# From flyways to foci: a systematic review and meta-analysis on the role of birds in the maintenance and global dispersal of ticks and tick-borne pathogens

**DOI:** 10.1186/s13071-025-07238-4

**Published:** 2026-01-24

**Authors:** Guo-Yao Zu, Wan-Nian Wei, Zhi Cao, Xiu-Tong Xiao, Hui-Jun Yu, Cheng Li, Shi-Jing Shen, Shuo Zhou, Ting-Ting Gong, Chen Shan, Wu-Chun Cao, Lin Zhao

**Affiliations:** 1 Institute of EcoHealth, School of Public Health, Cheeloo College of Medicine, Shandong University, Jinan, 250012 Shandong People’s Republic of China; 2 State Key Laboratory of Pathogen and Biosecurity, Beijing Institute of Microbiology and Epidemiology, Beijing, 100071 People’s Republic of China; 3 Shandong Provincial Key Laboratory of Intelligent Monitoring, Early Warning, Prevention and Control for Infectious Diseases, Jinan, 250012 Shandong People’s Republic of China

**Keywords:** Birds, Ticks, Tick-borne pathogens, Zoonoses, Bird migration

## Abstract

**Background:**

Birds (Aves) are considered to play important roles in the dissemination of ticks and tick-borne pathogens, yet the global extent of their contribution to vector maintenance and long-distance dispersal remains poorly quantified. This study provides a comprehensive global synthesis of bird-associated ticks (BATs) and bird-associated tick-borne pathogens (BATBPs) to characterize the epidemiological roles of birds and assess the resulting public health and biosecurity risks.

**Methods:**

We systematically searched multiple bibliographic databases and GenBank up to February 2025 in accordance with Preferred Reporting Items for Systematic reviews and Meta-Analyses (PRISMA) guidelines. Field-based studies reporting bird–tick–pathogen associations were included. Thematic maps showing the geographical distributions of birds, BATs, and BATBPs were produced in ArcGIS, and pooled infestation prevalence was estimated via logit-transformed random-effects meta-analysis with the Hartung–Knapp adjustment.

**Results:**

Our synthesis of 772 studies and 86 molecular records identified 185 BAT species and 102 BATBPs across 34 avian orders, representing 77.3% of all global orders. Within the BATBP spectrum, 53.9% are zoonotic, and 99 tick species have documented records of human-biting. Passeriformes (songbirds) hosted the greatest tick diversity (129 species), while Galliformes exhibited the highest pooled infestation prevalence (17.6%; *n* = 29 studies, *m* = 18,746 birds). Globally, allochthonous tick records showed relatively high spatial overlap with the Black Sea–Mediterranean and East Atlantic flyways. Critically, we identified a profound surveillance imbalance in Asia, which accounts for only 6.5% of sampling coordinates (26/397 sites) despite exhibiting a high diversity of emerging pathogens.

**Conclusions:**

Birds serve as important contributors to global tick-borne disease epidemiology through local vector maintenance and intercontinental bio-dispersal. They support tick feeding and life-cycle completion and may disperse ticks during migration, facilitating population establishment in new areas. Molecular evidence indicates that birds carry a broad spectrum of tick-borne pathogens; however, the available evidence is largely observational, and experimental validation is required to clarify reservoir competence and transmission. Strengthening integrated One Health surveillance of high-risk hubs, particularly in data-deficient regions such as Asia, is essential to mitigate spillover risk at shifting ecological and migratory interfaces.

**Graphical abstract:**

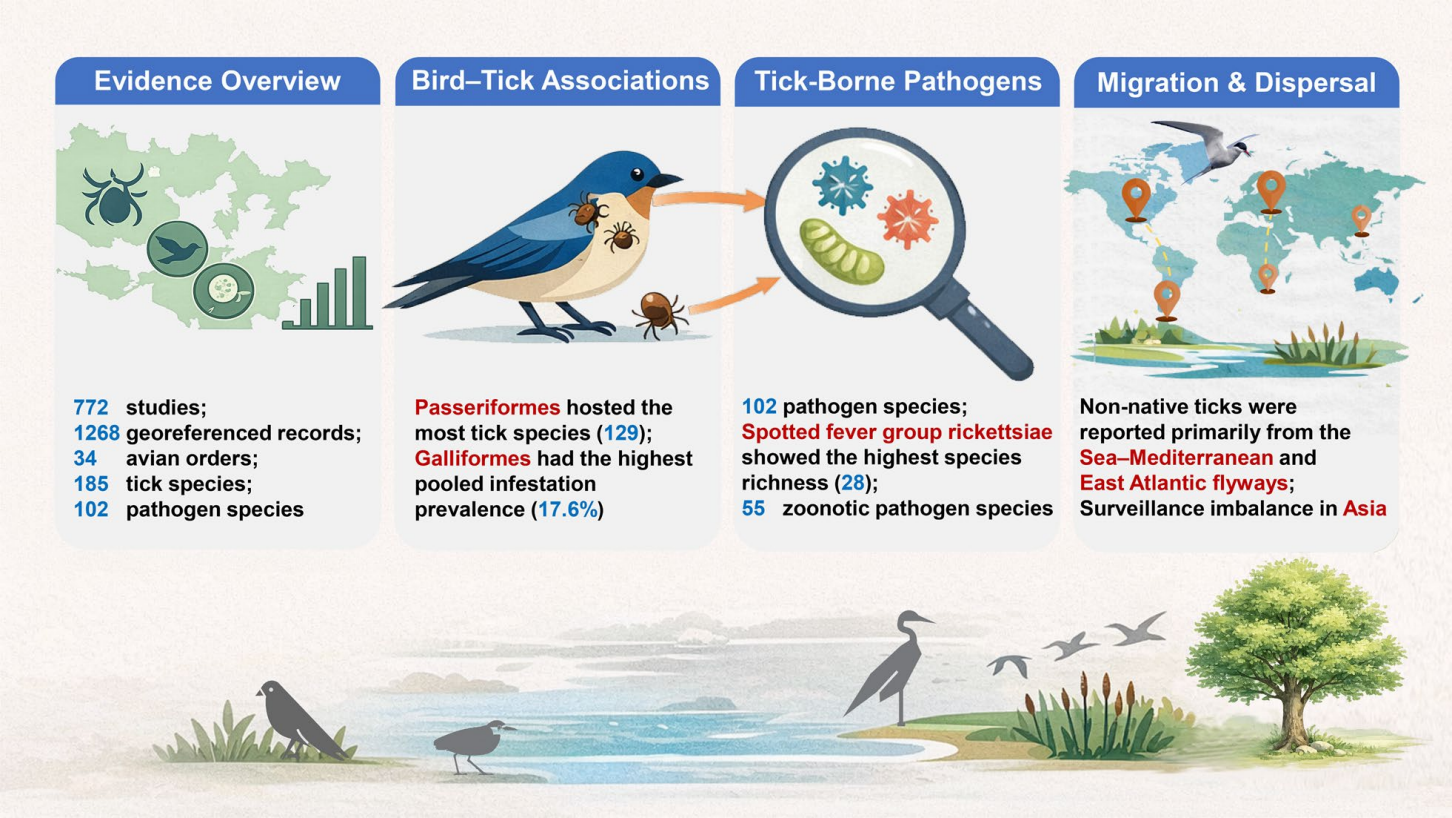

**Supplementary Information:**

The online version contains supplementary material available at 10.1186/s13071-025-07238-4.

## Background

Tick-borne diseases (TBDs) represent a primary and escalating global threat, imposing a substantial public health and economic burden [1]. The geographical expansion of certain tick populations [2, 3], coupled with the rising incidence of endemic and emerging TBDs [4–6], has raised significant concerns for human and animal health worldwide. Against this backdrop, birds (class Aves), as the most species-rich vertebrates with a worldwide distribution, have been inferred to play an important role as contributors to the dissemination of ticks and tick-borne pathogens [7–10]. However, evidence linking birds to these cycles remains fragmented [11]. Specifically, many studies do not clearly distinguish birds’ mechanical transport potential from reservoir competence, which constrains our understanding of how avian movements may contribute to the establishment of natural foci in novel regions.

In addition, the overall extent and global distribution of bird-mediated risks have not yet been systematically quantified. Existing reviews, for instance, are restricted to limited areas such as the Mediterranean basin and the Americas [12–14]. A global synthesis is therefore required to clarify the risks posed by bird-associated ticks (BATs) and bird-associated tick-borne pathogens (BATBPs) and to inform evidence-based guidance on diagnosis, surveillance, and control.

Our previous research suggested that the spread of *Haemaphysalis longicornis* in the USA might be associated with migratory birds [15] and further identified Aves as important hosts for *Haemaphysalis concinna*, supporting the view that they contribute to the dispersal and maintenance of tick populations across regions [16]. In this study, we conducted a comprehensive review, as follows: First, systematically characterize the global spatial distribution and taxonomic spectrum of BATs and BATBPs. Second, we analyzed tick infestation prevalence through the lens of avian ecological groups to better understand the potential human exposure risk and pathogen spillover risks. Finally, we mapped the distribution of non-native tick species detected along migratory flyways to evaluate the role of long-distance migration in seeding new geographical foci.

## Methods

### Study design and systematic search strategy

We conducted a systematic search of published literature across PubMed, Web of Science, Scopus, and the China National Knowledge Infrastructure (CNKI) up to February 16, 2025. The search followed the Preferred Reporting Items for Systematic Reviews and Meta-Analyses (PRISMA 2020) guidelines [17]; the PRISMA checklist and search strings are provided in Additional file 1 (Tables S1, S2). We implemented a broad search term strategy combining avian-related terms (e.g., birds, Aves, avian, migratory) with vector-specific terms (e.g., ticks, Acari, *Ixodes*, and all recognized tick genera). To minimize bias, no language restrictions were applied, and the search included primary research articles while excluding conference abstracts and gray literature. All studies were imported into EndNote (version 21.2.0.17387) and Microsoft Excel 2021 for automated and manual deduplication.

### Literature screening and eligibility criteria

We implemented a two-stage screening process. Inter-reviewer agreement was quantified using Cohen’s kappa (κ) statistic [18]. Two reviewers (G-YZ and W-NW) independently screened titles/abstracts and full texts, with any discrepancies resolved through discussion or by consulting a third reviewer (LZ). Eligibility criteria were defined to disentangle the roles of birds in vector maintenance versus pathogen transport: (1) field-based studies reporting ticks collected directly from birds (feeding ticks), from identifiable nests/cavities (questing/nidicolous ticks), or recorded as prey; (2) molecular or serological detection of pathogens in bird tissues or their associated ticks; (3) provision of location data suitable for georeferencing. Studies reporting only tick infestation without pathogen detection were still included for BAT diversity and prevalence analyses. We excluded reviews, theoretical studies, laboratory-based experimental trials, veterinary clinical reports, and publications lacking original field data (Additional file 1: Table S3).

### Data extraction and standardization

We extracted variables including country/location, habitat type, sampling period, avian species, tick species, pathogen species, pathogen detection methods, number of birds examined and infested (for meta-analysis), and geographical coordinates (Additional file 1: Table S4).

To ensure data consistency and accuracy, avian nomenclature was standardized according to the International Ornithological Committee (IOC) World Bird List (http://www.worldbirdnames.org/) (version 14.2, accessed December 18, 2024). Seven ecological groups were categorized based on avian ecological features [7] to better reflect tick-acquisition risk beyond simple taxonomy: songbirds, raptors, landfowl, shorebirds, waterfowl, climbing birds, and aerial birds (Additional file 1: Table S5).

Tick and pathogen nomenclature were verified and unified against the National Center for Biotechnology Information (NCBI) Taxonomy Database (https://www.ncbi.nlm.nih.gov/taxonomy), Global Biodiversity Information Facility (GBIF, https://www.gbif.org/) and the International Committee on Taxonomy of Viruses (ICTV) taxonomy (https://ictv.global/taxonomy) (all accessed February 2025). To reduce epidemiological noise and ensure data quality, tick-associated symbionts (e.g., *Coxiella*-like endosymbiont) were systematically excluded from the BATBP dataset, and genus-level records (e.g., *Ixodes* sp.) were treated as independent taxa only if no other species from that genus were identified in the same study to avoid potential double-counting. Detailed methods are provided in Additional file 1 (Text S1).

All sampling coordinates were standardized to decimal degrees to enable high-resolution spatial analysis. Whenever available, we extracted geocoordinates directly from peer-reviewed manuscripts; when only administrative units or bounded sampling regions (e.g., counties, cities, provinces, or custom sampling areas) were reported, we used the geographical centroid of that polygon as an approximate point location. This differentiation in coordinate resolution is maintained in the spatial database to ensure transparency. Additionally, to maximize the coverage of global pathogen distributions, we interrogated the GenBank database (https://www.ncbi.nlm.nih.gov/genbank/) (accessed February 2025), downloaded pathogen records explicitly derived from birds or BATs, and extracted the corresponding location information for inclusion in the BATBP dataset.

### Spatial mapping and statistical analysis

Thematic maps were generated using ArcGIS (version 10.8; Esri, Redlands, CA, USA) to illustrate the global occurrence of birds, BATs, and BATBPs. Each georeferenced point represents an independent detection event from the peer-reviewed dataset. GenBank-derived records—which often lack comprehensive denominator data—were summarized only at the country level in a separate illustrative map and were strictly excluded from the prevalence meta-analyses. Rose diagrams were generated in R (version 4.3.3) using tidyverse to visualize tick composition by continent.

We employed meta-analysis to estimate the pooled infestation prevalence (defined as the proportion of birds infested with ticks) and its corresponding 95% confidence intervals (CIs). To ensure the robustness of our estimates and minimize the impact of small-sample bias, studies were included in the meta-analysis only if they reported the exact numbers of avian hosts examined and infested; studies with a total sample size of fewer than 10 individuals were excluded. Furthermore, to maintain statistical validity, we pre-specified that no pooled estimate would be calculated when only a single study was available for a specific category or avian order.

Meta-analyses were performed in R (version 4.3.3) using the meta package. Prevalence was pooled using a logit-transformed random-effects model, with τ^2^ estimated by the DerSimonian–Laird method and 95% CIs computed using the Hartung–Knapp adjustment. Heterogeneity was quantified using *I*^2^, and a 95% prediction interval (PI) was reported for each pooled estimate. All subgroup analyses (by ecological group, continent, season, and habitat) consistently utilized the random-effects model to ensure methodological comparability across groups.

## Results

### Literature search and data composition

**Fig. 1:**
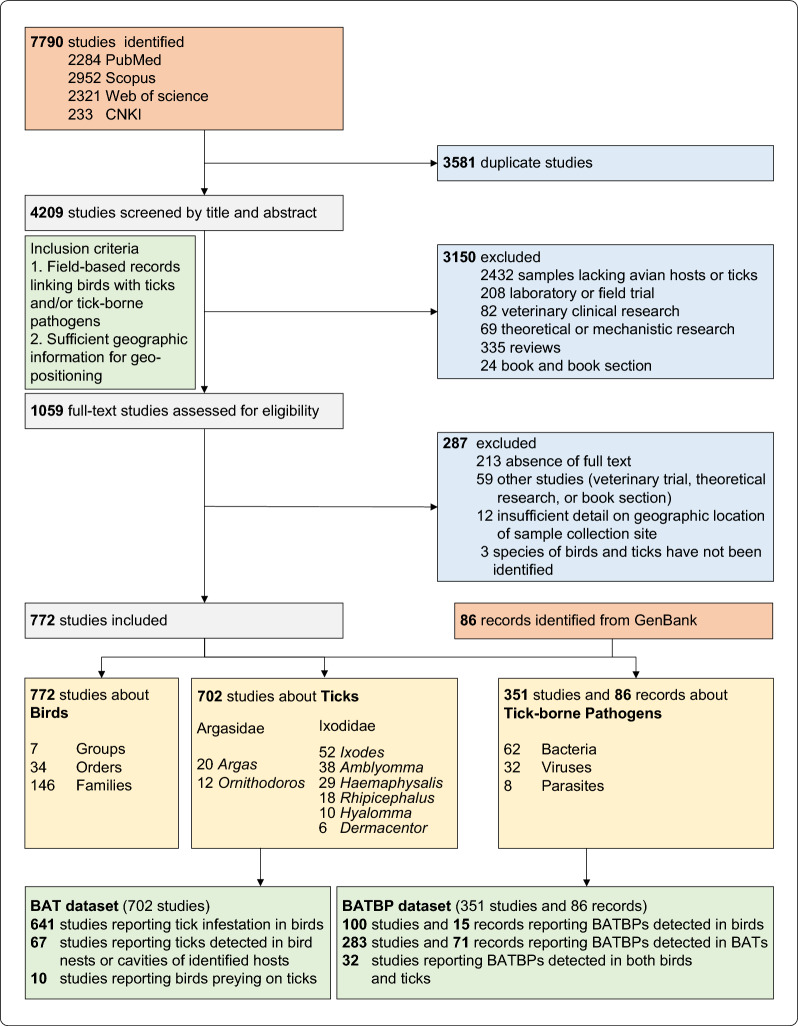
Flow diagram of the systematic review process. Literature and database searches were conducted up to Feb 16, 2025. *CNKI* China National Knowledge Infrastructure, *BATBPs* bird-associated tick-borne pathogens, *BATs* bird-associated ticks

Our systematic search and screening process is detailed in Fig. 1. The initial search identified 7790 studies. After a rigorous two-stage screening process, 772 original studies were included (Additional file 2: Dataset S1). Inter-reviewer agreement during study selection was substantial (Cohen’s κ = 0.76, 95% CI 0.73–0.78), indicating high reliability in the selection process. In addition, 86 records were identified from the GenBank database. All 772 studies contributed data on birds covering 34 avian orders and 146 families. A subset of 702 studies reported tick-related data (the BAT dataset), identifying 185 tick species belonging to eight genera (Additional file 2: Dataset S2). Most tick-related studies investigated tick infestation in birds, accounting for 641 of 702 studies (91.3%). Pathogen information was extracted from 351 included studies and 86 GenBank records (the BATBP dataset), yielding 102 distinct BATBP species, comprising 62 bacteria, 32 viruses, and eight parasites (Additional file 2: Dataset S3).

### Global spatial distribution of birds, BATs, and BATBPs

**Fig. 2:**
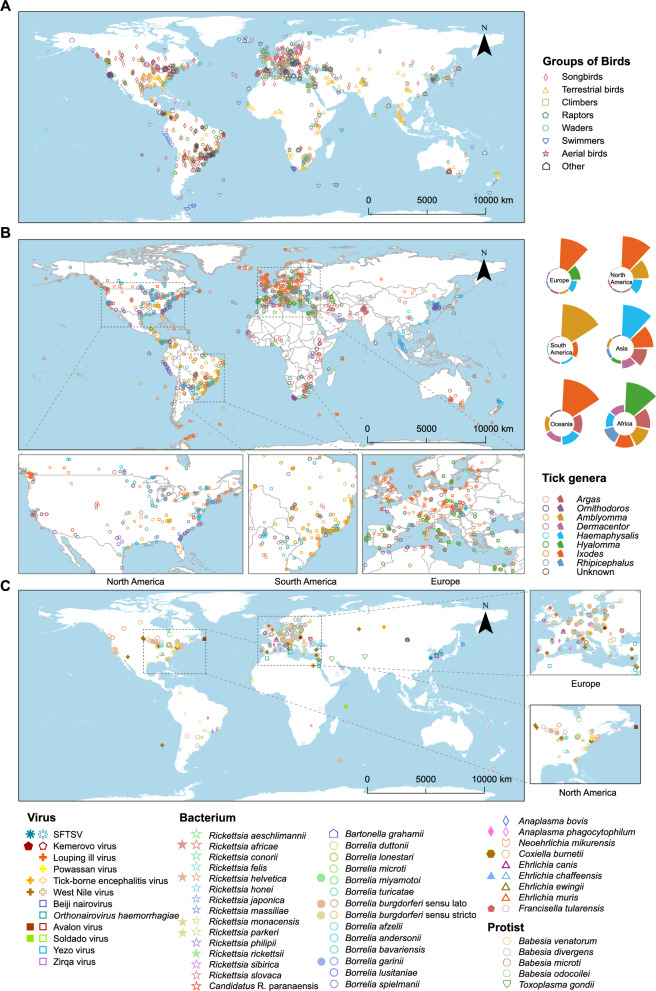
Global distribution of birds, bird-associated ticks (BATs), and bird-associated tick-borne pathogens (BATBPs). **A** Georeferenced locations of birds, classified by ecological group. **B** Georeferenced locations of BATs. Rose diagrams illustrate the composition of tick genera by continent, where the length of each semicircle is proportional to the number of records. **C** Georeferenced locations of zoonotic BATBPs. Solid symbols indicate pathogen detections in avian samples; hollow symbols indicate detections in tick samples. *SFTSV* severe fever with thrombocytopenia syndrome virus

We identified 1268 georeferenced records of birds and BATs across 96 countries and 397 georeferenced pathogen detection sites across 79 countries. As shown in Fig. 2A, records of birds with documented tick contact were geographically concentrated in Europe (391/1268, 30.8%), North America (317/1268, 25.0%), and South America (259/1268, 20.4%). When classified by ecological group, records were most frequent for songbirds (730/1268, 57.6%), followed by landfowl (255/1268, 20.1%) and shorebirds (187/1268, 14.8%). Notably, even aerial birds such as Apodiformes, which spend most of their lives in flight, were reported to be parasitized by ticks. Detailed results are provided in Additional file 3 (Fig. S1, S2).

Marked differences were observed in the geographical distribution of tick genera (Fig. 2B). *Ixodes* exhibited the broadest geographical distribution (678/1268, 53.5%), dominating reports from Europe (323 coordinates) and North America (214). *Amblyomma* (320/1268, 25.2%) was recorded mainly in South America (185), while *Haemaphysalis* (261/1268, 20.6%) was the most frequently reported genus in Asia (57). In Africa, although *Argas* showed the widest geographical range, *Hyalomma* was more frequently reported, and our results indicate that it was associated with a broad range of avian orders (*n* = 20). These findings suggest continent-specific patterns in the dominant tick genera. More details in the rose diagram are provided in Additional file 3 (Figs. S3–S5, Table S6).

We mapped 268 unique coordinates for 55 species of zoonotic BATBPs (Fig. 2C, Additional file 3: Table S7). Bacterial pathogens were the most diverse (37 species) and widely distributed (223/271, 82.3%), with *Borreliaceae* being the most frequent (152/271, 56.1%). Viruses (e.g., *Orthonairovirus* and *Orthoflavivirus*) were detected almost exclusively in the Northern Hemisphere. Critically, we identified a significant surveillance imbalance: Asia exhibited a comparatively high pathogen richness (21 species) relative to its sparse sampling effort (26 coordinates), suggesting that many Asian foci remain under-investigated. The geographical distribution of non-zoonotic pathogens and GenBank-derived records is provided in Additional file 3 (Figs. S6, S7).

### Diversity and infestation prevalence of BATs

**Fig. 3:**
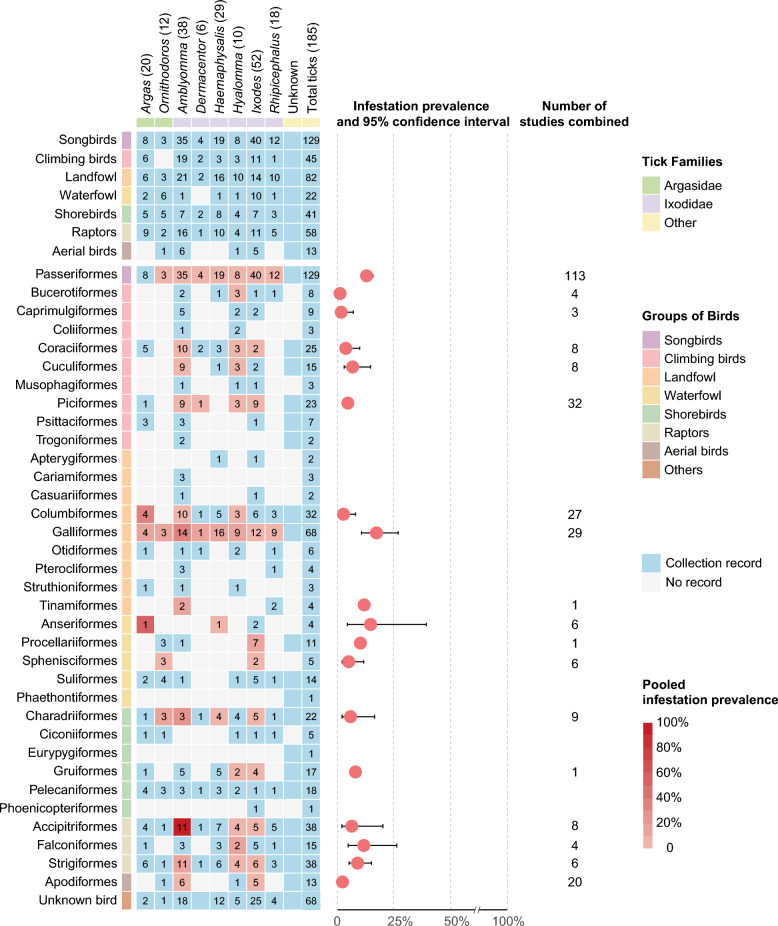
Diversity of BATs and pooled infestation prevalence. The left panel is a diversity matrix illustrating associations between avian orders (rows) and tick genera (columns). The avian orders are also classified by their ecological group (labels on the far left). Column headers indicate the total number of tick species per genus, and matrix cells show the number of species recorded for each avian order. The intensity of the red shading corresponds to the pooled infestation prevalence for each avian order–tick genus combination. Pale blue cells indicate combinations with insufficient data for meta-analysis. The right panel displays the overall pooled tick infestation prevalence (with 95% CI) for each avian order

Bird–tick associations involved 34 avian orders and 185 tick species (Fig. 3) and comprised three main evidence contexts: (i) 176 tick species collected directly from bird bodies, (ii) engorged or questing/free-living ticks collected from identifiable host bird nests/cavities, and (iii) records of tick consumption by birds (Additional file 4: Fig. S8).

Passeriformes hosted the greatest tick diversity (129 species), dominated by *Ixodes* (40) and *Amblyomma* (35). Galliformes (including domestic poultry and wild landfowl) were associated with 68 tick species, mainly *Haemaphysalis* (16) and *Amblyomma* (14). Several other orders also showed high diversity, for example, Accipitriformes, Falconiformes, and Strigiformes (collectively, raptors), together were associated with 58 tick species.

Meta-analyses provided pooled infestation prevalence estimates with corresponding study and sample counts to contextualize the data. Among all avian orders, Galliformes exhibited the highest pooled prevalence (17.6%; *n* = 29 studies, *m* = 18,746 birds), followed by Anseriformes (14.8%; *n* = 6 studies, *m* = 304 birds) and Passeriformes (12.9%; *n* = 113 studies, *m* = 278,857 birds) (Fig. 3; Additional file 4: Table S8). In specific avian order–tick genus combinations, we noted extremely high estimates derived from limited study counts, which require cautious interpretation: *Amblyomma* infestation in Accipitriformes (91.7%; *n* = 1 study, *m* = 12 birds) and *Argas* infestation in Anseriformes (54.3%; *n* = 2 studies, *m* = 84 birds) (Additional file 4: Table S9, Fig. S9). Subgroup analyses indicated that infestation was highest in summer (17.0%; *n* = 34 studies, *m* = 55,031 birds). By habitat, infestation prevalence was higher in woodlands (14.8%; *n* = 84 studies, *m* = 125,505 birds) and agro-pastoral landscapes (13.9%; *n* = 25 studies, *m* = 10,170 birds); detailed results are provided in Additional file 4 (Table S10).

### Diversity and linkage of BATBPs

**Fig. 4:**
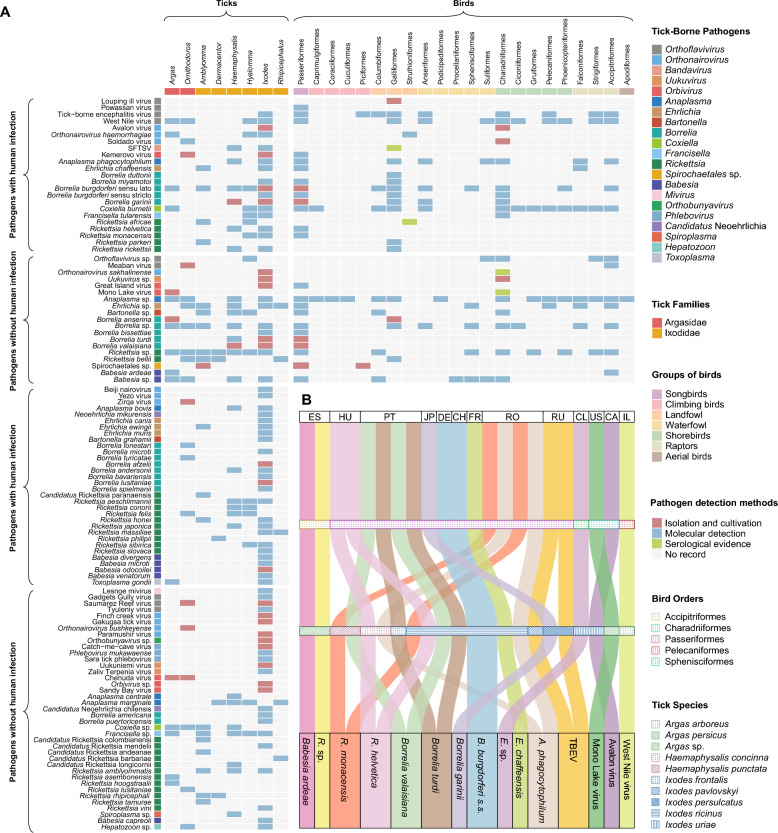
Diversity and linkage of tick-borne pathogens associated with birds. **A** A diversity matrix chart showing pathogens (rows) detected in tick genera and avian orders (columns). To reflect evidentiary confidence and ensure detection specificity, cell colors follow a hierarchical priority—isolation and culture, followed by molecular detection, and then serology—with microscopy-only records excluded. **B** A Sankey diagram. Link widths are proportional to the number of co-detection events for each specific pathogen extracted from the literature. ES: Spain; HU: Hungary; PT: Portugal; JP: Japan; DE: Germany; CH: Switzerland; FR: France; RO: Romania; RU: Russian Federation; CL: Chile; US: United States of America; CA: Canada; IL: Israel; TBEV: Tick-borne encephalitis virus; SFTSV: Severe fever with thrombocytopenia syndrome virus; *A. phagocytophilum: Anaplasma phagocytophilum*; *E. chaffeensis*: *Ehrlichia chaffeensis; E.* sp.: *Ehrlichia* sp.; *B. burgdorferi* s.s.: *Borrelia burgdorferi* sensu stricto; *R. helvetica*: *Rickettsia helvetica*; *R. monacensis*: *Rickettsia monacensis*; *R.* sp.: *Rickettsia* sp.

The BATBP dataset encompassed 102 pathogen species from 21 genera, of which 55 species (53.9%) are recognized as human pathogens. The diversity matrix (Fig. 4A) showed that *Ixodes* accounted for the largest pathogen spectrum (69 species), including 20 species identified via isolation or culture. Across avian orders, the largest numbers of BATBP species were reported in Passeriformes (at least 20), followed by Charadriiformes (18) and Galliformes (15). Within the zoonotic subset, bacteria predominated (37 species, 67.3%), with spotted fever group rickettsiae (SFGR) representing the most diverse bacterial cluster (14 species).

To move beyond simple pathogen occurrence records, we summarized co-detection records defined as the concurrent detection of the same pathogen in avian samples and in associated ticks—collected either directly from avian hosts or from their identifiable nests/cavities—within the same study, site, and sampling period (Fig. 4B). Such associations were reported in 13 countries across eight pathogen genera, including *Orthoflavivirus*, *Orthonairovirus*, *Rickettsia*, *Borrelia*, and *Ehrlichia*. In the 23 co-detection records, ticks were reported as parasitizing or engorged in 22 (95.7%). In addition, 15 records (65.2%) explicitly reported the PCR target genes used for detection (Additional file 5: Table S11). Notably, matching gene sequences of *Ehrlichia chaffeensis* were identified in tissues of *Erithacus rubecula* and in its attached *Ixodes ricinus* ticks. Although the direction of transmission cannot be inferred, this paired detection provides stronger evidence that pathogen occurrence is directly linked to the bird–tick interface, rather than to other potential sources.

### Local and global roles of birds in tick and pathogen cycles

**Fig. 5:**
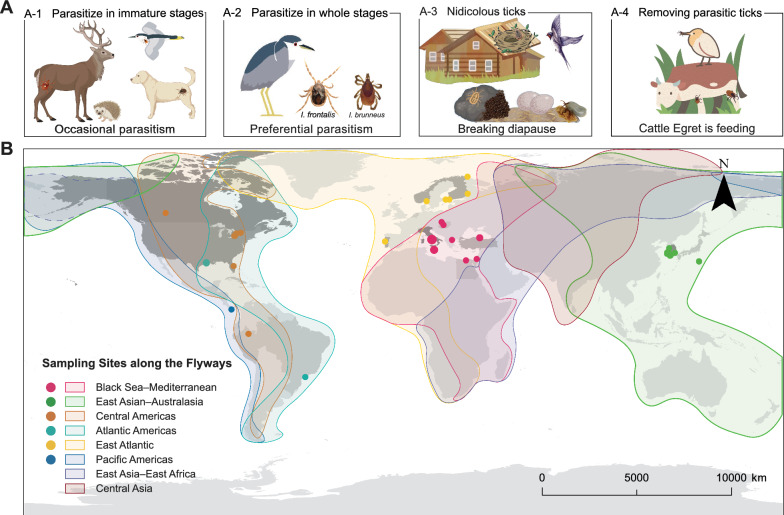
Ecological mechanisms for the local maintenance and global dispersal of ticks by birds. **A** Schematic illustrating the complex ecological relationships between birds and ticks at the local scale, including generalist parasitism (**A-1**), specialist ornithophilic parasitism (**A-2**), nidicolous (nest-based) cycles (**A-3**), and avian tick predation (**A-4**). **B** Global map showing the recorded occurrences of non-native (allochthonous) tick species detected on migratory birds, overlaid on major intercontinental flyways. Allochthonous ticks were identified using study-reported “first record/non-native” statements, and were cross-validated against GBIF and the literature at the first administrative level to grade records as confirmed or suspected. The basemap’s color gradient indicates the number of non-native tick species recorded per country

We synthesized existing evidence of bird-mediated ecological processes at two scales (Fig. 5). At the local scale, bird–tick interactions manifest through three primary ecological pathways: (1) opportunistic generalist parasitism, where broad host-range ticks (e.g., *I. ricinus*) utilize birds as incidental hosts, with larvae and nymphs being acquired through direct environmental contact during ground-foraging (Fig. 5A-1); (2) specialized ornithophilic parasitism, involving at least 49 species (e.g., *Ixodes frontalis*) that rely on birds across all life stages to maintain stable maintenance cycles (Fig. 5A-2; Additional file 6: Fig. S10, Table S12); and (3) nidicolous cycles, where approximately 27 species are sequestered within nests or cavities, with life histories synchronized to seasonal host occupancy (Fig. 5A-3; Additional file 6: Table S13). Conversely, we identified an opposing role where some species, such as the cattle egret (*Bubulcus ibis*), act as active predators (Fig. 5A-4; Additional file 6: Table S14). Nevertheless, the confirmation of 99 human-biting BAT species and 20 species infesting poultry underscores a substantial risk of pathogen spillover (Additional file 6: Tables S15, S16).

At the global scale, we synthesized 33 studies across 13 countries documenting the introduction of allochthonous (non-native) ticks by migratory birds (Fig. 5B, Additional file 6: Table S17). These records were geographically clustered along the Mediterranean coast of Europe, the Atlantic seaboard of the Americas, and East Asia, showing high spatial concordance with the Black Sea–Mediterranean and East Atlantic flyways. By cross-referencing these detections with national endemic checklists and GBIF occurrence records, we assessed the confidence level of the “allochthonous” tick records reported in our results. In contrast, the East Asia–East Africa and Central Asia flyways remain critically data-deficient, with no published reports of allochthonous tick introductions identified within the search period (up to February 2025).

## Discussion

Wild birds frequently host ticks, including ticks infected with tick-borne pathogens [19], and may contribute to human and animal exposure in some settings [20]. However, evidence remains fragmented across regions and taxa, with a historical reliance on local observational data. To our knowledge, this is the most comprehensive systematic review to date, synthesizing available published evidence up to February 2025 on the global diversity, spatial distribution, infestation prevalence, and ecological associations of birds, BATs, and BATBPs.

We observed that dominant BAT genera vary by continents, with *Haemaphysalis* predominating in Asia and *Ixodes* in Europe (Fig. 2B). This spatial sorting is epidemiologically significant because different tick taxa vector distinct pathogen spectra; for instance, *Ha. longicornis* is often linked to SFGR outbreaks in East Asia [15], whereas *Ixodes persulcatus* is associated with high *Babesia* diversity in northern latitudes [21]. The currently documented BATBP detections also displayed strong geographical clustering (Fig. 2C). Europe and North America contributed geographically widespread reports of BATs and BATBPs, whereas Asia showed comparatively high BATBP diversity despite relatively sparse sampling. In contrast, data from Africa and Oceania remain limited, likely reflecting regional disparities in surveillance intensity and diagnostic capacity rather than a lack of biological risk [22]. These findings underscore the need for regional surveillance strategies that account for local bird and tick species present.

By synthesizing evidence across 34 avian orders (77.3%, 34/44, according to the IOC classification [23]), our review broadens the current understanding of the avian tick spectrum [24], indicating that birds have been documented as hosts for at least 176 BAT species. Previous studies indicate that ground-foraging species are disproportionately associated with higher tick-infestation prevalence and intensity [13]. In this study, our meta-analysis identified Galliformes, including domestic poultry and wild terrestrial birds, as the order with the highest pooled infestation prevalence (17.6%; *n* = 29 studies, *m* = 18,746 birds). Passeriformes, which are common in peri-urban and agricultural settings and frequently overlap with human activities [25], accounted for the greatest reported tick diversity (129 species), in line with previous reports [26]. These findings suggest that BATs may pose a public health risk, given the potential for human exposure. In addition, ecological traits, such as the potential for parthenogenesis in *Ha. longicornis* [27], further exacerbate introduction risks, as a single female tick can establish a new population in a novel habitat [28]. Birds can also influence nidicolous ticks by disrupting diapause, facilitating life-cycle completion, and supporting the long-term maintenance of natural foci within nesting microhabitats [29]. Although standardized bird-focused tick sampling is challenging [10], these findings support broader and more systematic surveillance of birds across habitats, particularly where close contact with people or livestock is likely.

Several studies suggest that wild birds may serve as hosts for specific tick-borne agents in certain ecological contexts [30–33]. In our review, the BATBP reservoir appears broader than commonly recognized. Among these, 53.9% (55/102) of BATBP species were identified as human pathogens. These include globally significant agents such as SFGR [34], *Anaplasmataceae* [35], and the *Borrelia burgdorferi* sensu lato complex [36]. We also identified reports linking birds to viruses of public health concern, such as Powassan virus [37], the emerging severe fever with thrombocytopenia syndrome virus (SFTSV) endemic in East Asia, and West Nile virus (WNV). However, tick vector competence for WNV remains to be clarified [38]. Notably, simple pathogen presence in birds does not prove reservoir competence [39]. In most cases, pathogens detected in birds and in their associated ticks did not match [40]. We therefore prioritized within-study co-detection evidence, specifically instances of molecular identity where identical gene sequences were found in avian tissues and attached feeding ticks [41]. Such findings move beyond simple presence records and offer preliminary evidence that is consistent with the possibility of tick-mediated bridging of pathogens between birds and other hosts within shared ecological settings. However, co-detection does not resolve transmission directionality, and which BATBPs are efficiently transmitted between birds and ticks ultimately requires experimental validation [42].

The emergence of new natural foci reflects a complex interplay of ecological and anthropogenic drivers and can be strongly shaped by host movements, given the limited active dispersal of ticks [43, 44]. Quantitative appraisals now suggest that between 4 and 39 million Neotropical ticks are transported annually into North America via songbird migration [13]. In this review, we adopted two complementary views to consider how birds may contribute to the maintenance and geographical redistribution of ticks and tick-borne pathogens. In a local-scale view (Fig. 5A), birds act as principal hosts for at least 49 specialist ornithophilic tick species (e.g., *I. frontalis*) that are closely associated with avian hosts across multiple developmental stages [45]. From a global-scale view, migratory birds undertake repeated stopovers during which they may acquire ticks or drop engorged ticks [46]. Based on the available literature, we integrated the reported detection locations of non-native BATs with their previously documented geographical ranges and found that their apparent dispersal patterns may align with major avian migratory flyways, such as the Black Sea–Mediterranean and East Atlantic flyways [47, 48]. Given the strong host [49, 50] and ecological [51] adaptations of some tick species, birds may have played an important role in facilitating their dispersal. Therefore, strengthened surveillance using standardized protocols is warranted, particularly in Asian foci and other data-sparse regions.

Our study has several limitations. First, our review was largely restricted to Chinese and English literature, and relevant studies in other languages may have been missed. Second, field reporting quality varies significantly across regions, and observed patterns are influenced by sampling and publication biases. Finally, the classification of migratory status is complex, and the lack of fine-scale movement data limited our ability to conduct specific analyses on individual migratory cohorts.

## Conclusions

Synthesizing evidence from 772 primary studies and 86 molecular records, our review establishes birds as important contributors to the global epidemiology of TBDs. Birds can support tick feeding and, for ornithophilic ticks in particular, facilitate completion of the tick life cycle. At the same time, the transport role of birds should not be overlooked, as birds can carry infected ticks into new areas and may contribute to the establishment of new foci. While substantial molecular detection evidence exists, the reservoir competence of most avian hosts remains a critical knowledge gap and requires formal experimental validation. Overall, the pronounced surveillance imbalance in Asia, major data deficiencies along key flyways, and the high statistical heterogeneity inherent in global ecological reports represent quantifiable gaps in the current evidence base. Prioritizing integrated One Health monitoring of high-risk bird groups is essential to bridge these gaps and mitigate pathogen spillover risk across shifting ecological and migratory boundaries.

## Supplementary Information

Additional file 1: Text S1. Supplementary Methods. Text S2. Supplementary Results. Table S1. PRISMA 2020 Checklist. Table S2. The detailed search strategy for each database. Table S3. The inclusion and exclusion criteria of screening publications. Table S4. List of variables extracted from reviewed studies. Table S5. The classification definition of the seven ecological groups of wild birds.

Additional file 2: Dataset S1. Supplementary References. Dataset S2. Dataset of bird-associated ticks. Dataset S3. Dataset of bird-associated tick-borne pathogens.

Additional file 3: Figure S1. Global distribution of birds associated with ticks. Figure S2. Continental distribution and composition of bird groups. Figure S3. Global distribution of ornithophilic ticks. Figure S4. Global distribution of BATs. Figure S5. Continental distribution and composition of BATs. Table S6. Number of bird–tick reports by continent. Table S7. Zoonotic BATBPs. Figure S6. Global distribution of non-zoonotic BATBPs. Figure S7. Global distribution of BATBPs (GenBank).

Additional file 4: Figure S8. Relationship matrix between tick species and avian. Table S8. Estimated tick infestation prevalence. Table S9. Infestation prevalence as reported in individual studies. Figure S9. Meta-analysis of tick infestation prevalence by tick genus across avian orders. Table S10. Subgroup analysis of tick infestation prevalence.

Additional file 5: Table S11. Co-detection of BATBPs in birds and BATs within the same study.

Additional file 6: Figure S10. Composition of ornithophilic ticks. Table S12. Distribution, hosts, and pathogens of ornithophilic ticks. Table S13. Ticks collected from bird nests or nesting cavities. Table S14. Confirmed records of tick predation by birds. Table S15. Ticks with evidence of biting humans. Table S16. Poultry infested with ticks. Table S17. Non-native ticks along migratory bird flyways.

## Data Availability

All data generated or analyzed during this study are included in this article and its supplementary files; the full dataset, including geographical coordinates and other relevant fields, is provided in Additional file 2.

## Abbreviations

TBDs: Tick-borne diseases
BAT: Bird-associated tick
BATBP: Bird-associated tick-borne pathogen
PRISMA: Preferred Reporting Items for Systematic reviews and Meta-Analyses
CI: Confidence interval
PI: Prediction interval
CNKI: China National Knowledge Infrastructure
IOC: International Ornithological Committee
ICTV: International Committee on Taxonomy of Viruses
NCBI: National Center for Biotechnology Information
Cohen’s κ: Cohen’s kappa
SFGR: spotted fever group rickettsiae
SFTSV: Severe fever with thrombocytopenia syndrome virus
WNV: West Nile virus
