# Supplementary material for: From flyways to foci: a systematic review and meta-analysis on the role of birds in the maintenance and global dispersal of ticks and tick-borne pathogens
Source: Parasit Vectors. 2026 Jan 24;19:88. doi: 10.1186/s13071-025-07238-4 (PMC12914891; doi:10.1186/s13071-025-07238-4)
Supplement: Supplementary file 1 — Additional file 1: Text S1. Supplementary Methods. Text S2. Supplementary Results. Table S1. PRISMA 2020 Checklist. Table S2. The detailed search strategy for each database. Table S3. The inclusion and exclusion criteria of screening publications. Table S4. List of variables extracted from reviewed studies. Table S5. The classification definition of the seven ecological groups of wild birds. [file 13071_2025_7238_MOESM1_ESM.docx]

Text S1. Supplementary Methods

***Avian taxonomy***

We standardized avian names according to the IOC World Bird List (version 14.2, accessed Dec 18, 2024). All bird species names reported in the primary studies were matched to the IOC list, and historical or vernacular names were updated to the corresponding valid species names. When only common names were provided in the original articles, we used BirdLife International and IOC crosswalks to identify the corresponding scientific names. Records that could not be confidently assigned to a unique IOC species (e.g. ambiguous common names) were excluded from species-level analyses but retained at higher taxonomic levels (order or family).

***Tick nomenclature and standardisation***
Tick names were first extracted exactly as reported in each primary study and then standardised in a stepwise manner. We used the NCBI Taxonomy database (accessed February 2025) as the primary reference to update spelling, synonymies and current combinations, and cross-checked doubtful or missing entries against additional authoritative sources (e.g. GBIF backbone taxonomy and recent global or regional tick catalogues/monographs). When a reported species name could not be validated or is not currently accepted (e.g. Argas zumpti), we adopted a conservative approach and retained the record at the genus level in all quantitative analyses (e.g. Argas sp.).

When counting species, genus-only records (e.g., *Ixodes* sp.) were retained for genus-level summaries but were not treated as additional species if one or more congeneric species were already identified in the same study, to avoid inflating species counts. For example, records reporting *Ixodes pavlovskyi*, *Ixodes turdus*, and *Ixodes* sp. were summarized as at least two *Ixodes* species.

We defined an occurrence as allochthonous (non-native) when the original study explicitly described a first local record or a non-native tick and reported species identification methods; each candidate record was then cross-validated using GBIF occurrence data and supplementary PubMed searches, and classified as confirmed or suspected (Additional file 6: Table S17).

***Taxonomic standardization of Lyme borreliosis group spirochetes***

In this study, we followed the traditional usage of *Borrelia* sensu lato for the Lyme borreliosis group, in line with the treatment of *Borreliella* as a heterotypic synonym of *Borrelia* in major nomenclatural esources [1]. Accordingly, all sequence records and published names labelled as “*Borreliella*” in NCBI or in primary studies were harmonized under *Borrelia* sensu lato in our dataset.

***Standardization of pathogen detection methods in the reviewed studies***

We standardized laboratory evidence into four categories: molecular detection (PCR or sequencing of genus or species-specific targets), isolation and cultivation (culture or isolation with confirmatory PCR or sequencing when reported), light micrograph identification (morphological identification in stained smears or tissues), and serological tests (e.g., IFA or ELISA, including paired-serum seroconversion where available).

***Geo-positioning of the occurrence data***

Whenever available, we extracted geocoordinates (decimal latitude and longitude) from peer-reviewed articles reporting bird-associated ticks or tick-borne pathogens. These coordinates were recorded as reported by the authors.

When point information was not available, we extracted the location as a two-dimensional bounded region, or a “polygon” [2]. A polygon was usually an dministrative unit, such as a county, a city or a province, but could also be a customized sampling region defined by the study (e.g. a specific forest transect or island area). For each polygon, the coordinates of its geographic centroid were queried from Google Maps or equivalent online gazetteers and used as an approximate point location representing that sampling region.

**Reference**

1. LPSN – List of Prokaryotic names with Standing in Nomenclature. Leibniz Institute DSMZ. Available from: https://lpsn.dsmz.de/ (accessed February 2025).
2. Tang T, Zhu Y, Zhang YY, et al. The global distribution and the risk prediction of relapsing fever group Borrelia: a data review with modelling analysis. Lancet Microbe. 2024;5:e442-e451.

Text S2. Supplementary Results

**Global distribution of birds parasitized by ticks**

The seven avian groups showed distinct global distribution patterns (Figure 1). Songbirds, represented solely by the order Passeriformes, had the widest geographical range and the highest species richness. Landfowl formed a nearly continuous distribution across temperate and semi-arid regions, with Galliformes recorded from all continents except Antarctica and Columbiformes concentrated in mid-latitude Eurasia and Latin America. Among water-associated groups, Charadriiformes dominated the wader group, occurring widely along northern-hemisphere coastlines, particularly in Europe and North America. In the raptor group, Accipitriformes were the most representative taxon, whereas within the climbing birds, Piciformes and Coraciiformes exhibited the broadest distributions.

**Global distribution of ticks**

A total of 1268 georeferenced records of birds and bird associated ticks (BATs), representing 204 species from 8 genera, were compiled to examine global distribution patterns. To further investigate genus-specific spatial trends, we compared the distribution maps of ornithophilic ticks and those collected from birds that are only occasionally parasitized. Ornithophilic ticks were identified in five of the eight genera, encompassing 48 species in total. Host preference was most pronounced in the genus *Argas*, for which all 20 recorded species were ornithophilic, followed by *Ornithodoros*, in which most species (seven/twelve) belonged to this category.

Comparative analysis of spatial distributions revealed distinct geographical patterns across major genera. The widespread genus *Ixodes* (53.5%, 678/1268) generally showed overlapping distributions between ornithophilic and occasionally parasitizing species; however, its ornithophilic members extended markedly toward polar regions. This poleward expansion was primarily driven by *I. uriae*, a seabird-associated tick recorded on penguins, enabling the genus’ occurrence in polar and subpolar zones. *Haemaphysalis* (261/1268; 20.6%) ranked second in total records but contained relatively few ornithophilic species, whose distribution largely coincided with that of ticks from occasionally parasitized birds. The detailed geographic distribution of BATs is provided in Additional file 3: Fig. S3-5.

***Global distribution of non-zoonotic BATBPs***

In addition, 47 bird-associated tick-borne pathogens (BATBPs) without confirmed human pathogenicity were identified. By pathogen type, bacterial BATBPs exhibited the greatest species richness, with 30 species across six major genera showing distinct geographical segregation. Members of the order *Rickettsiales* and related groups were dominant, comprising 11 species mainly distributed across Europe and the Americas. Viral BATBPs, primarily belonging to the genera *Orthoflavivirus* and *Orthonairovirus*, were mostly recorded in Europe but were also sporadically detected in coastal and island regions such as Madagascar, islands off southeastern Australia, and areas near the Kamchatka Peninsula. Parasites were mainly detected in Europe but occasionally occurred in mid- to high-latitude coastal regions of the Southern Hemisphere, including New Zealand, the Cape region of Africa, and sites near the Drake Passage. The detailed geographic distribution of BATBPs is provided in Additional file 3: Fig. S6, S7.

***Results of subgroup analysis***

Based on stratification by avian habits/ecological groups, continent, climate zone, season, and habitat at sampling sites, the subgroup analyses showed that songbirds had the highest tick‐infestation prevalence at 12.9% (107 studies; 275707 birds). By continent, Oceania exhibited the highest prevalence (15.7%; 2 studies, 414 birds), whereas Antarctica was the lowest (1.2%; 3 studies, 585 birds). By climate, the South Temperate Zone showed a relatively higher prevalence (14.2%; 27 studies, 13340 birds). By habitat, birds sampled in Forests and Woodlands and in Agro-pastoral landscapes had comparatively higher prevalences of 14.8% (84 studies, 125505 birds) and 13.9% (25 studies, 10170 birds), respectively (Additional file 4: Table S10).

Table S1. PRISMA 2020 checklist

| **Section and Topic** | **Item #** | **Checklist item** | **Location where item is reported** |
| --- | --- | --- | --- |
| **TITLE** | | |  |
| Title | 1 | Identify the report as a systematic review. | 1 |
| **ABSTRACT** | | |  |
| Abstract | 2 | See the PRISMA 2020 for Abstracts checklist. | N/A |
| **INTRODUCTION** | | |  |
| Rationale | 3 | Describe the rationale for the review in the context of existing knowledge. | N/A |
| Objectives | 4 | Provide an explicit statement of the objective(s) or question(s) the review addresses. | 4-5 |
| **METHODS** | | |  |
| Eligibility criteria | 5 | Specify the inclusion and exclusion criteria for the review and how studies were grouped for the syntheses. | 5, Table S3 |
| Information sources | 6 | Specify all databases, registers, websites, organisations, reference lists and other sources searched or consulted to identify studies. Specify the date when each source was last searched or consulted. | 5, Table S1 |
| Search strategy | 7 | Present the full search strategies for all databases, registers and websites, including any filters and limits used. | 5, Table S1 |
| Selection process | 8 | Specify the methods used to decide whether a study met the inclusion criteria of the review, including how many reviewers screened each record and each report retrieved, whether they worked independently, and if applicable, details of automation tools used in the process. | 6 |
| Data collection process | 9 | Specify the methods used to collect data from reports, including how many reviewers collected data from each report, whether they worked independently, any processes for obtaining or confirming data from study investigators, and if applicable, details of automation tools used in the process. | N/A |
| Data items | 10a | List and define all outcomes for which data were sought. Specify whether all results that were compatible with each outcome domain in each study were sought (e.g. for all measures, time points, analyses), and if not, the methods used to decide which results to collect. | 6 |
|  | 10b | List and define all other variables for which data were sought (e.g. participant and intervention characteristics, funding sources). Describe any assumptions made about any missing or unclear information. | Table S4 |
| Study risk of bias assessment | 11 | Specify the methods used to assess risk of bias in the included studies, including details of the tool(s) used, how many reviewers assessed each study and whether they worked independently, and if applicable, details of automation tools used in the process. | N/A |
| Effect measures | 12 | Specify for each outcome the effect measure(s) (e.g. risk ratio, mean difference) used in the synthesis or presentation of results. | 8 |
| Synthesis methods | 13a | Describe the processes used to decide which studies were eligible for each synthesis (e.g. tabulating the study intervention characteristics and comparing against the planned groups for each synthesis (item #5)). | 8 |
|  | 13b | Describe any methods required to prepare the data for presentation or synthesis, such as handling of missing summary statistics, or data conversions. | 8-9 |
|  | 13c | Describe any methods used to tabulate or visually display results of individual studies and syntheses. | 8-9 |
|  | 13d | Describe any methods used to synthesize results and provide a rationale for the choice(s). If meta-analysis was performed, describe the model(s), method(s) to identify the presence and extent of statistical heterogeneity, and software package(s) used. | 8-9 |
|  | 13e | Describe any methods used to explore possible causes of heterogeneity among study results (e.g. subgroup analysis, meta-regression). | 8-9, |
|  | 13f | Describe any sensitivity analyses conducted to assess robustness of the synthesized results. | 9 |
| Reporting bias assessment | 14 | Describe any methods used to assess risk of bias due to missing results in a synthesis (arising from reporting biases). | N/A |
| Certainty assessment | 15 | Describe any methods used to assess certainty (or confidence) in the body of evidence for an outcome. | 8 |
| **RESULTS** | | |  |
| Study selection | 16a | Describe the results of the search and selection process, from the number of records identified in the search to the number of studies included in the review, ideally using a flow diagram. | 9 |
|  | 16b | Cite studies that might appear to meet the inclusion criteria, but which were excluded, and explain why they were excluded. | N/A |
| Study characteristics | 17 | Cite each included study and present its characteristics. | 9 |
| Risk of bias in studies | 18 | Present assessments of risk of bias for each included study. | N/A |
| Results of individual studies | 19 | For all outcomes, present, for each study: (a) summary statistics for each group (where appropriate) and (b) an effect estimate and its precision (e.g. confidence/credible interval), ideally using structured tables or plots. | 12-13, Table S8-10 |
| Results of syntheses | 20a | For each synthesis, briefly summarise the characteristics and risk of bias among contributing studies. | N/A |
|  | 20b | Present results of all statistical syntheses conducted. If meta-analysis was done, present for each the summary estimate and its precision (e.g. confidence/credible interval) and measures of statistical heterogeneity. If comparing groups, describe the direction of the effect. | 12-13, Table S8-10 |
|  | 20c | Present results of all investigations of possible causes of heterogeneity among study results. | Table S10 |
|  | 20d | Present results of all sensitivity analyses conducted to assess the robustness of the synthesized results. | N/A |
| Reporting biases | 21 | Present assessments of risk of bias due to missing results (arising from reporting biases) for each synthesis assessed. | N/A |
| Certainty of evidence | 22 | Present assessments of certainty (or confidence) in the body of evidence for each outcome assessed. | 12-13, Table S8-10 |
| **DISCUSSION** | | |  |
| Discussion | 23a | Provide a general interpretation of the results in the context of other evidence. | 16-20 |
|  | 23b | Discuss any limitations of the evidence included in the review. | 19-20 |
|  | 23c | Discuss any limitations of the review processes used. | 19-20 |
|  | 23d | Discuss implications of the results for practice, policy, and future research. | 20 |
| **OTHER INFORMATION** | | |  |
| Registration and protocol | 24a | Provide registration information for the review, including register name and registration number, or state that the review was not registered. | N/A |
|  | 24b | Indicate where the review protocol can be accessed, or state that a protocol was not prepared. | N/A |
|  | 24c | Describe and explain any amendments to information provided at registration or in the protocol. | N/A |
| Support | 25 | Describe sources of financial or non-financial support for the review, and the role of the funders or sponsors in the review. | 21-22 |
| Competing interests | 26 | Declare any competing interests of review authors. | 22 |
| Availability of data, code and other materials | 27 | Report which of the following are publicly available and where they can be found: template data collection forms; data extracted from included studies; data used for all analyses; analytic code; any other materials used in the review. | 22 |

*From:*  Page MJ, McKenzie JE, Bossuyt PM, Boutron I, Hoffmann TC, Mulrow CD, et al. The PRISMA 2020 statement: an updated guideline for reporting systematic reviews. BMJ 2021;372:n71. doi: 10.1136/bmj.n71. This work is licensed under CC BY 4.0. To view a copy of this license, visit https://creativecommons.org/licenses/by/4.0/

Table S2: The detailed search strategy for each database

The database searches were conducted up to February 16, 2025.

| Database | Searches | Results |
| --- | --- | --- |
| PubMed | ("poultry"[MeSH Terms] OR "poultry"[All Fields] OR "poultries"[All Fields] OR "poultry s"[All Fields] OR "Fowl"[All Fields] OR ("birds"[MeSH Terms] OR "birds"[All Fields] OR "avian"[All Fields] OR "avians"[All Fields]) OR ("birds"[MeSH Terms] OR "birds"[All Fields] OR "aves"[All Fields]) OR ("birds"[MeSH Terms] OR "birds"[All Fields] OR "bird"[All Fields])) AND ("ticks"[MeSH Terms] OR "ticks"[All Fields] OR "tick"[All Fields]) | 2284 |
| Scopus | TITLE-ABS-KEY ( ( poultry ) OR( fowl ) OR ( avian ) OR ( aves ) OR ( bird ) ) AND TITLE-ABS-KEY ( *Amblyomma* OR *Archaeocroton* OR *Bothriocroton* OR *Haemaphysalis* OR *Hyalomma* OR *Nosomma* OR *Ixodes* OR *Dermacentor* OR *Rhipicentor* OR *Rhipicephalus* OR *Robertsicus* OR *Alveonasus* OR *Argas* OR *Navis* OR *Ogadenus* OR *Proknekalia* OR *Secretargas* OR *Alectorobius* OR *Antricola* OR *Carios* OR *Chiropterargas* OR *Nothoaspis* OR *Ornithodoros* OR *Otobius* OR *Reticulinasus* OR *Subparmatus* OR *Nuttalliella* OR tick ) | 2952 |
| Web of Science Core Collection | (ALL=(Poultry) OR ALL=(Fowl) OR ALL=(Avian) OR ALL=(Aves) OR ALL=(Bird)) AND ALL=(tick) | 2321 |
| CNKI | 检索范围：总库（篇关摘：蜱(模糊)）AND（篇关摘：鸟 + 禽(模糊)） | 233 |
| GenBank | Search (bird or avian or fowl or poultry) AND (tick or *Amblyomma* or *Haemaphysalis* or *Hyalomma* or *Ixodes* or *Dermacentor* or *Rhipicephalus* or *Argas* or *Alectorobius* or *Alveonasus* or *Ornithodoros*) Filters: Protists; Bacteria; Viruses. | 1125 |

Table S3: The inclusion and exclusion criteria of screening publications

| **Criteria** | **Guidance** | **Outcome** |
| --- | --- | --- |
| **Title/Abstract screening** | | |
| #1: Birds | Does the Title/Abstract refer to birds or their nests that were collected in the field? | If Yes, remain and evaluate #2.  If No, exclude. |
| #2: Ticks | Does the Title/Abstract refer to ticks associated with birds? | If Yes, remain and evaluate #3.  If No, exclude. |
| #3: Tick associated pathogens | Does the Title/Abstract refer to tick associated pathogens detected from ticks or birds？ | Whether Yes or No, evaluate #4. |
| #4: Not review | Does the Title/Abstract refer to the article that is Not a review? (Not reviewing the published articles, with presenting new primary data) | If Yes, remain and evaluate #5.  If No, exclude. |
| #5: Articles | Does the Title/Abstract refer to the article that is not a conference proceeding or a book? | If No, remain for full text review.  If Yes. exclude. |
| **Full text screening** | | |
| #1: Full text | Does the full text of the article exist? | If Yes, remain and evaluate #2.  If No, exclude. |
| #2: Re-screening | Does the article meet the screening criteria before?  1-not veterinary clinical research/ review  2-not theoretical research on model construction or molecular mechanisms  3-samples comprising both avian hosts and ticks (for ticks)  4-infection in natural environment (for pathogens) | If Yes, remain and evaluate #3.  If No, exclude. |
|  | 5-pathogen detection must be accompanied by a specified method (for pathogens) |  |
| #3: Species identification | Does the article report specific species identification results?  1-birds were identified to at least the order level  2-ticks were identified to at least the genus level | If Yes, remain and evaluate #4.  If No, exclude. |
| #4: Geographical information | Does the article refer the geographical information?   1. geographic location information at country or subnational administrative divisions levels 2. exact locations or only marked the latitude and longitude | If Yes, remain for data extracting.  If No, exclude. |

Table S4: List of variables extracted from reviewed studies

| Variables | Explanation |
| --- | --- |
| Basic information of the study |  |
| Reference ID | Unique identifier assigned to an article. |
| Article title | Article title that included in the review. |
| Authors | Authors of the included article. |
| Publication year | Publication year of the included article. |
| Study period | The start and end time for the period over which the sample collected. |
| Study site | Including the country, specific locality (province, city, or sampling point), and precise geographic coordinates (latitude and longitude). |
| Habitat type | Describes the ecological environment of the sampling site, including both natural and human-modified habitats. |
| Ticks associated with birds |  |
| Species of ticks | Including taxonomic information at the family, genus, and species levels. |
| Species of birds | Including taxonomic classification at the order, family, genus, and species levels, as well as common names. |
| Number of birds collected | Refers to the total number of birds captured or examined in the field, described at the order level. |
| Number of birds infested | Refers to the number of birds that were found to be infested with ticks among all examined individuals, described at the order level. |
| Bird–tick association | Describes the relationship between the collected ticks and birds, including (1) ticks directly removed from bird bodies, (2) ticks collected from bird nests or roosting sites, and (3) ticks associated with prey items consumed by birds. |
| Non-native ticks | Refers to tick species that are absent or only rarely reported in the study area. |
| Pathogen detected from ticks/birds |  |
| Species of pathogen | Refers to the identified pathogen detected in ticks or birds, including its biological type (bacterium, virus, or protozoan) and full taxonomic classification at the family, genus, and species levels. Tick symbionts were excluded from the study. |
| Source of detection | Refers to the biological material from which the pathogen was detected, including various bird tissues (e.g., blood, liver, spleen, or cloacal swabs) or ticks. |
| Detection method | The technology used to detect tick-borne pathogens. |

Table S5. The classification definition of the seven ecological groups of wild birds

| **Ecological groups** | **Ecological and morphological characteristics** | **Bird orders** | **Representative species** | **References** |
| --- | --- | --- | --- | --- |
| Songbirds | Songbirds are small- to medium-sized, agile perching birds in the order Passeriformes. They are mainly diurnal, forage on insects, seeds and fruits from canopy to ground, and have slender legs with an anisodactyl foot (three toes forward, one long hind toe) adapted for perching and nest building. | Passeriformes | *Corvus frugilegus,*  *Turdus merula* | 1, 2 |
| Climbing birds | Climbing birds are small- to medium-sized arboreal species that move mainly on trunks and branches in forests, hills or cliffs, feeding on bark-dwelling insects and fruits. They have short, strong zygodactyl feet that provide a powerful grasp but limited flight. | Bucerotiformes, Cuculiformes, Coliiformes, Coraciiformes, Caprimulgiformes, Psittaciformes, Piciformes, Trogoniformes,  Musophagiformes | *Dendrocopos major,*  *Myiopsitta monachus,*  *Crotophaga ani* | 2, 3 |
| Landfowl | Landfowl are predominantly terrestrial birds that engage primarily in ground-based activities such as foraging and nest building. They are primarily herbivorous, feeding on seeds, stems, and fruits, and forage almost exclusively on the ground. Their morphology reflects this lifestyle, featuring robust bodies, curved beaks, and powerful hind limbs well-suited for ground locomotion and foraging, while being generally less adapted for sustained, long-distance flight. | Apterygiformes, Columbiformes, Casuariiformes, Cariamiformes, Galliformes, Otidiformes,  Pteroclidiformes,  Struthioniformes, Tinamiformes | *Gallus gallus,*  *Numida meleagris,*  *Phasianus colchicus* | 4, 5 |
| Waterfowl | Waterfowl are birds adapted to aquatic habitats, feeding on aquatic plants and small animals while foraging on or just below the water surface. They have legs with fully webbed feet, broad bills and dense plumage, better for swimming than walking. | Anseriformes, Procellariiformes,  Phaethontiformes, Sphenisciformes, Suliformes | *Anas zonorhyncha,*  *Anas platyrhynchos* | 2, 6 |
| Shorebirds | Shorebirds (waders) are small- to medium-sized, long-legged birds that forage in shallow waters along coasts and wetlands, feeding on invertebrates and small aquatic prey. They are strong fliers, with limited foot webbing and a characteristic long bill, neck and long legs. | Charadriiformes, Ciconiiformes, Eurypygiformes, Gruiformes, Phoenicopteriformes, Pelecaniformes | *Larus argentatus,*  *Sterna hirundo,*  *Fratercula arctica* | 3, 7 |
| Raptors | Raptors are medium- to large-sized carnivorous birds of prey, mostly diurnal, that hunt or scavenge vertebrates. They have powerful hooked beaks, strong feet with sharp talons, and exceptionally keen eyesight, all specialized for detecting, seizing and tearing prey. | Accipitriformes, Falconiformes, Strigiformes | *Gyps fulvus,*  *Bubo virginianus,*  *Milvago chimachima* | 8, 9 |
| Aerial birds | Aerial birds spend most of their lives in flight, feeding on airborne insects or nectar and often migrating long distances. They have long, pointed wings, lightweight bodies and small, weak feet poorly suited to walking but excellent for sustained high-speed flight. | Apodiformes | *Apus apus,*  *Archilochus colubris, Aerodramus fuciphagus,*  *Aerodramus fuciphagus* | 10, 11 |

**Reference**

1. Moyle RG, Oliveros CH, Andersen MJ, et al. Tectonic collision and uplift of Wallacea triggered the global songbird radiation. Nat Commun. 2016;7:12709. Published 2016 Aug 30.
2. Jetz W, Thomas GH, Joy JB, Hartmann K, Mooers AO. The global diversity of birds in space and time. Nature. 2012;491:444-448.
3. Prum RO, Berv JS, Dornburg A, et al. A comprehensive phylogeny of birds (Aves) using targeted next-generation DNA sequencing. Nature. 2015;526:569-573.
4. Hosner PA, Faircloth BC, Glenn TC, Braun EL, Kimball RT. Avoiding Missing Data Biases in Phylogenomic Inference: An Empirical Study in the Landfowl (Aves: Galliformes). Mol Biol Evol. 2016;33:1110-1125.
5. Kimball RT, Hosner PA, Braun EL. A phylogenomic supermatrix of Galliformes (Landfowl) reveals biased branch lengths. Mol Phylogenet Evol. 2021;158:107091.
6. Roberts A, Scarpignato AL, Huysman A, Hostetler JA, Cohen EB. Migratory connectivity of North American waterfowl across administrative flyways. Ecol Appl. 2023;33:e2788.
7. Ericson PG, Envall I, Irestedt M, Norman JA. Inter-familial relationships of the shorebirds (Aves: Charadriiformes) based on nuclear DNA sequence data. BMC Evol Biol. 2003;3:16.
8. McClure CJW, Lepage D, Dunn L, et al. Towards reconciliation of the four world bird lists: hotspots of disagreement in taxonomy of raptors. Proc Biol Sci. 2020;287:20200683.
9. Buechley ER, Santangeli A, Girardello M, Neate-Clegg MHC, Oleyar D, McClure CJW, et al. Global raptor research and conservation priorities: Tropical raptors fall prey to knowledge gaps. Diversity and Distributions. 2019;25:856–869.
10. Åkesson S, Klaassen R, Holmgren J, Fox JW, Hedenström A. Migration routes and strategies in a highly aerial migrant, the common swift Apus apus, revealed by light-level geolocators. PLoS One. 2012;7:e41195.
11. Ingersoll R, Haizmann L, Lentink D. Biomechanics of hover performance in Neotropical hummingbirds versus bats. Sci Adv. 2018;4:eaat2980.
