# Supplementary material for: From flyways to foci: a systematic review and meta-analysis on the role of birds in the maintenance and global dispersal of ticks and tick-borne pathogens
Source: Parasit Vectors. 2026 Jan 24;19:88. doi: 10.1186/s13071-025-07238-4 (PMC12914891; doi:10.1186/s13071-025-07238-4)

Figure S1: Global distribution of birds associated with ticks

Hollow symbols indicate records with confirmed sampling coordinates, whereas solid symbols represent polygon-based records.

**(Ⅰ) Distribution of the Group Songbirds**


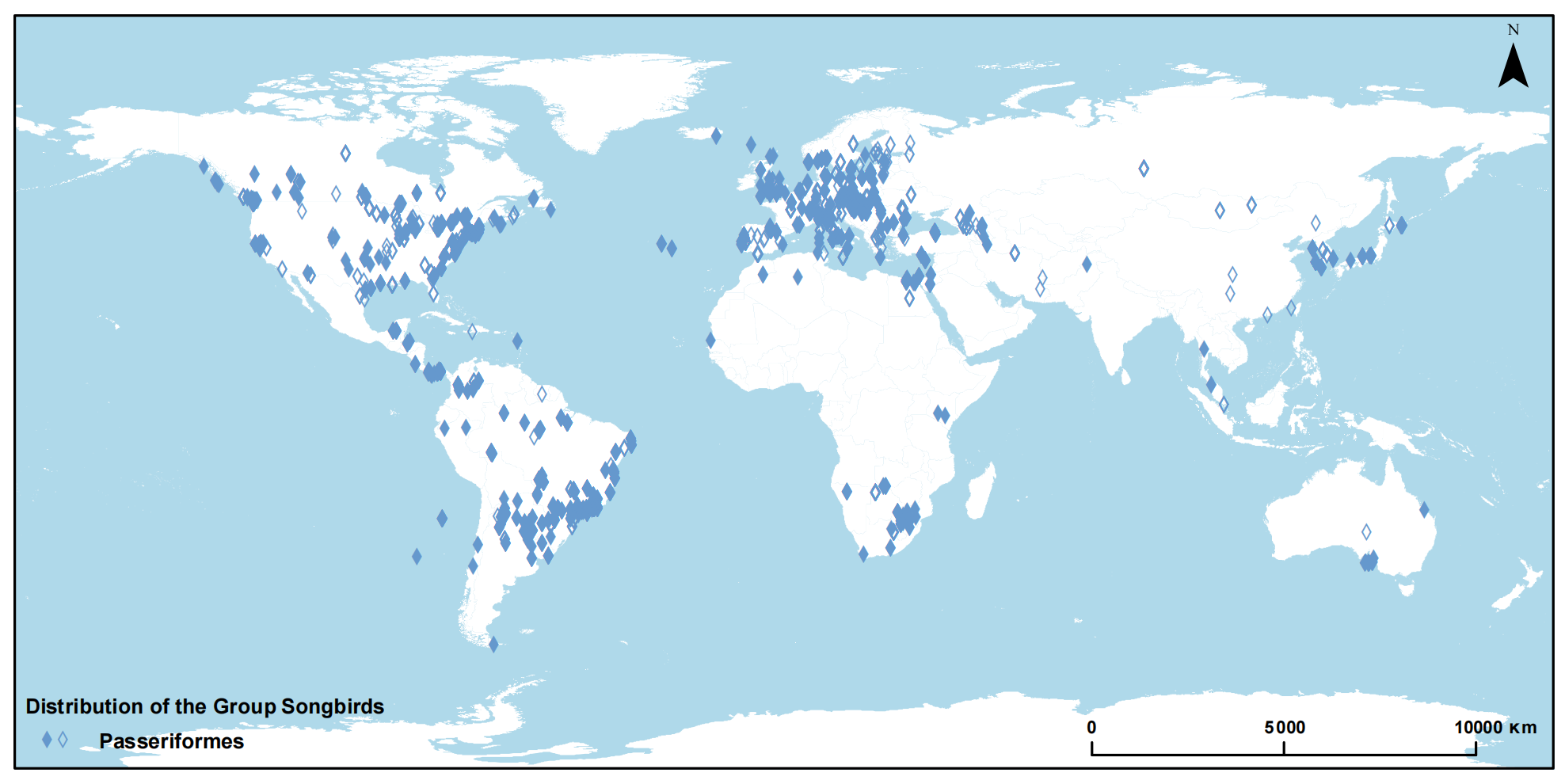


**(Ⅱ) Distribution of the Group Landfowl**


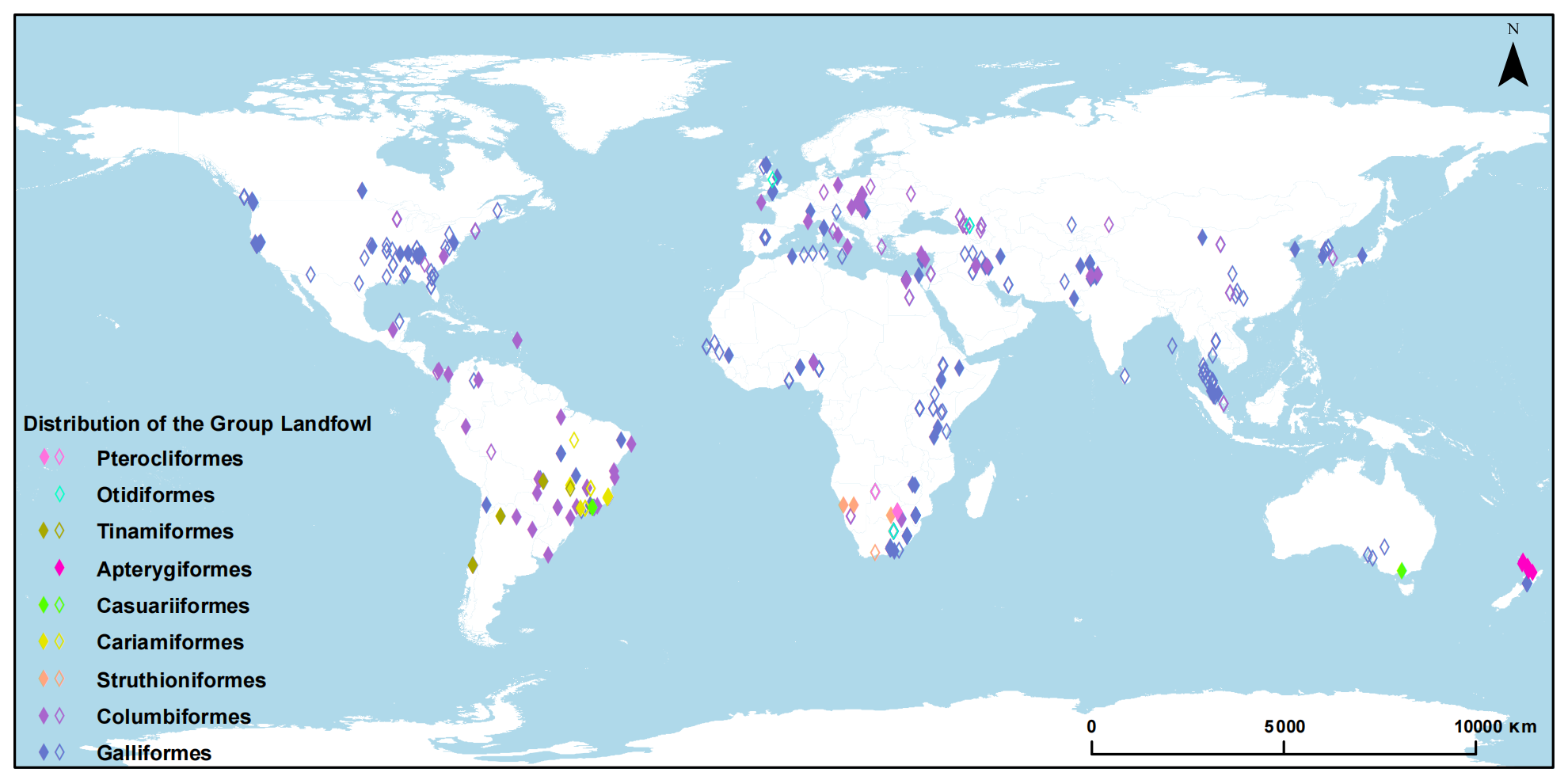


**(Ⅲ) Distribution of the Group S****horebirds**


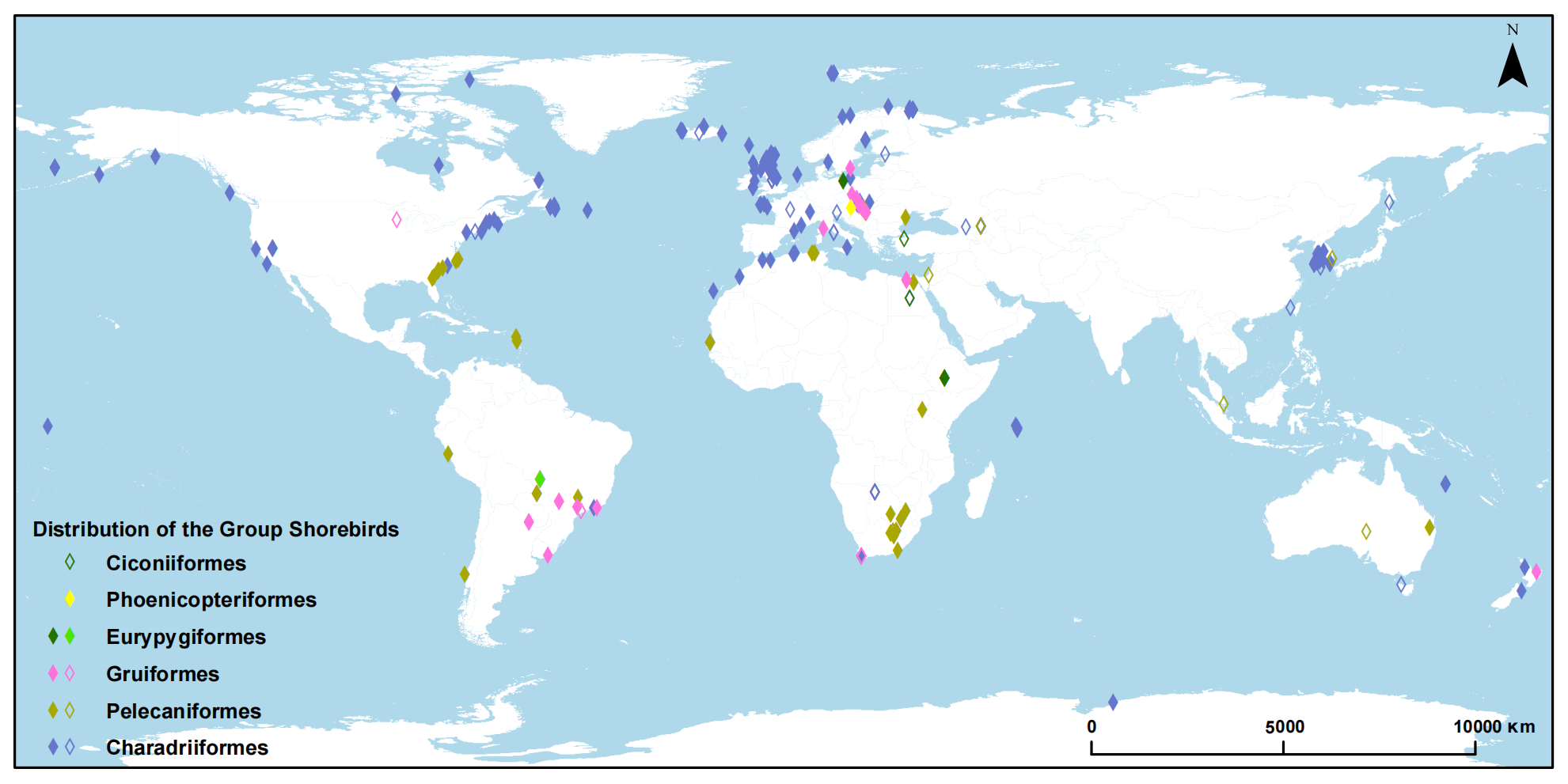


**(Ⅳ) Distribution of the Group Raptors**


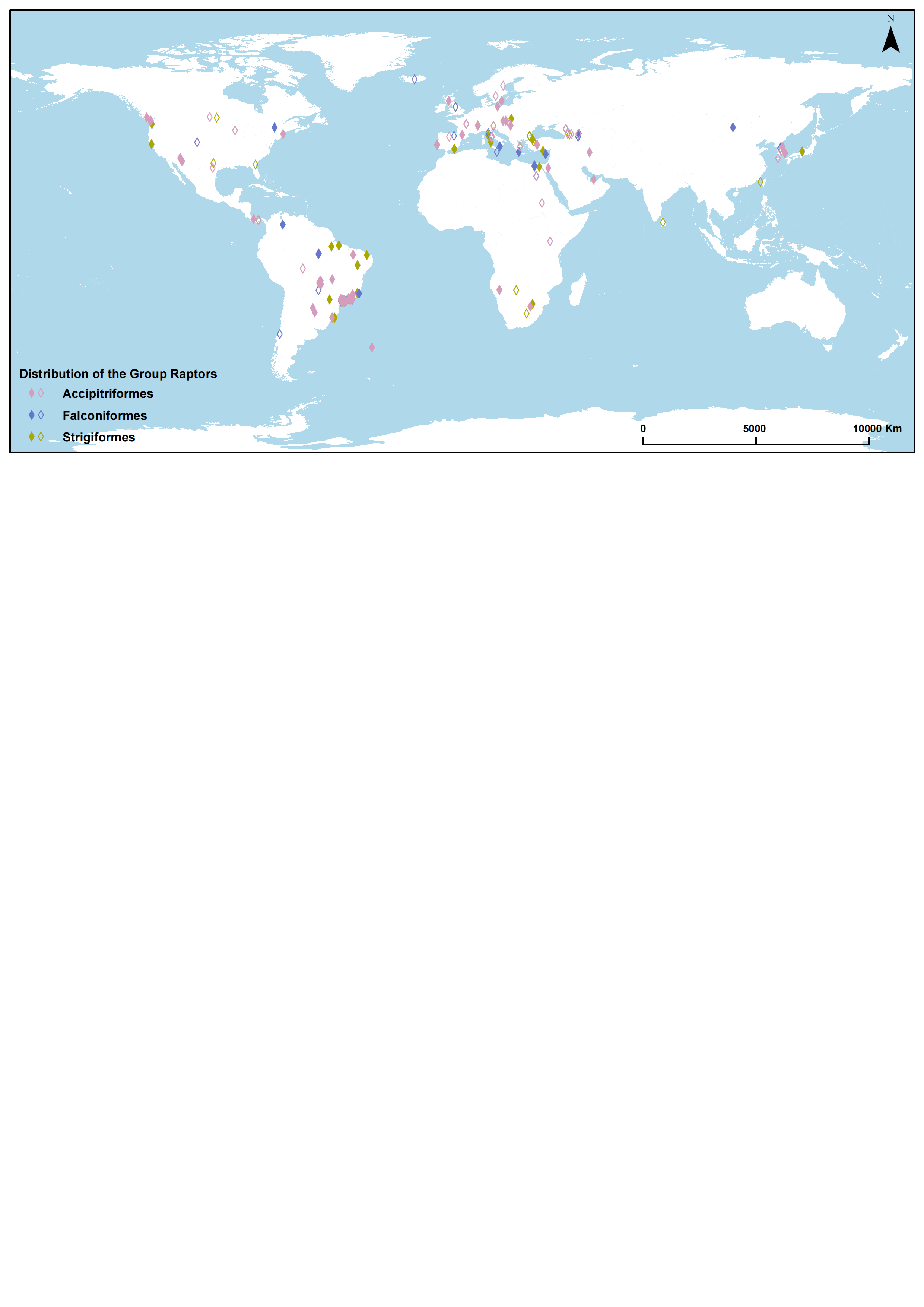


**(Ⅴ) Distribution of the Group Climbing birds**


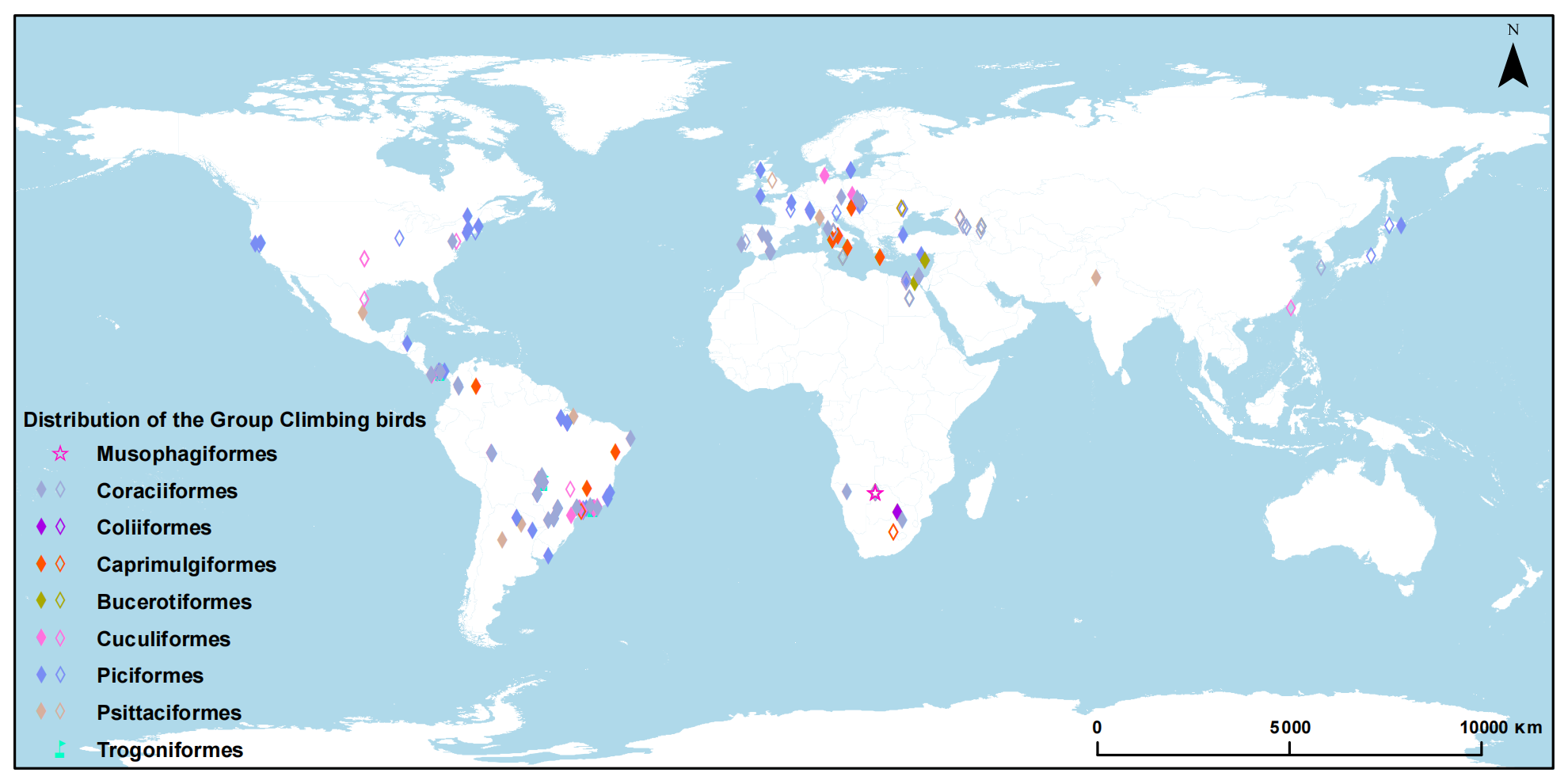


**(Ⅵ) Distribution of the Group Waterfowl**


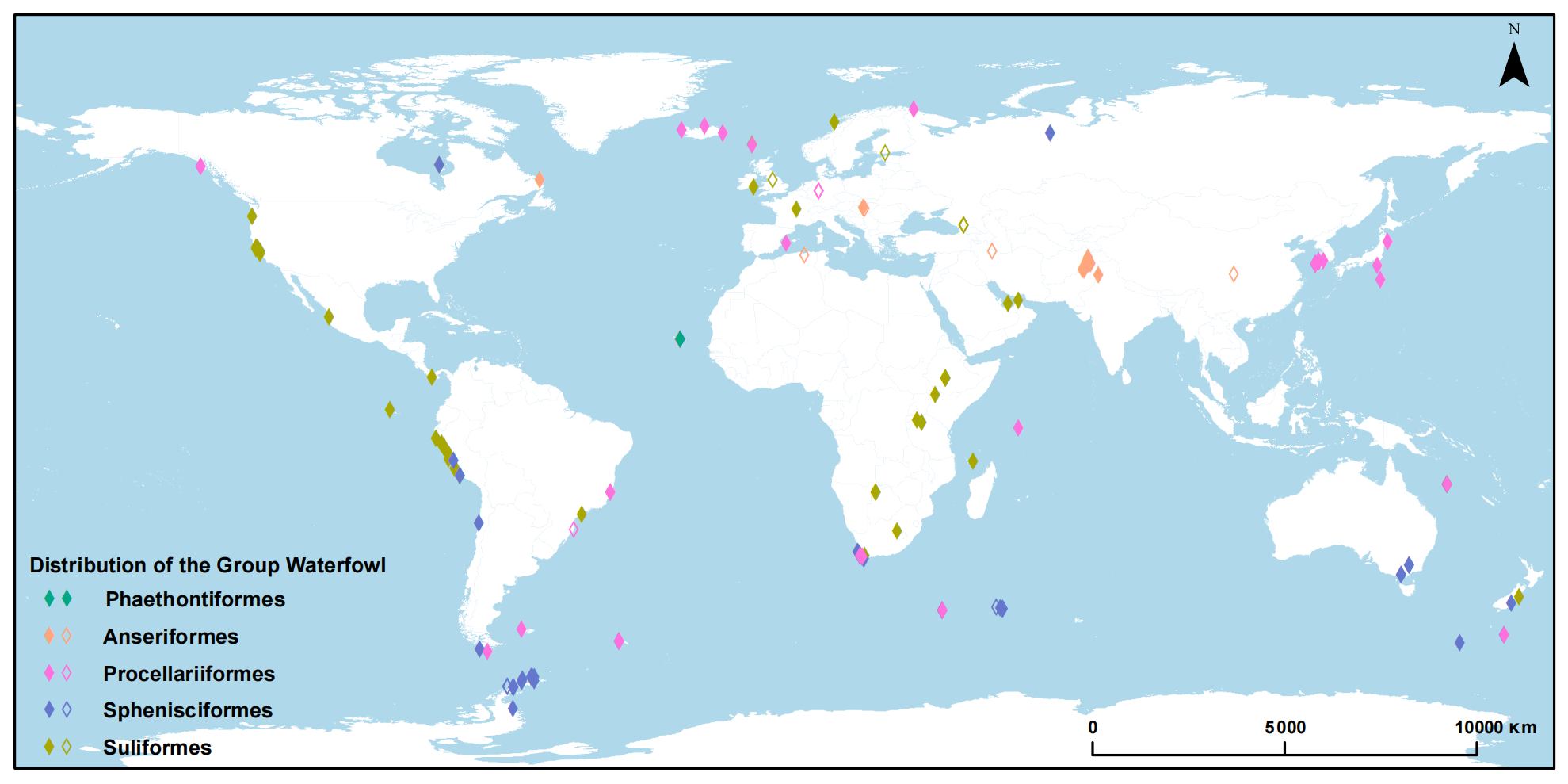


**(Ⅶ) Distribution of the order Aerial Birds**


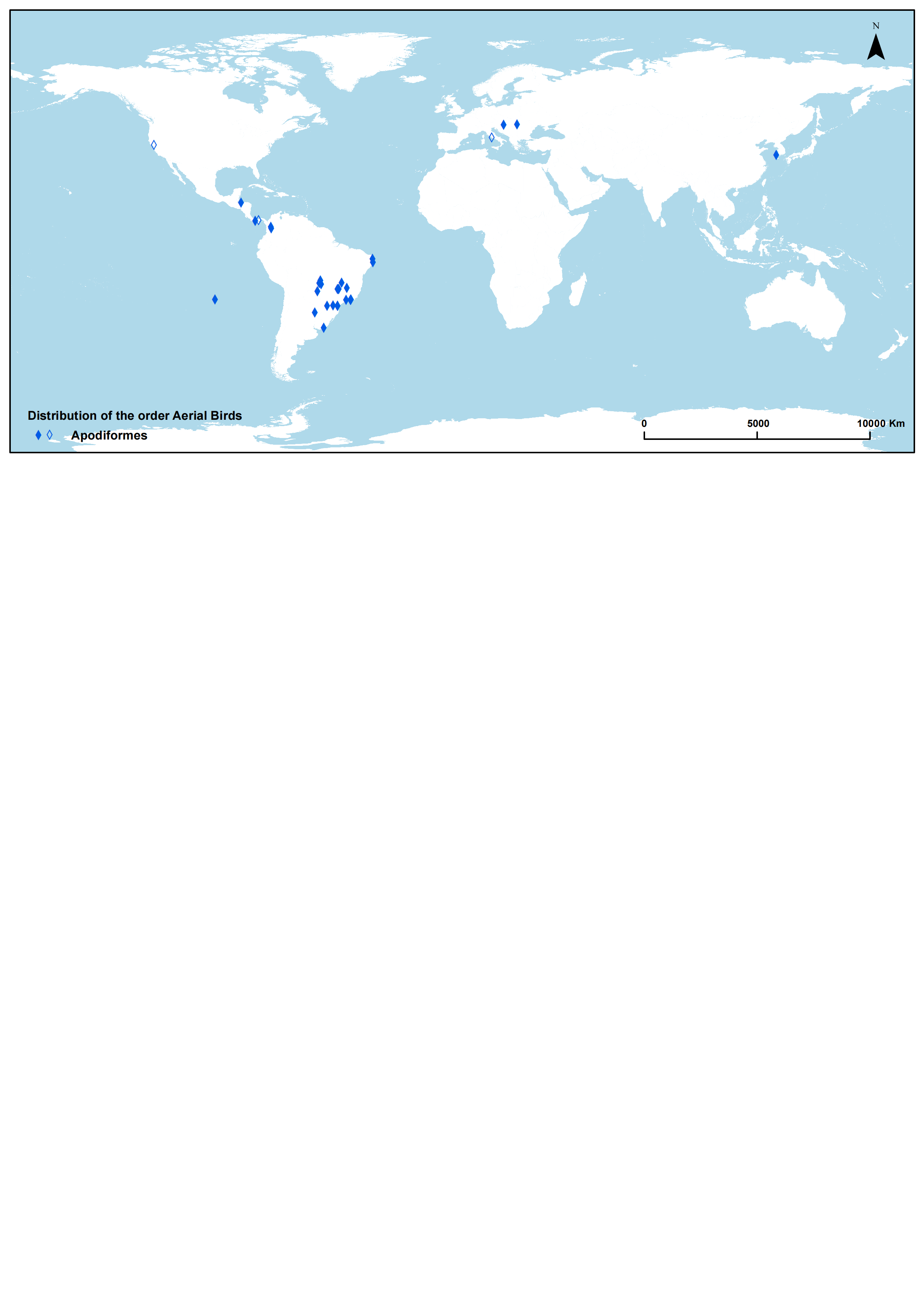


Figure S2: Continental distribution and composition of bird groups

Figures indicate the number of reported geocoordinates (after deduplication).

**(Ⅰ) Bird composition across continents**


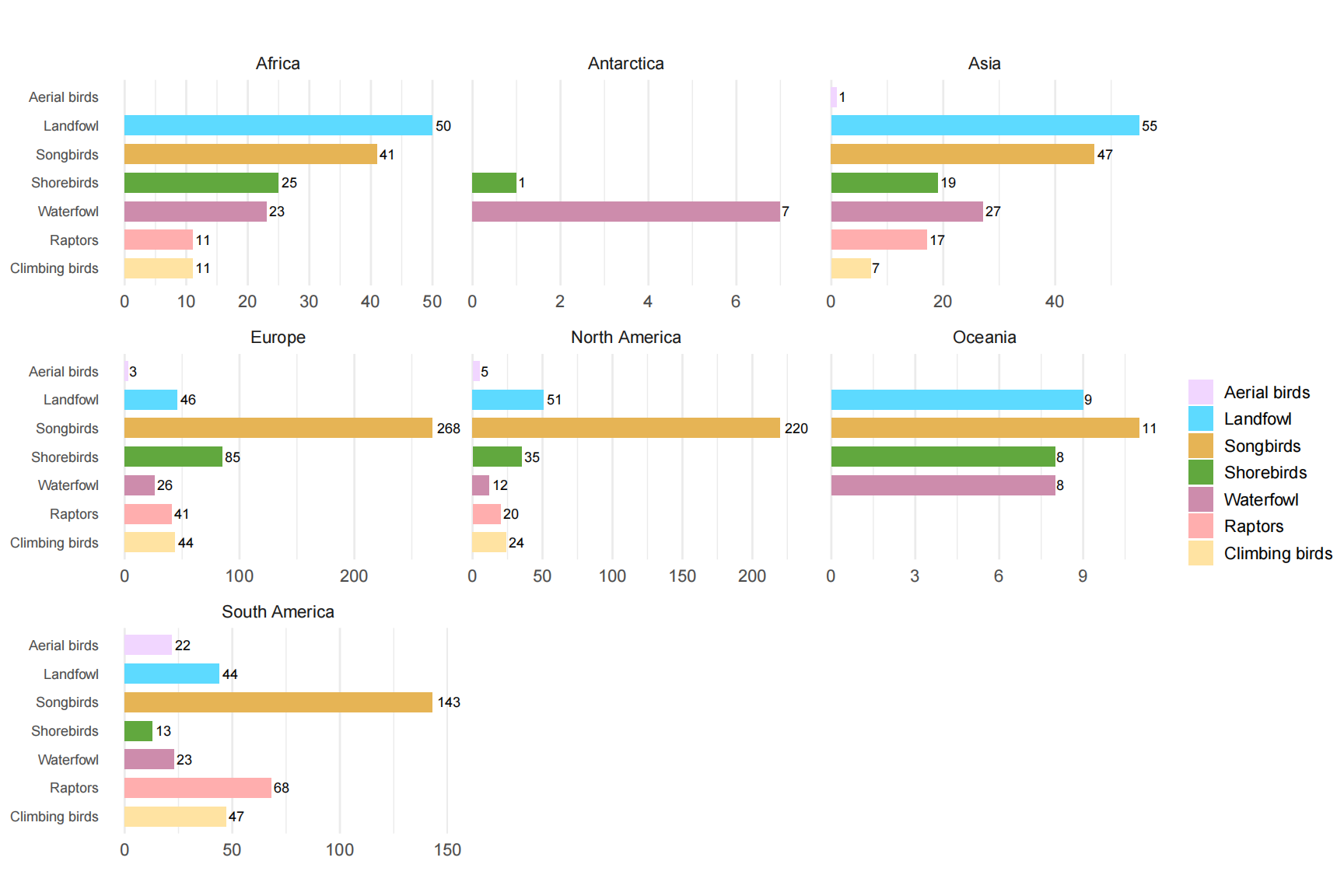


**(Ⅱ)** **Continental distribution of bird groups**


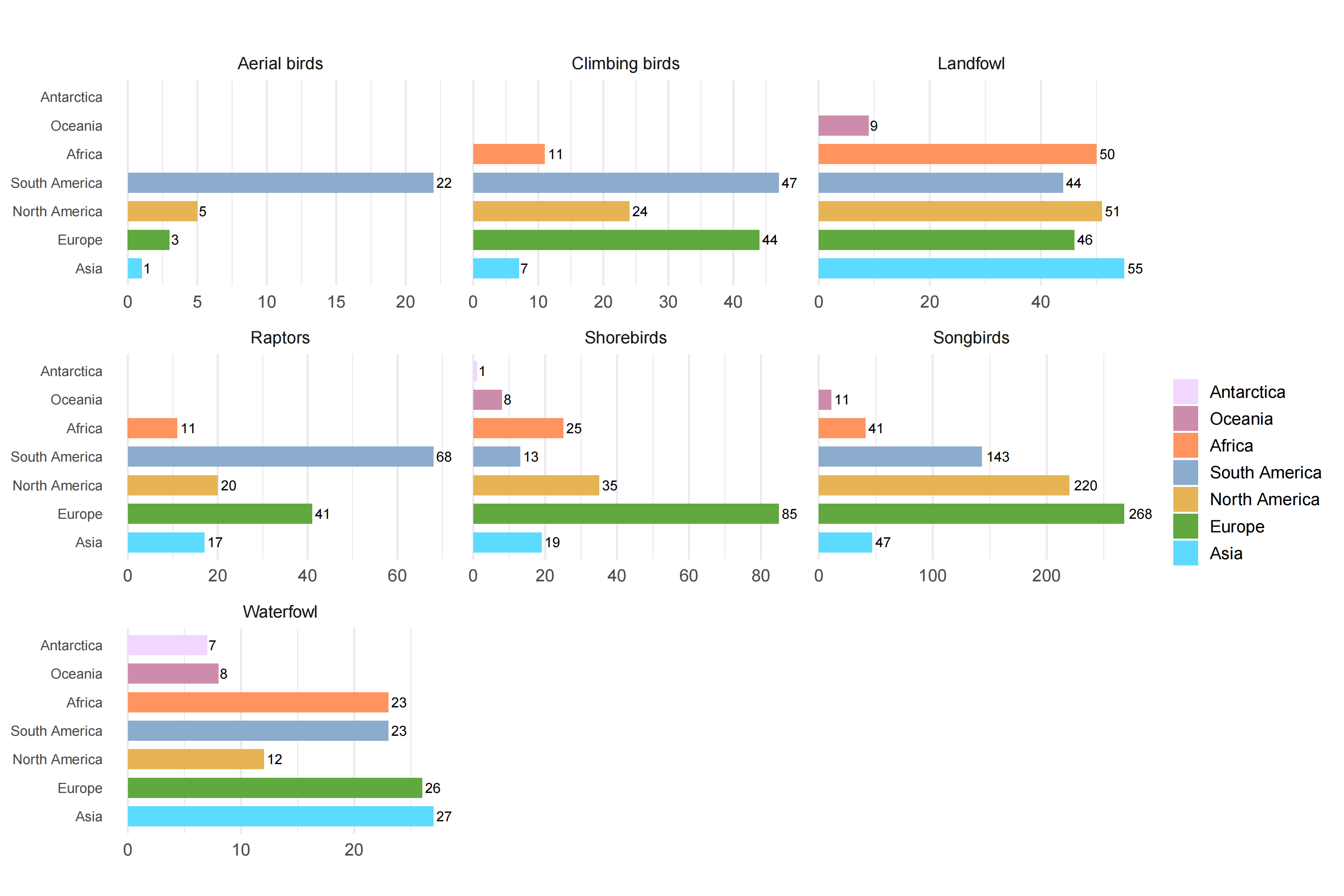


Figure S3: Global distribution of ornithophilic ticks

Hollow symbols indicate records with confirmed sampling coordinates, whereas solid symbols represent polygon-based records.


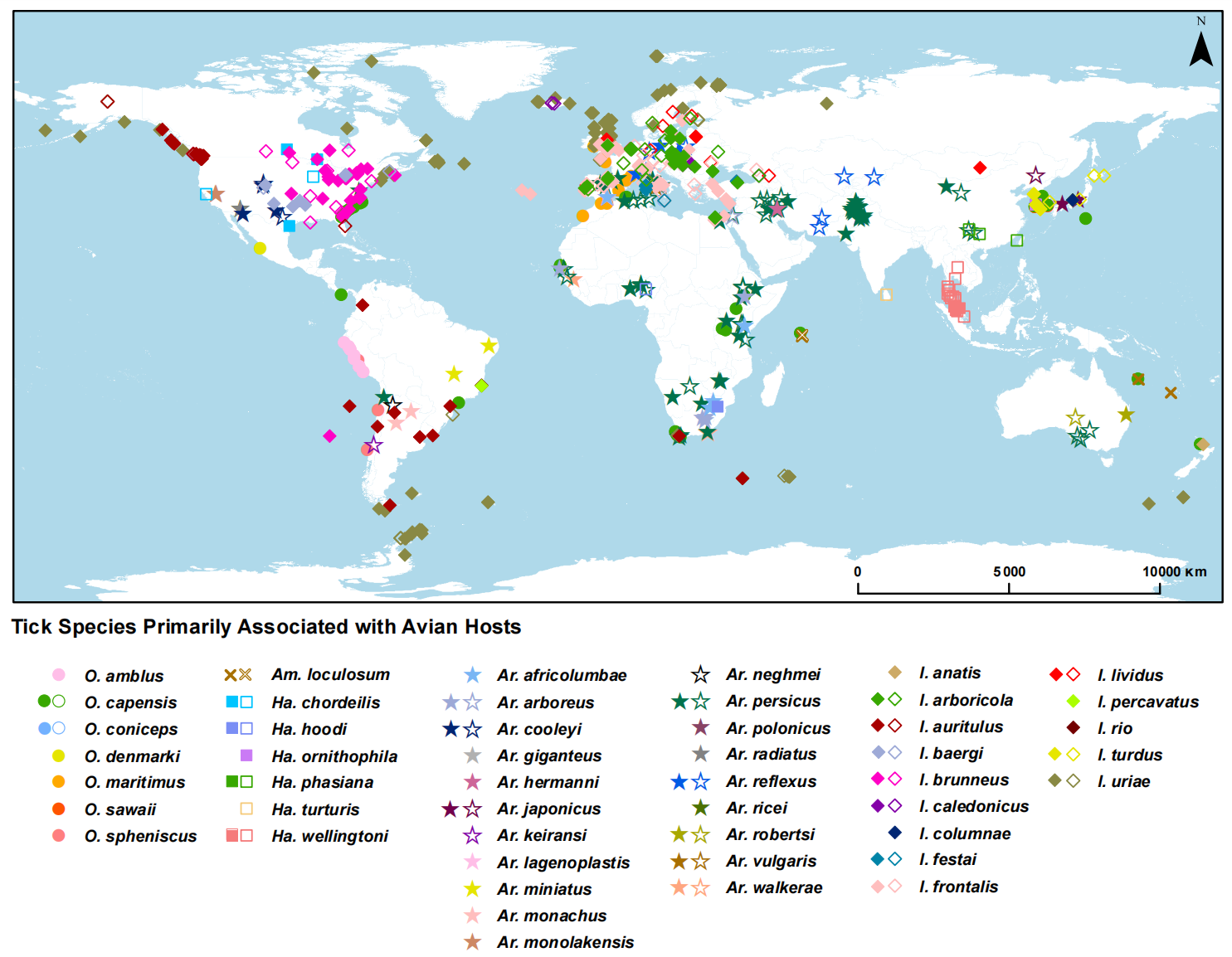


Figure S4: Global distribution of BATs

Ticks collected exclusively from birds or bird nests. Hollow symbols indicate records with confirmed sampling coordinates, whereas solid symbols represent polygon-based records.

**(Ⅰ) Distribution of *Ixodes***


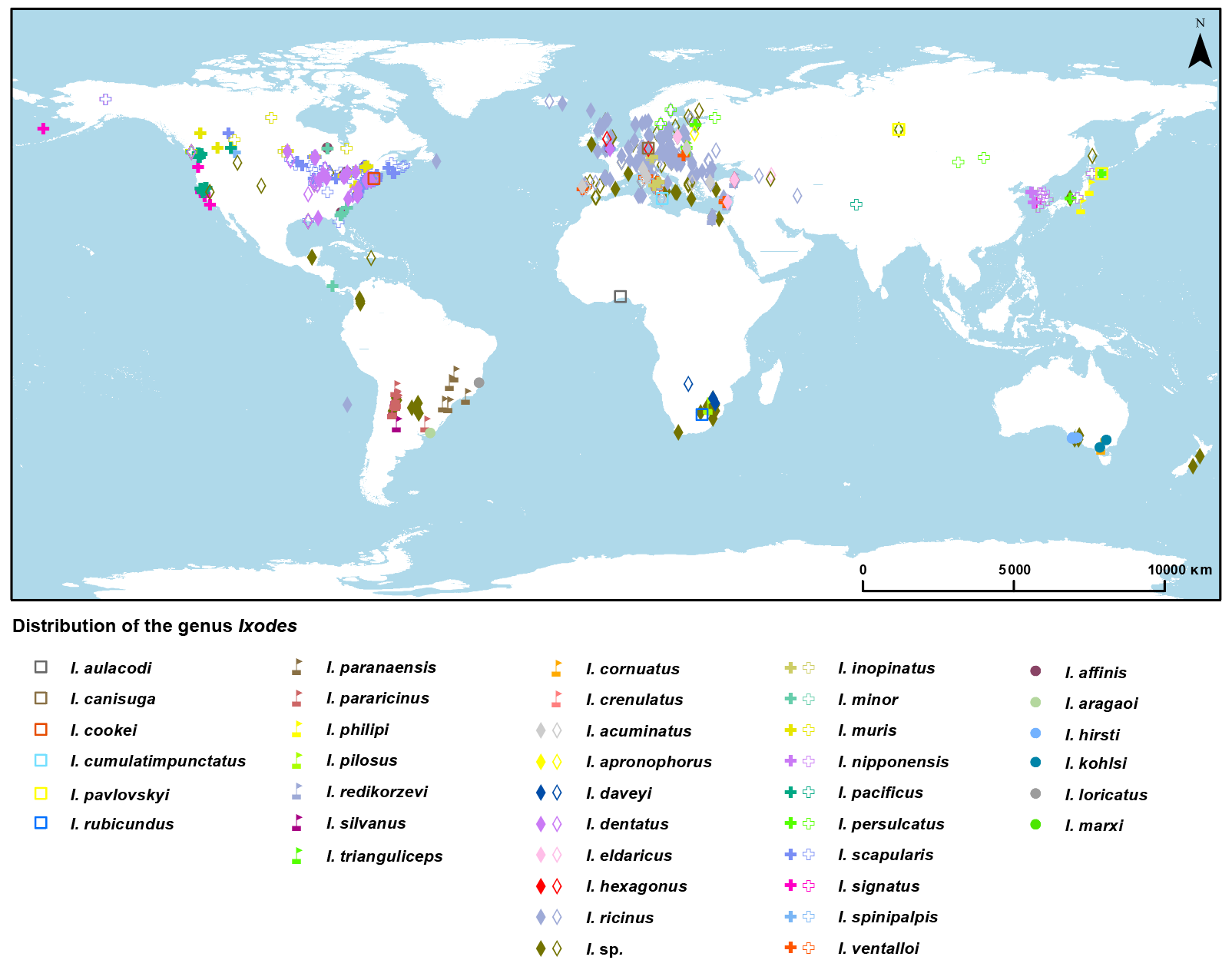


**(Ⅱ) Distribution of *Amblyomma***


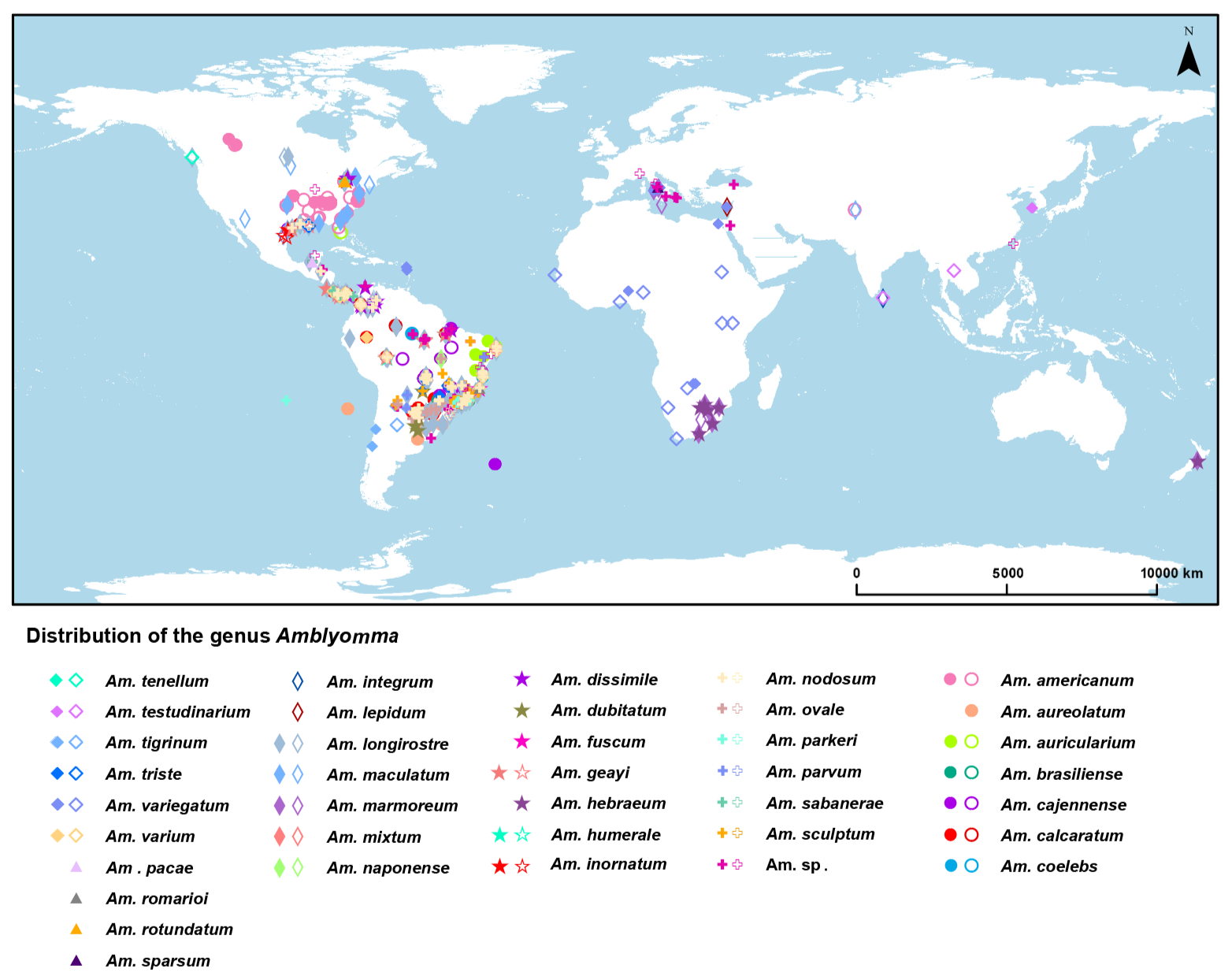


**(Ⅲ) Distribution of *Haemaphysalis***

**
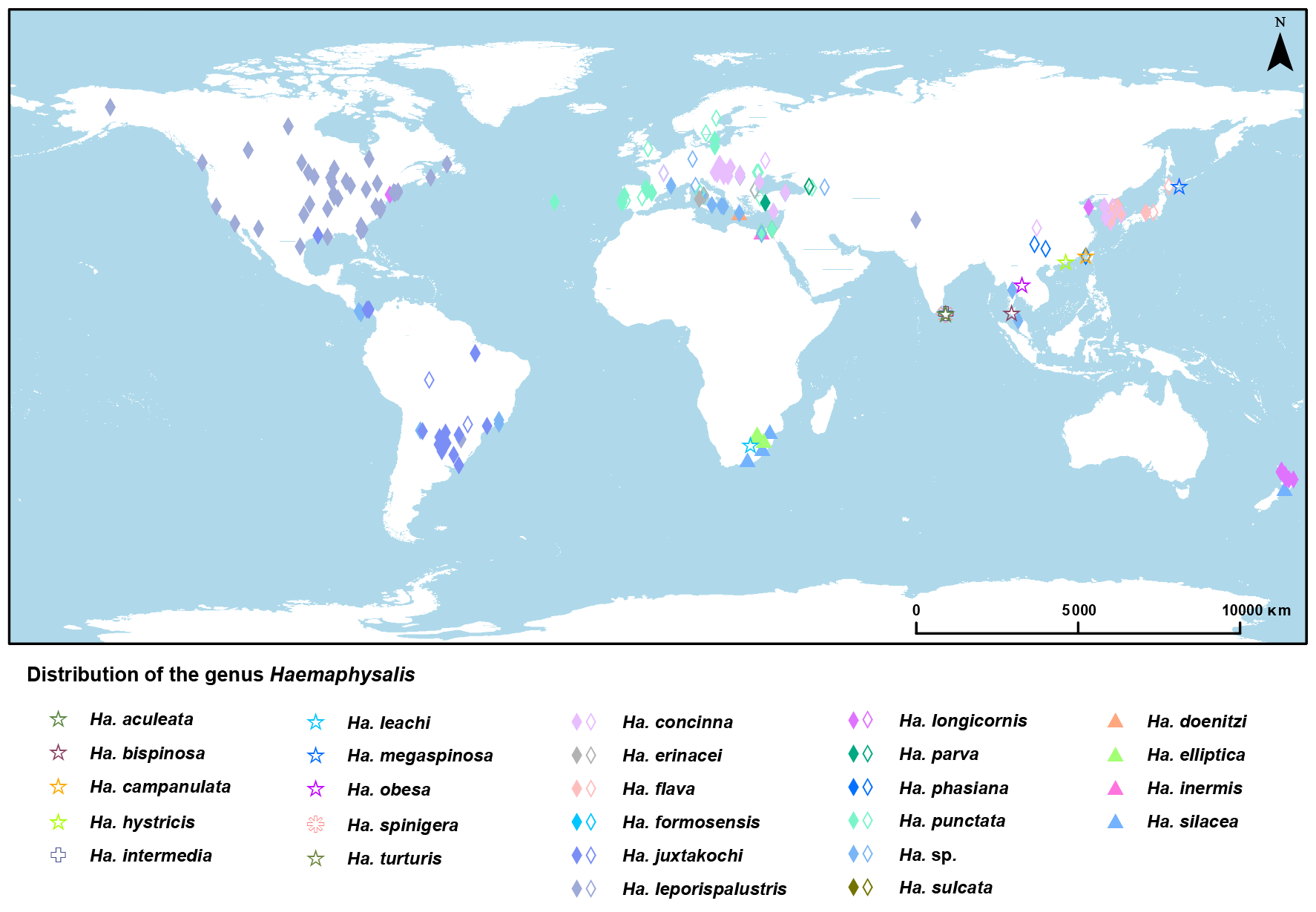
**

**(Ⅳ) Distribution of *Rhipicephalus***


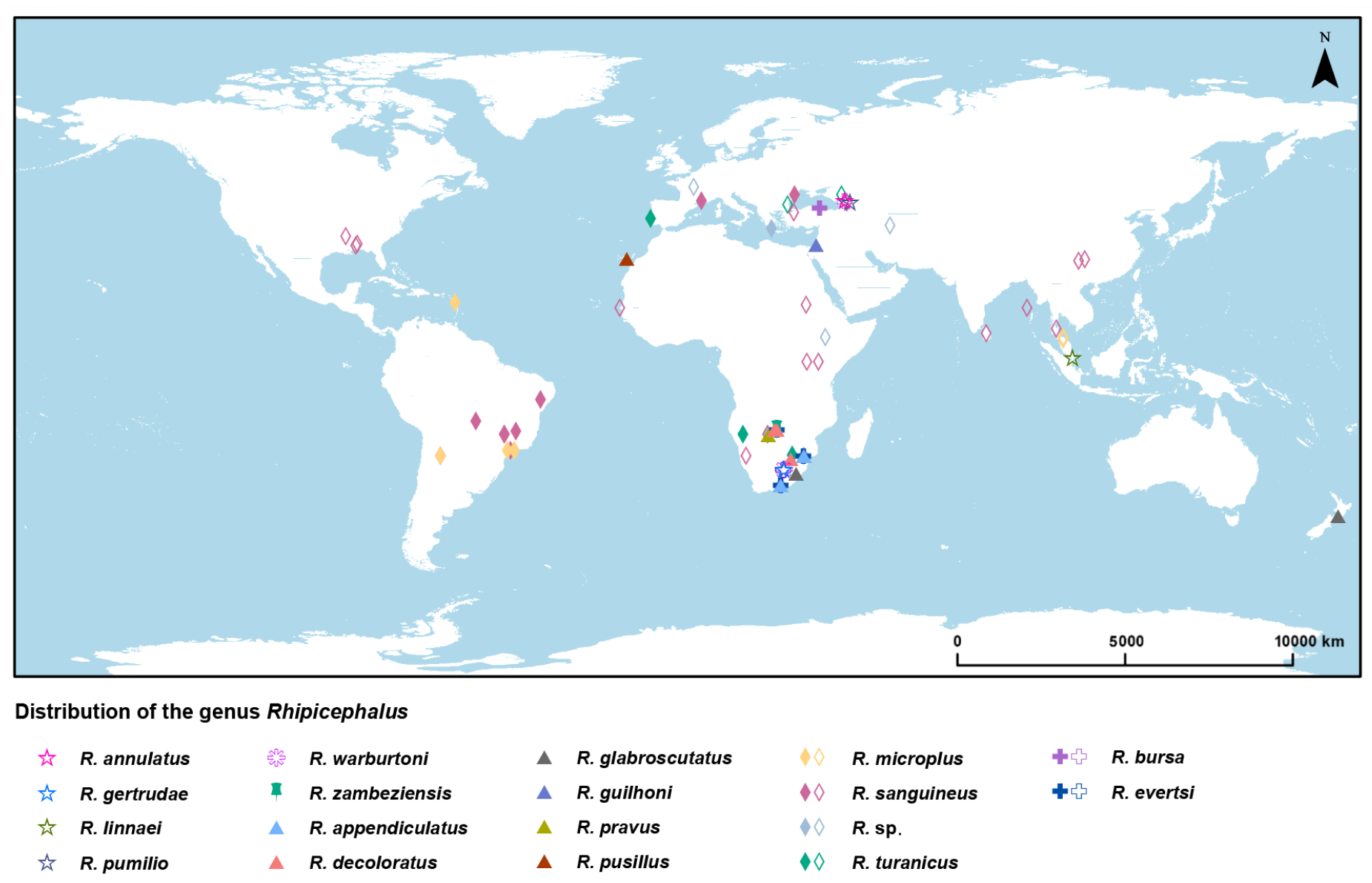


**(Ⅴ) Distribution of *Hyalomma***


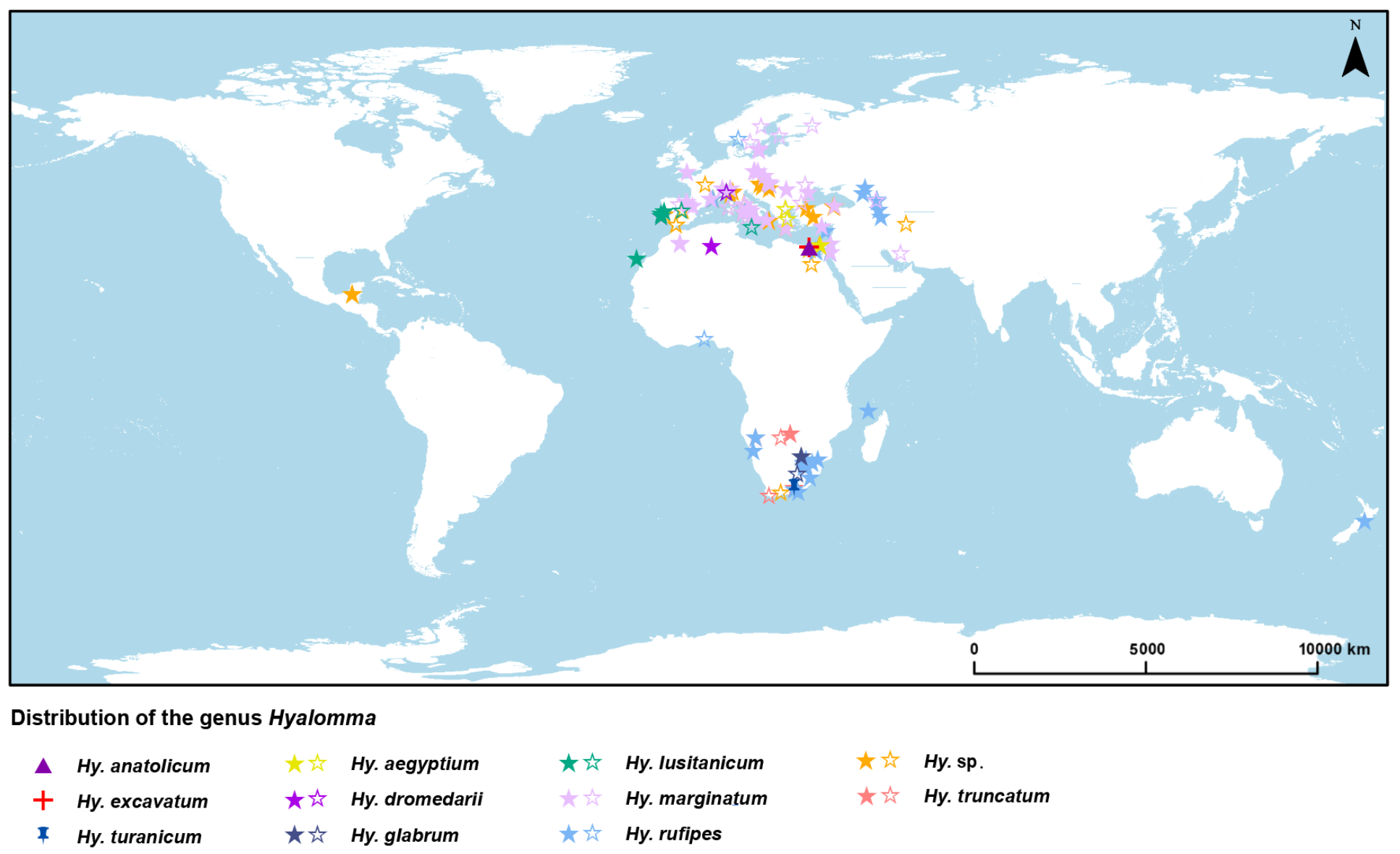


**(Ⅵ) Distribution of Other Tick Genera**


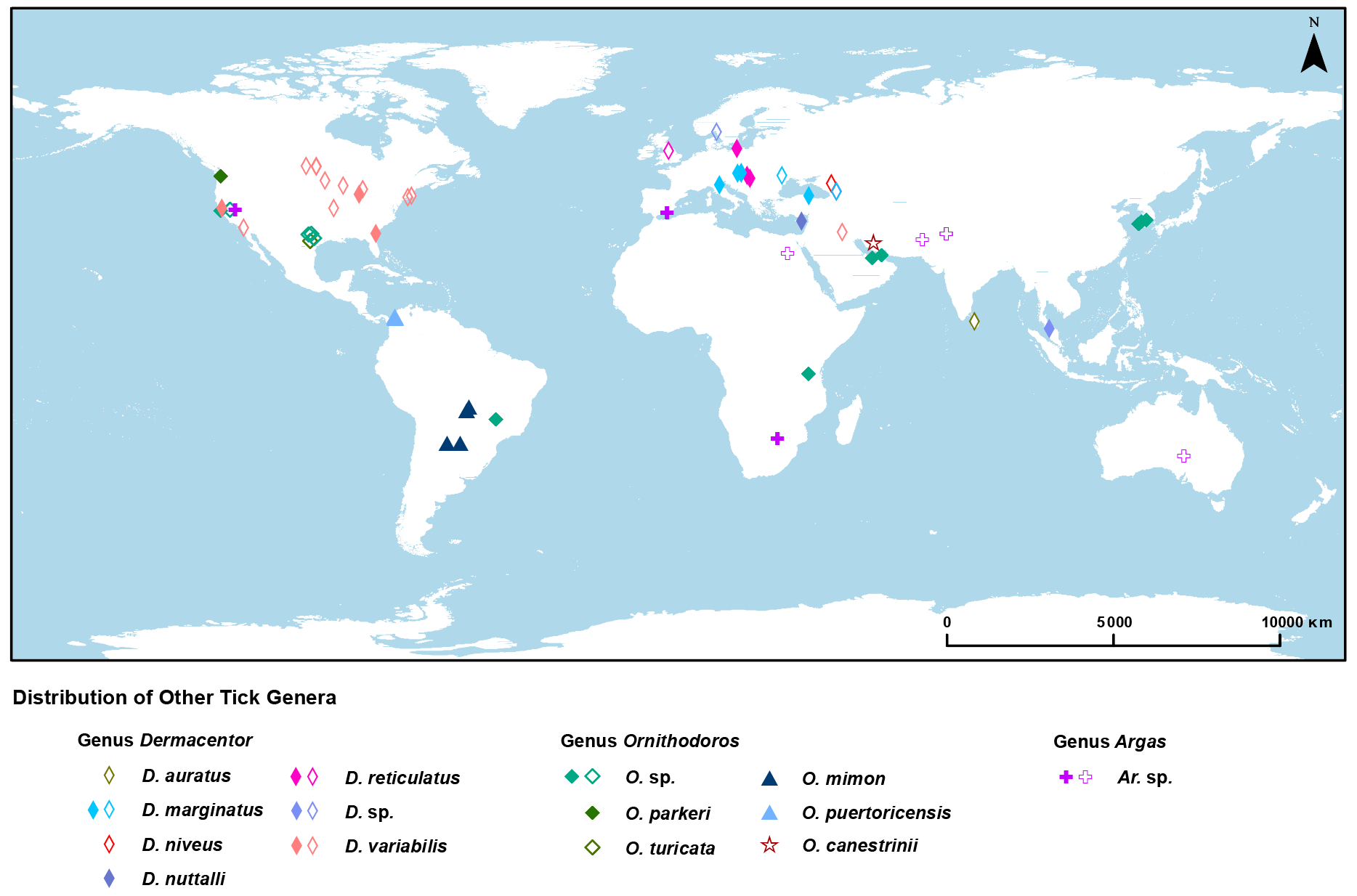


Figure S5: Continental distribution and composition of BATs

Figures indicate the number of reported geocoordinates (after deduplication).

**(Ⅰ) Genus Gomposition of Ticks Across Continents**


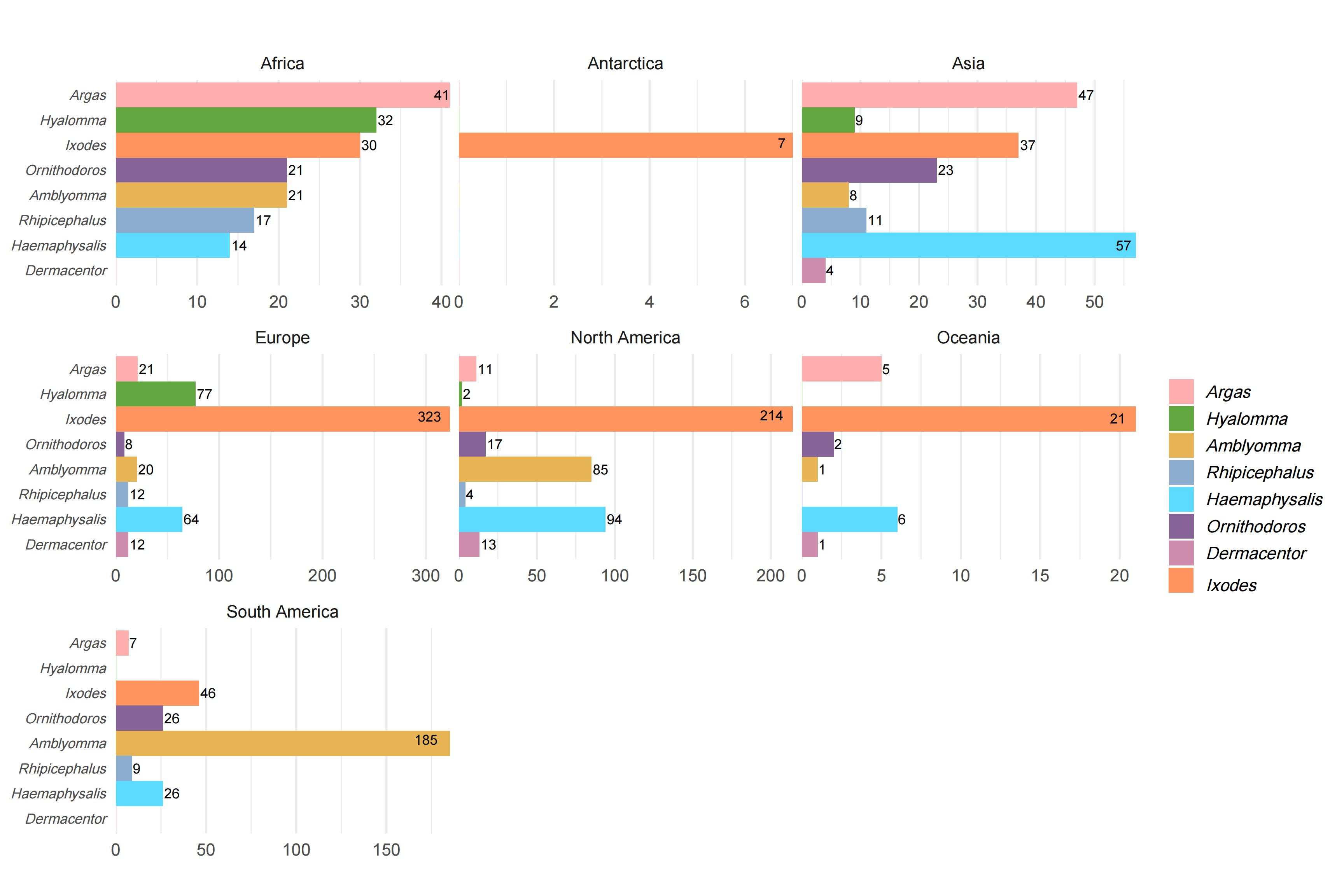


**(Ⅱ) Continental distribution of tick genera**


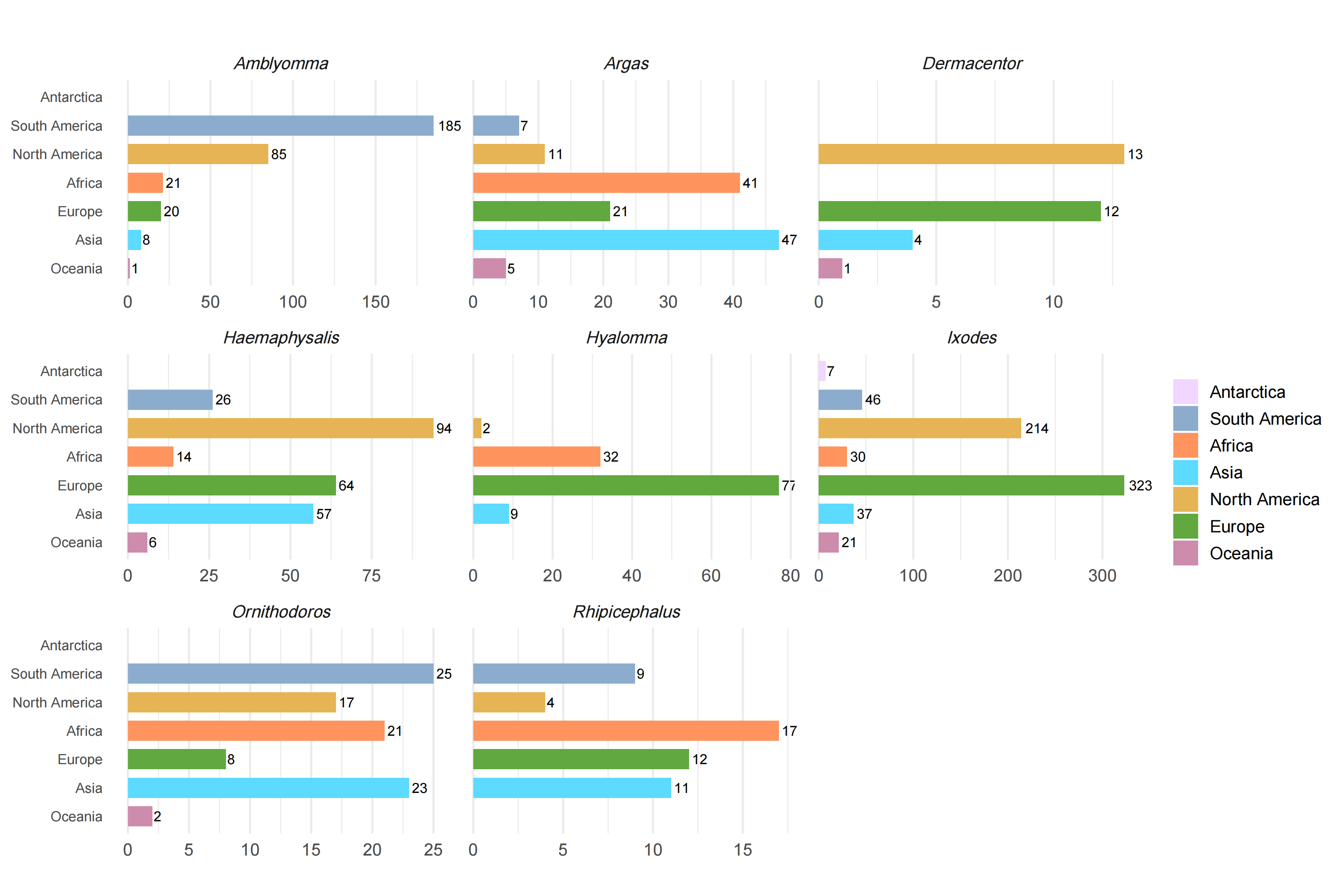


Table S6: Number of bird–tick reports by continent

Here, a “report” was defined as a unique georeferenced bird–tick occurrence, specified by the combination of geographic coordinates, avian order, and tick species. Genus-level report counts therefore represent the number of such unique records rather than the number of sampling sites or tick specimens.

| Continents | Tick genus | Number of tick species | Number of avian orders | Reports |
| --- | --- | --- | --- | --- |
| Africa |  |  |  |  |
|  | *Amblyomma* | 3 | 17 | 43 |
|  | *Argas* | 4 | 13 | 45 |
|  | *Haemaphysalis* | 7 | 3 | 23 |
|  | *Hyalomma* | 9 | 20 | 98 |
|  | *Ixodes* | 10 | 8 | 40 |
|  | *Ornithodoros* | 2 | 7 | 23 |
|  | *Rhipicephalus* | 10 | 8 | 31 |
| Antarctica |  |  |  |  |
|  | *Ixodes* | 1 | 1 | 7 |
| Asia |  |  |  |  |
|  | *Amblyomma* | 4 | 2 | 10 |
|  | *Argas* | 5 | 7 | 54 |
|  | *Dermacentor* | 3 | 2 | 4 |
|  | *Haemaphysalis* | 20 | 10 | 128 |
|  | *Hyalomma* | 2 | 4 | 13 |
|  | *lxodes* | 18 | 8 | 80 |
|  | *Ornithodoros* | 4 | 7 | 32 |
|  | *Rhipicephalus* | 5 | 4 | 11 |
| Europe |  |  |  |  |
|  | *Amblyomma* | 11 | 7 | 35 |
|  | *Argas* | 6 | 6 | 33 |
|  | *Dermacentor* | 4 | 6 | 16 |
|  | *Haemaphysalis* | 6 | 11 | 104 |
|  | *Hyalomma* | 6 | 15 | 176 |
|  | *lxodes* | 20 | 22 | 565 |
|  | *Ornithodoros* | 3 | 4 | 8 |
|  | *Rhipicephalus* | 8 | 8 | 25 |
| North America |  |  |  |  |
|  | *Amblyomma* | 21 | 10 | 188 |
|  | *Argas* | 6 | 5 | 14 |
|  | *Dermacentor* | 1 | 5 | 15 |
|  | *Haemaphysalis* | 4 | 6 | 107 |
|  | *Hyalomma* | 1 | 1 | 2 |
|  | *Ixodes* | 15 | 15 | 375 |
|  | *Ornithodoros* | 4 | 7 | 18 |
|  | *Rhipicephalus* | 1 | 1 | 3 |
| Oceania |  |  |  |  |
|  | *Amblyomma* | 1 | 3 | 3 |
|  | *Argas* | 2 | 3 | 6 |
|  | *Dermacentor* | 1 | 1 | 1 |
|  | *Haemaphysalis* | 1 | 2 | 6 |
|  | *lxodes* | 5 | 7 | 24 |
|  | *Ornithodoros* | 1 | 3 | 4 |
| South America |  |  |  |  |
|  | *Amblyomma* | 23 | 19 | 514 |
|  | *Argas* | 5 | 4 | 7 |
|  | *Haemaphysalis* | 2 | 3 | 30 |
|  | *lxodes* | 10 | 9 | 72 |
|  | *Ornithodoros* | 5 | 5 | 27 |
|  | *Rhipicephalus* | 2 | 5 | 11 |
|  | *Amblyomma* | 23 | 19 | 514 |

Table S7: Zoonotic BATBPs

| Species | Source of detection | Reference |
| --- | --- | --- |
| *Orthoflavivirus* |  |  |
| Louping ill virus | Birds | Davidson MM, Williams H, Macleod JA. Louping ill in man: a forgotten disease. *J Infect*. 1991;23(3):241-249. |
| Powassan virus | Birds | Blatman Z, Rowan-Legg A, Schaffzin JK, Wilson N, Bechard N. Powassan virus encephalitis in a 9-year-old. *CMAJ*. 2024;196(28):E973-E976. |
| Tick-borne encephalitis virus | Birds and ticks | Sigfrid L, Chan XHS, Kasbergen LMR, et al. Prevalence, clinical management, and outcomes of adults hospitalised with endemic arbovirus illness in southeast Europe (MERMAIDS-ARBO): a prospective observational study. *Lancet Infect Dis*. 2025;25(6):690-700. |
| West Nile virus | Birds and ticks | Schwartz FW, Ibaraki M, Hort HM. Seasonal Bird Migration Could Explain Regional Synchronicity and Amplification in Human West Nile Virus Case Numbers. *Geohealth*. 2025;9(3):e2024GH001194. |
| *Orthonairovirus* |  |  |
| *Orthonairovirus haemorrhagiae* | Birds and ticks | Adesola RO, Warsame AAA, Idris I. Current status of Crimean-Congo hemorrhagic fever outbreaks in Uganda and other African countries. *Health Sci Rep*. 2023;6(7):e1383. |
| Avalon Virus | Birds and ticks | Suinat JL, Leon A, Rendoing J. Polyradiculonévrite après morsures de tiques. Rôle possible d'un nouvel agent pathogène chez l'homme: le virus Avalon [Polyradiculoneuritis after tick bites. Possible role of a new pathogenic agent in man: the Avalon virus]. *Presse Med*. 1985;14(30):1616. |
| Soldado virus | Birds and ticks | Chastel C, Bailly-Choumara H, Le Lay G. Pouvoir pathogène naturel pour l'homme d'un variant antigénique du virus Soldado isolé au Maroc [Natural pathogenicity for man of an antigenic variant of Soldado virus from Morocco (author's transl)]. *Bull Soc Pathol Exot Filiales*. 1981;74(5):499-505. |
| *Bandavirus* |  |  |
| Severe fever with thrombocytopenia syndrome virus | Birds and ticks | Liu Q, He B, Huang SY, Wei F, Zhu XQ. Severe fever with thrombocytopenia syndrome, an emerging tick-borne zoonosis. *Lancet Infect Dis*. 2014;14(8):763-772. |
| *Orbivirus* |  |  |
| Kemerovo virus | Birds and ticks | Dilcher M, Hasib L, Lechner M, et al. Genetic characterization of Tribeč virus and Kemerovo virus, two tick-transmitted human-pathogenic Orbiviruses. *Virology*. 2012;423(1):68-76. |
| *Anaplasma* |  |  |
| *Anaplasma phagocytophilum* | Birds and ticks | Rikihisa Y. *Anaplasma phagocytophilum* and *Ehrlichia chaffeensis*: subversive manipulators of host cells. *Nat Rev Microbiol*. 2010;8(5):328-339. |
| *Ehrlichia* |  |  |
| *Ehrlichia chaffeensis* | Birds and ticks | Scolarici MJ, Kuehler D, Osborn R, et al. Donor-Derived Ehrlichiosis Caused by *Ehrlichia chaffeensis* from Living Donor Kidney Transplant. *Emerg Infect Dis*. 2025;31(3):587-590. |
| *Borrelia* |  |  |
| *Borrelia duttonii* | Birds and ticks | Lescot M, Audic S, Robert C, et al. The genome of *Borrelia recurrentis*, the agent of deadly louse-borne relapsing fever, is a degraded subset of tick-borne *Borrelia duttonii*. *PLoS Genet*. 2008;4(9):e1000185. |
| *Borrelia miyamotoi* | Birds and ticks | Koetsveld J, Wagemakers A, Brouwer M, et al. Limited evidence of infection with other tick-borne pathogens in patients tested for Lyme neuroborreliosis in the Netherlands. *Ticks Tick Borne Dis*. 2024;15(6):102415. |
| *Borrelia burgdorferi* sensu lato | Birds and ticks | Stanek G, Wormser GP, Gray J, Strle F. Lyme borreliosis. *Lancet*. 2012;379(9814):461-473. |
| *Borrelia burgdorferi* sensu stricto | Birds and ticks | Jungnick S, Margos G, Rieger M, et al. *Borrelia burgdorferi* sensu stricto and *Borrelia afzelii*: Population structure and differential pathogenicity. *Int J Med Microbiol*. 2015;305(7):673-681. |
| *Borrelia garinii* | Birds and ticks | Tian J, Liu J, Zhao H, et al. Molecular surveillance reveals a potential hotspot of tick-borne disease in Yakeshi City, Inner Mongolia. *BMC Microbiol*. 2023;23(1):359. |
| *Coxiella* |  |  |
| *Coxiella burnetii* | Birds and ticks | Eldin C, Mélenotte C, Mediannikov O, et al. From Q Fever to *Coxiella burnetii* Infection: a Paradigm Change. *Clin Microbiol Rev*. 2017;30(1):115-190. |
| *Francisella* |  |  |
| *Francisella tularensis* | Birds and ticks | Pechous RD, McCarthy TR, Zahrt TC. Working toward the future: insights into *Francisella tularensis* pathogenesis and vaccine development. *Microbiol Mol Biol Rev*. 2009;73(4):684-711. |
| *Rickettsia* |  |  |
| *Rickettsia africae* | Birds and ticks | Smit A, Mulandane FC, Wójcik SH, et al. Infection Rates and Characterisation of *Rickettsia africae* (Rickettsiaceae) Detected in *Amblyomma* Species from Southern Africa. *Microorganisms*. 2024;12(8):1663. |
| *Rickettsia helvetica* | Birds and ticks | Dobler G, Wölfel R. Typhus and other rickettsioses: emerging infections in Germany. *Dtsch Arztebl Int*. 2009;106(20):348-354. |
| *Rickettsia monacensis* | Birds and ticks | Jado I, Oteo JA, Aldámiz M, et al. *Rickettsia monacensis* and human disease, Spain. *Emerg Infect Dis*. 2007;13(9):1405-1407. |
| *Rickettsia parkeri* | Birds and ticks | Paddock CD, Finley RW, Wright CS, et al. *Rickettsia parkeri* rickettsiosis and its clinical distinction from Rocky Mountain spotted fever. *Clin Infect Dis*. 2008;47(9):1188-1196. |
| *Rickettsia rickettsii* | Birds and ticks | Rocky Mountain Spotted Fever in a Large Metropolitan Center, Mexico-United States Border, 2009-2019. *Pediatr Infect Dis J*. 2021;40(9):845. |
| *Orthonairovirus* |  |  |
| Beiji nairovirus | Ticks | Wang Y, Huang J, Chen M, et al. Development of a loop-mediated isothermal amplification (LAMP) assay for the rapid detection of Beiji nairovirus. *Microb Pathog*. 2025;199:107251. |
| Yezo virus | Ticks | Zhang MZ, Bian C, Ye RZ, et al. A series of patients infected with the emerging tick-borne Yezo virus in China: an active surveillance and genomic analysis. *Lancet Infect Dis*. 2025;25(4):390-398. |
| Zirqa virus | Ticks | Hoogstraal H, Gallagher MD. Blisters, pruritus, and fever after bites by the Arabian tick *Ornithodoros* (Alectorobius) *muesebecki*. *Lancet*. 1982;2(8293):288-289. |
| *Anaplasma* |  |  |
| *Anaplasma bovis* | Ticks | Lu M, Chen Q, Qin X, et al. *Anaplasma bovis* Infection in Fever and Thrombocytopenia Patients - Anhui Province, China, 2021. *China CDC Wkly*. 2022;4(12):249-253. |
| *Candidatus* Neoehrlichia |  |  |
| *Neoehrlichia mikurensis* | Ticks | Boyer PH, Baldinger L, Degeilh B, et al. The emerging tick-borne pathogen *Neoehrlichia mikurensis*: first French case series and vector epidemiology. *Emerg Microbes Infect*. 2021;10(1):1731-1738. |
| *Ehrlichia* |  |  |
| *Ehrlichia canis* | Ticks | Sgroi G, D'Alessio N, Veneziano V, et al. *Ehrlichia canis* in Human and Tick, Italy, 2023. *Emerg Infect Dis*. 2024;30(12):2651-2654. |
| *Ehrlichia ewingii* | Ticks | Adams SN, Bestul NC, Calloway KN, Kersh GJ, Salzer JS. National Surveillance of Human Ehrlichiosis Caused by *Ehrlichia ewingii*, United States, 2013-2021. *Emerg Infect Dis*. 2025;31(2):222-227. |
| *Ehrlichia muris* | Ticks | Johnson DK, Schiffman EK, Davis JP, et al. Human Infection with *Ehrlichia muris*-like Pathogen, United States, 2007-2013(1). *Emerg Infect Dis*. 2015;21(10):1794-1799. |
| *Bartonella* |  |  |
| *Bartonella grahamii* | Ticks | Xu A, Lu L, Zhang W, et al. Microevolution of *Bartonella grahamii* driven by geographic and host factors. *mSystems*. 2024;9(10):e0108924. |
| *Borrelia* |  |  |
| *Borrelia lonestari* | Ticks | Vazquez Guillamet LJ, Marx GE, Benjamin W, et al. Relapsing Fever Caused by *Borrelia lonestari* after Tick Bite in Alabama, USA. *Emerg Infect Dis*. 2023;29(2):441-444. |
| *Borrelia microti* | Ticks | Mancini F, Innocenti P, Baumgartner M, et al. *Borrelia microti* infection in an Italian woman returning from Kyrgyzstan and Tajikistan. *Travel Med Infect Dis*. 2020;35:101448. |
| *Borrelia turicatae* | Ticks | Xu A, Lu L, Zhang W, et al. Microevolution of *Bartonella grahamii* driven by geographic and host factors. *mSystems*. 2024;9(10):e0108924. |
| *Borrelia afzelii* | Ticks | Gallais F, De Martino SJ, Sauleau EA, et al. Multilocus sequence typing of clinical *Borreliella afzelii* strains: population structure and differential ability to disseminate in humans. *Parasit Vectors*. 2018;11(1):374. |
| *Borrelia andersonii* | Ticks | Clark KL, Hartman S. PCR Detection of *Bartonella* spp. and *Borreliella* spp. DNA in Dry Blood Spot Samples from Human Patients. *Pathogens*. 2024;13(9):727. |
| *Borrelia bavariensis* | Ticks | Becker NS, Rollins RE, Nosenko K, et al. High conservation combined with high plasticity: genomics and evolution of Borrelia bavariensis. *BMC Genomics*. 2020;21(1):702. |
| *Borrelia lusitaniae* | Ticks | Vieira JP, Brito MJ, de Carvalho IL. *Borrelia lusitaniae* Infection Mimicking Headache, Neurologic Deficits, and Cerebrospinal Fluid Lymphocytosis. *J Child Neurol*. 2019;34(12):748-750. |
| *Borrelia spielmanii* | Ticks | Del Giudice P, Freychet F, Kopec L, et al. Erythema Migrans Caused by *Borrelia spielmanii*, France. *Emerg Infect Dis*. 2023;29(11):2366-2369. |
| *Rickettsia* (spotted fever group) |  |  |
| *Rickettsia aeschlimannii* | Ticks | Raoult D, Fournier PE, Abboud P, Caron F. First documented human Rickettsia aeschlimannii infection. *Emerg Infect Dis*. 2002;8(7):748-749. |
| *Rickettsia conorii* | Ticks | Rovery C, Raoult D. Mediterranean spotted fever. *Infect Dis Clin North Am*. 2008;22(3):515-ix. |
| *Rickettsia felis* | Ticks | Teng Z, Zhao N, Ren R, et al. Human *Rickettsia felis* infections in Mainland China. *Front Cell Infect Microbiol*. 2022;12:997315. |
| *Rickettsia honei* | Ticks | Dehhaghi M, Kazemi Shariat Panahi H, Holmes EC, Hudson BJ, Schloeffel R, Guillemin GJ. Human Tick-Borne Diseases in Australia. *Front Cell Infect Microbiol*. 2019;9:3. |
| *Rickettsia japonica* | Ticks | Kasama K, Fujita H, Yamamoto S, et al. Genomic Features of *Rickettsia heilongjiangensis* Revealed by Intraspecies Comparison and Detailed Comparison With *Rickettsia japonica*. *Front Microbiol*. 2019;10:2787. |
| *Rickettsia massiliae* | Ticks | Zaharia M, Popescu CP, Florescu SA, et al. *Rickettsia massiliae* infection and SENLAT syndrome in Romania. *Ticks Tick Borne Dis*. 2016;7(5):759-762. |
| *Rickettsia philipii* | Ticks | Padgett KA, Bonilla D, Eremeeva ME, et al. The Eco-epidemiology of Pacific Coast Tick Fever in California. *PLoS Negl Trop Dis*. 2016;10(10):e0005020. |
| *Rickettsia sibirica* | Ticks | Santibáñez S, Ramos-Rincón JM, Santibáñez P, et al. Rickettsia sibirica mongolitimonae Infections in Spain and Case Review of the Literature. *Emerg Infect Dis*. 2025;31(1):18-26. |
| *Rickettsia slovaca* | Ticks | Sayfullin RF, Perekopskaya NE, Karan LS, Zvereva NN, Sayfullin MA. Autochthonous Case of *Rickettsia slovaca* Infection in Russia. *Emerg Infect Dis*. 2021;27(10):2736-2738. |
| *Candidatus* Rickettsia paranaensis | Ticks | Borsoi ABP, Bitencourth K, de Oliveira SV, Amorim M, Gazêta GS. Human Parasitism by *Amblyomma parkeri* Ticks Infected with *Candidatus* Rickettsia paranaensis, Brazil. *Emerg Infect Dis*. 2019;25(12):2339-2341. |
| *Babesia* |  |  |
| *Babesia divergens* | Ticks | Lobo CA, Cursino-Santos JR, Singh M, Rodriguez M. *Babesia divergens*: A Drive to Survive. *Pathogens*. 2019;8(3):95. Published 2019 Jul 2. |
| *Babesia microti* | Ticks | Madison-Antenucci S, Kramer LD, Gebhardt LL, Kauffman E. Emerging Tick-Borne Diseases. *Clin Microbiol Rev*. 2020;33(2):e00083-18. |
| *Babesia odocoilei* | Ticks | Maggi RG, Calchi AC, Moore CO, Kingston E, Breitschwerdt EB. Human *Babesia odocoilei* and *Bartonella* spp. co-infections in the Americas. *Parasit Vectors*. 2024;17(1):302. |
| *Babesia venatorum* | Ticks | Spoorenberg N, Köhler CF, Vermeulen E, et al. Autochthonous Human Babesiosis Caused by *Babesia venatorum*, the Netherlands. *Emerg Infect Dis*. 2024;30(9):1934-1938. |
| *Toxoplasma* |  |  |
| *Toxoplasma gondii* | Ticks | Matta SK, Rinkenberger N, Dunay IR, Sibley LD. *Toxoplasma gondii* infection and its implications within the central nervous system. *Nat Rev Microbiol*. 2021;19(7):467-480. |

Figure S6: Global distribution of non-zoonotic BATBPs

Hollow symbols indicate records with confirmed sampling coordinates, whereas solid symbols represent polygon-based records.

**( Ⅰ ) Distribution of Bacteria**


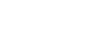

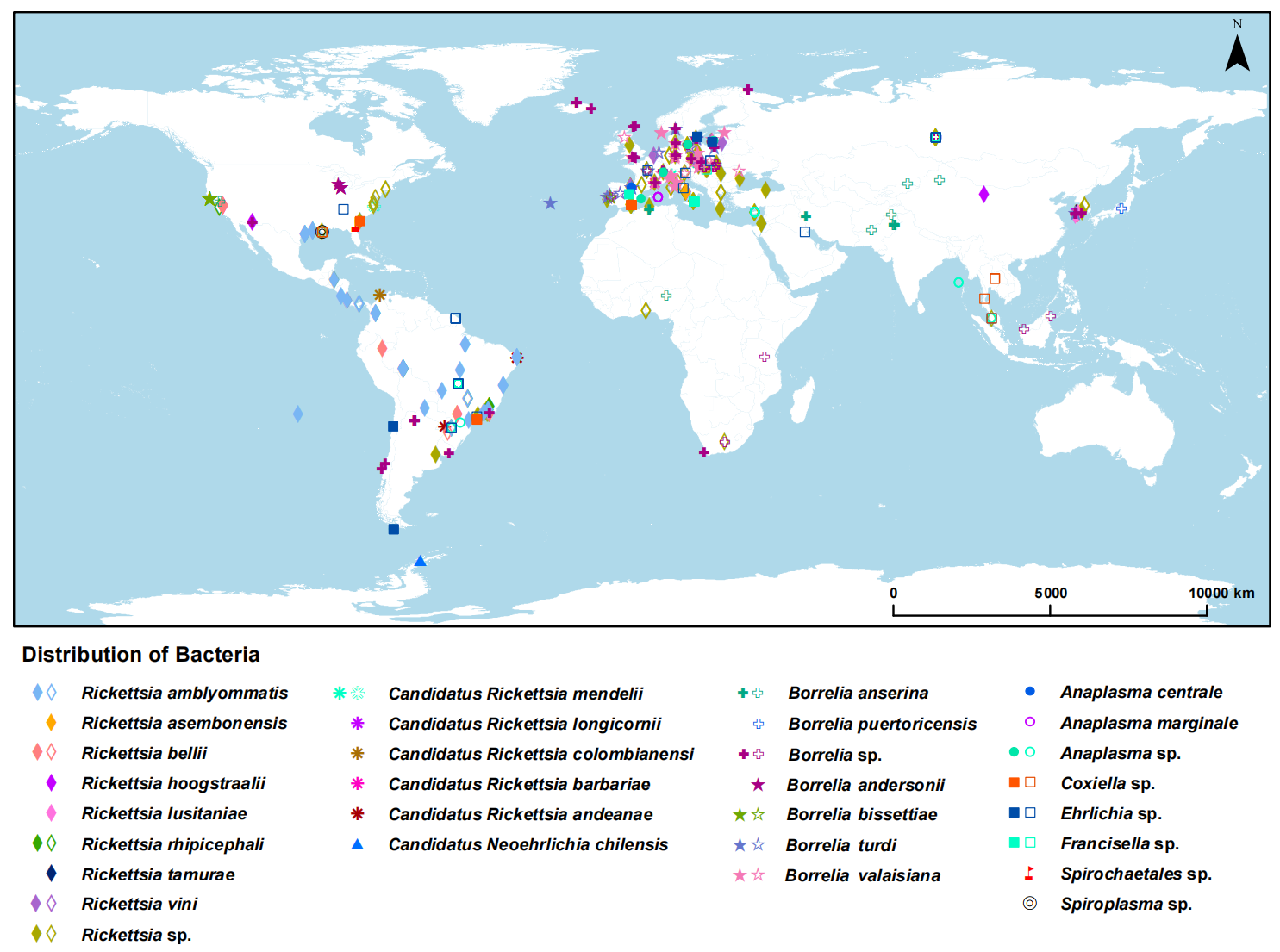


**(Ⅱ) Distribution of Virus**


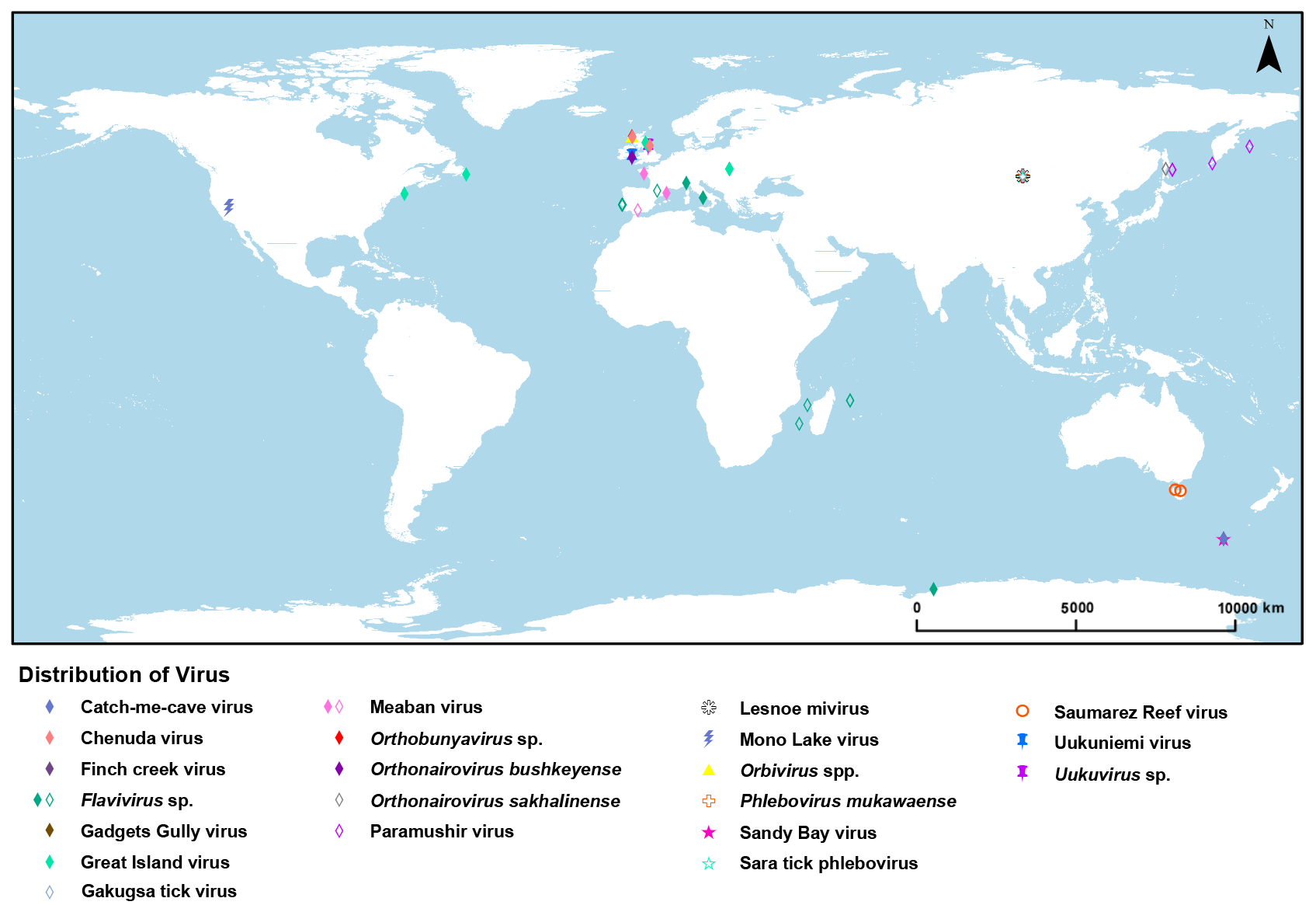


**(Ⅲ) Distribution of Protist**


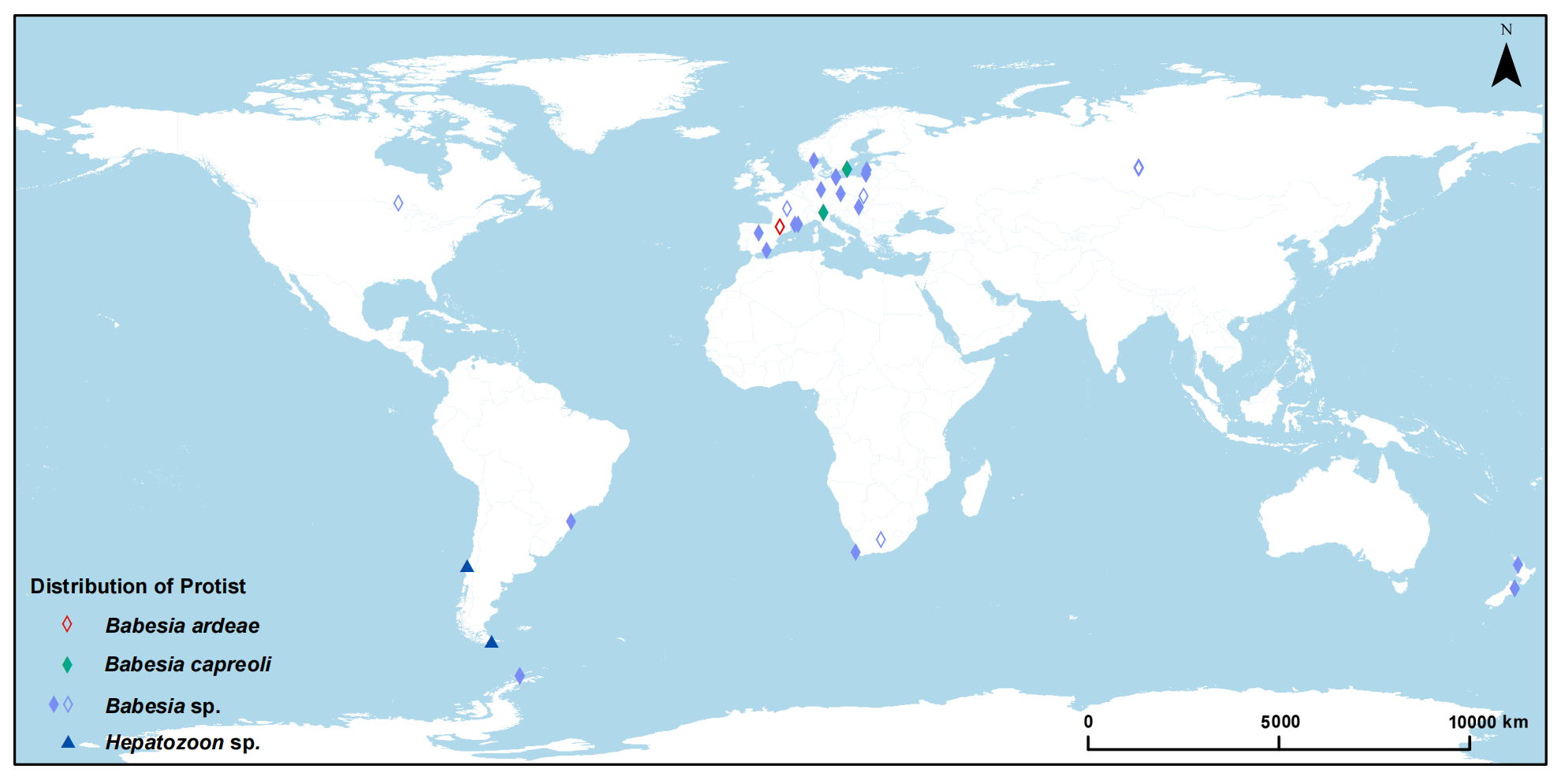


Figure S7: Global distribution of BATBPs (GenBank)

Solid symbols indicate detections in avian samples, and hollow symbols indicate detections in tick samples. ^a^ indicates zoonotic BATBP species.


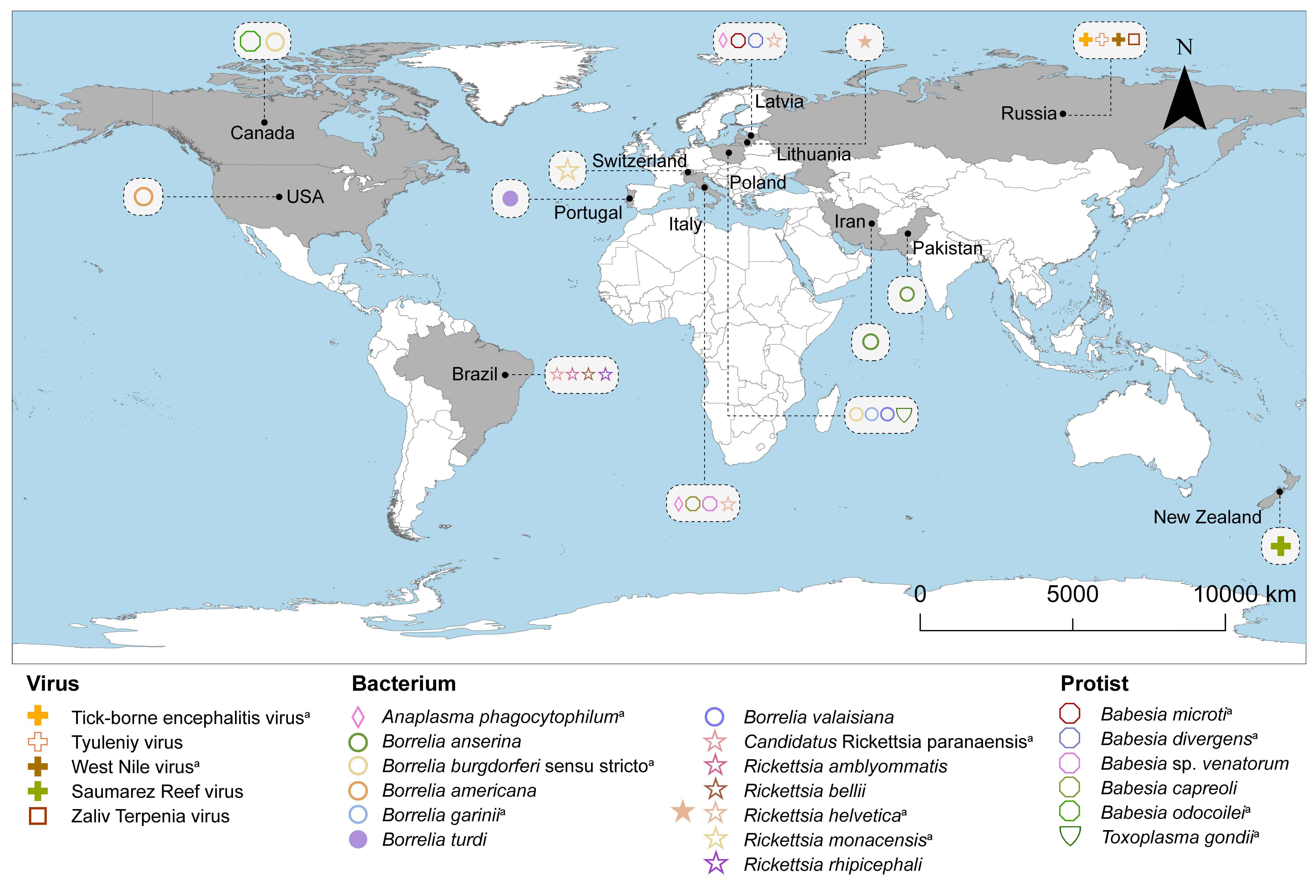

Supplement: Supplementary file 3 — Additional file 3: Figure S1. Global distribution of birds associated with ticks. Figure S2. Continental distribution and composition of bird groups. Figure S3. Global distribution of ornithophilic ticks. Figure S4. Global distribution of BATs. Figure S5. Continental distribution and composition of BATs. Table S6. Number of bird–tick reports by continent. Table S7. Zoonotic BATBPs. Figure S6. Global distribution of non-zoonotic BATBPs. Figure S7. Global distribution of BATBPs (GenBank). [file 13071_2025_7238_MOESM3_ESM.docx]
