# Supplementary material for: From flyways to foci: a systematic review and meta-analysis on the role of birds in the maintenance and global dispersal of ticks and tick-borne pathogens
Source: Parasit Vectors. 2026 Jan 24;19:88. doi: 10.1186/s13071-025-07238-4 (PMC12914891; doi:10.1186/s13071-025-07238-4)
Supplement: Supplementary file 4 — Additional file 4: Figure S8. Relationship matrix between tick species and avian. Table S8. Estimated tick infestation prevalence. Table S9. Infestation prevalence as reported in individual studies. Figure S9. Meta-analysis of tick infestation prevalence by tick genus across avian orders. Table S10. Subgroup analysis of tick infestation prevalence. [file 13071_2025_7238_MOESM4_ESM.docx]

Figure S8: Relationship matrix between tick species and avian

The tick species with documented human-biting records are labeled in red.





Table S8: Estimated tick infestation prevalence

| **Bird Order** | **Pooled Prevalence** | **95% CI** | **95% PI** | ***I^2^*** | ***τ²*** | **Studies** | **Birds examined** |
| --- | --- | --- | --- | --- | --- | --- | --- |
| Passeriformes | 0.1293 | 0.1034–0.1606 | 0.0127–0.6325 | 0.9947 | 1.5148 | 113 | 278857 |
| Bucerotiformes | 0.0146 | 0.0024–0.0827 | 0.0007–0.2282 | 0.3845 | 0.5329 | 4 | 510 |
| Caprimulgiformes | 0.0188 | 0.0004–0.4686 | 0.0001–0.8796 | 0.4248 | 1.0793 | 3 | 231 |
| Coraciiformes | 0.0456 | 0.0146–0.1337 | 0.0032–0.4163 | 0.6487 | 1.0677 | 8 | 646 |
| Cuculiformes | 0.0805 | 0.0342–0.1780 | 0.0123–0.3819 | 0.4126 | 0.5190 | 8 | 192 |
| Piciformes | 0.0515 | 0.0350–0.0751 | 0.0109–0.2118 | 0.4612 | 0.5676 | 32 | 1399 |
| Columbiformes | 0.0299 | 0.0166–0.0532 | 0.0001–0.9158 | 0.9641 | 7.8141 | 27 | 3166 |
| Galliformes | 0.1764 | 0.1022–0.2871 | 0.0095–0.8270 | 0.9919 | 2.2157 | 29 | 18746 |
| Tinamiformes | 0.1176 | NA | NA | NA | NA | 1 | 51 |
| Anseriformes | 0.1477 | 0.0159–0.6496 | 0.0033–0.9019 | 0.8938 | 1.9370 | 6 | 304 |
| Procellariiformes | 0.1010 | NA | NA | NA | NA | 1 | 594 |
| Sphenisciformes | 0.0509 | 0.0121–0.1897 | 0.0029–0.4991 | 0.9275 | 1.0740 | 6 | 1939 |
| Charadriiformes | 0.0584 | 0.0158–0.1938 | 0.0016–0.7115 | 0.8528 | 2.1934 | 9 | 659 |
| Gruiformes | 0.0769 | NA | NA | NA | NA | 1 | 91 |
| Accipitriformes | 0.0678 | 0.0131–0.2847 | 0.0010–0.8348 | 0.8111 | 2.7579 | 8 | 351 |
| Falconiformes | 0.1209 | 0.0206–0.4735 | 0.0073–0.7195 | 0.6964 | 0.6009 | 4 | 183 |
| Strigiformes | 0.0949 | 0.0426–0.1983 | 0.0266–0.2866 | 0.3506 | 0.1818 | 6 | 293 |
| Apodiformes | 0.0235 | 0.0136–0.0403 | 0.0027­0.1767 | 0.6871 | 0.9885 | 20 | 2060 |

Table S9: Infestation prevalence as reported in individual studies

| **Bird** | **Tick** | **Cases** | **Number** | **Prevalence** | **Reference ID** |
| --- | --- | --- | --- | --- | --- |
| Accipitriformes | *Amblyomma* | 11 | 12 | 0.9167 | 111 |
| Accipitriformes | *Hyalomma* | 3 | 10 | 0.1000 | 514 |
| Charadriiformes | *Ixodes* | 15 | 375 | 0.0400 | 140 |
| Charadriiformes | *Ornithodoros* | 10 | 68 | 0.1471 | 658 |
| Charadriiformes | *Haemaphysalis* | 1 | 30 | 0.0333 | 662 |
| Galliformes | *Dermacentor* | 22 | 120 | 0.1833 | 583 |
| Galliformes | *Rhipicephalus* | 26 | 284 | 0.0915 | 630 |
| Gruiformes | *Hyalomma* | 2 | 91 | 0.0220 | 133 |
| Gruiformes | *Ixodes* | 1 | 91 | 0.0110 | 133 |
| Passeriformes | *Ornithodoros* | 3 | 804 | 0.0037 | 3 |
| Piciformes | *Dermacentor* | 1 | 27 | 0.0370 | 479 |
| Procellariiformes | *Ixodes* | 60 | 584 | 0.1010 | 213 |
| Strigiformes | *Ixodes* | 3 | 30 | 0.1000 | 47 |
| Strigiformes | *Amblyomma* | 2 | 15 | 0.1333 | 112 |
| Tinamiformes | *Amblyomma* | 6 | 51 | 0.1176 | 208 |

Figure S9: Meta-analysis of tick infestation prevalence by tick genus across avian orders

The heterogeneity of combined studies was quantified by *I²* statistic.


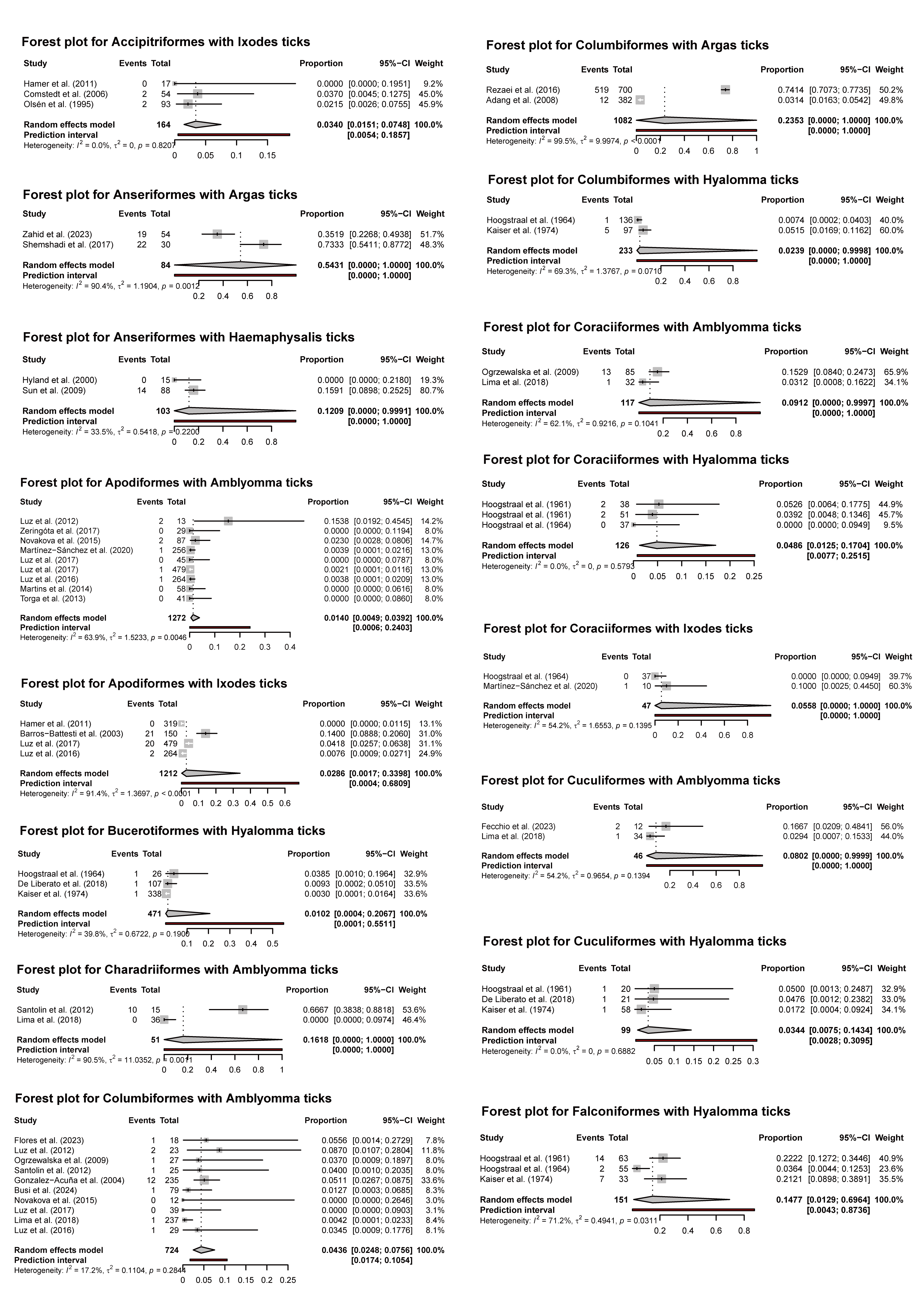


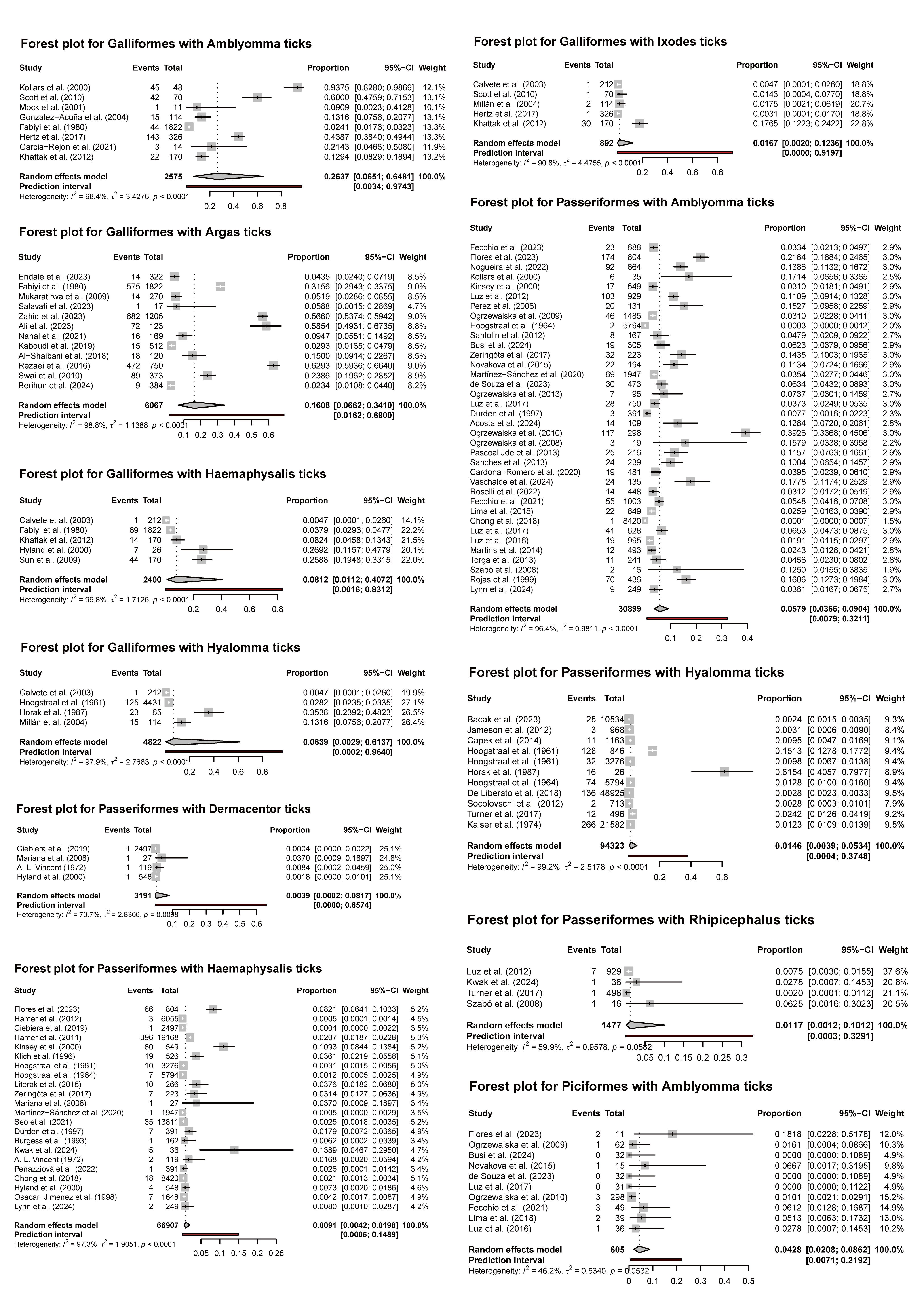


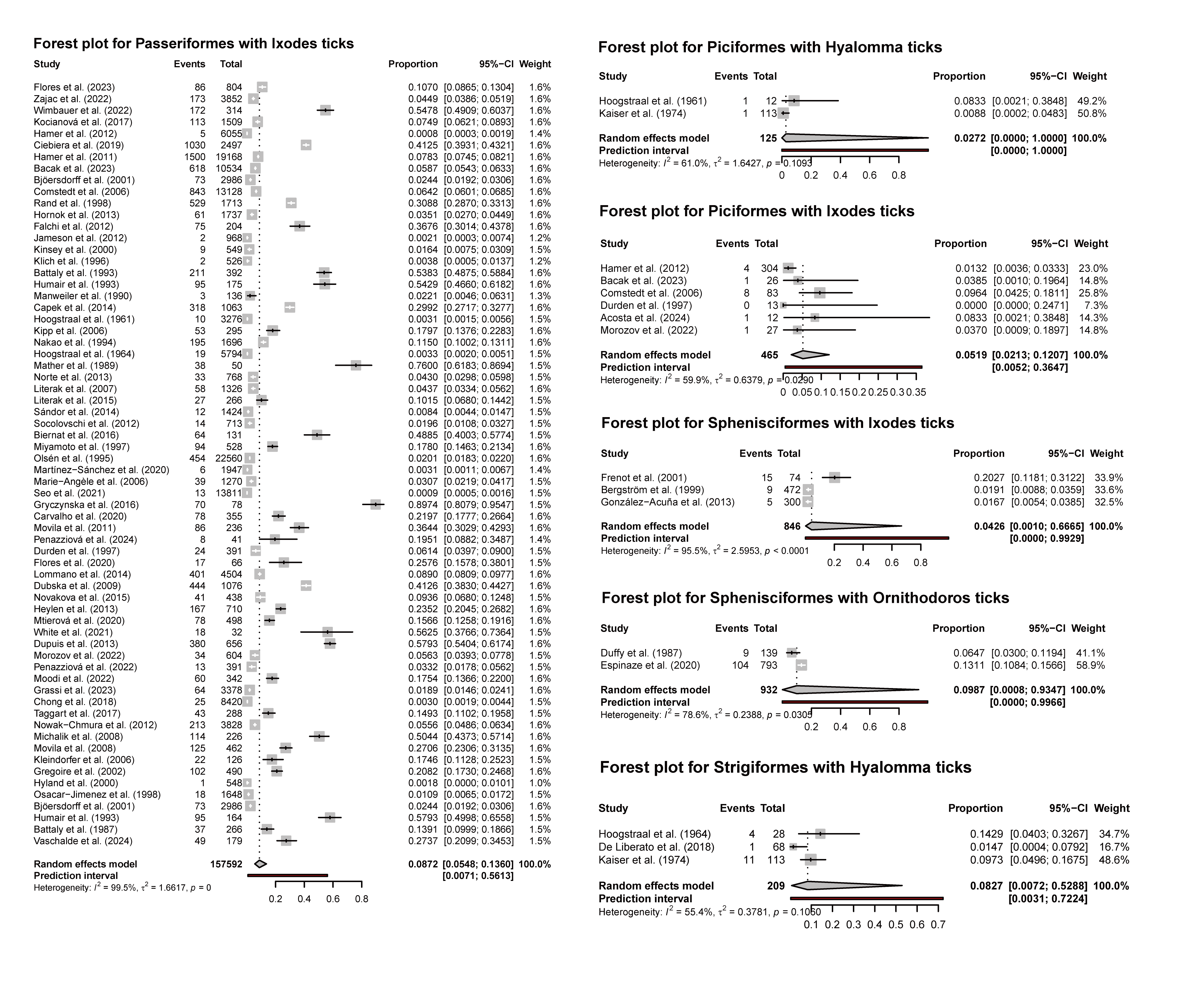


Table S10: Subgroup analysis of tick infestation prevalence

|  |  | |  | | **Prevalence** | | | | |
| --- | --- | --- | --- | --- | --- | --- | --- | --- | --- |
|  | **Studies** | **Sample size** | | **Pooled Estimate (95% CI)** | | **95% PI** | ***I^2^*** | ***τ²*** | **p value** |
| **Avian ecological groups** | **254** | **307543** | | **0.0931 (0.0785–0.1101)** | | **0.0080–0.5678** | **0.9907** | **1.6674** | **< 0.0001** |
| Songbirds | 107 | 275707 | | 0.1286 (0.1021–0.1608) | | 0.0128–0.6276 | 0.9948 | 1.4945 | .. |
| Landfowl | 52 | 22013 | | 0.0988 (0.0649–0.1477) | | 0.0044–0.7327 | 0.9885 | 2.5133 | .. |
| Climbing birds | 38 | 3216 | | 0.0470 (0.0325–0.0675) | | 0.0073–0.2481 | 0.6679 | 0.8381 | .. |
| Raptors | 15 | 877 | | 0.0809 (0.0383–0.1626) | | 0.0099–0.4363 | 0.7577 | 0.9329 | .. |
| Shorebirds | 11 | 799 | | 0.0590 (0.0208–0.1565) | | 0.0031–0.5546 | 0.8217 | 1.5733 | .. |
| Waterfowl | 12 | 2871 | | 0.0992 (0.0348–0.2520) | | 0.0101–0.5443 | 0.9344 | 1.0524 | .. |
| Aerial birds | 19 | 2060 | | 0.0238 (0.0135–0.0417) | | 0.0026–0.1845 | 0.7010 | 1.0134 | .. |
| **Continent** | **279** | **971230** | | **0.1027 (0.0871–0.1206)** | | **0.0090–0.5910** | **0.9957** | **1.6539** | **< 0.0001** |
| Oceania | 2 | 414 | | 0.1573 (0.0576–0.3633) | | 0.0324–0.5098 | 0.0000 | 0.0000 | .. |
| South America | 53 | 30917 | | 0.1223 (0.0954–0.1554) | | 0.0301–0.3843 | 0.9678 | 0.5475 | .. |
| North America | 58 | 253675 | | 0.1237 (0.0829–0.1806) | | 0.0090–0.6875 | 0.9970 | 1.8472 | .. |
| Asia | 38 | 110751 | | 0.1151 (0.0650–0.1956) | | 0.0035–0.8288 | 0.9969 | 3.1022 | .. |
| Europe | 98 | 507429 | | 0.0886 (0.0665–0.1172) | | 0.0086–0.5208 | 0.9962 | 1.4632 | .. |
| Africa | 21 | 62970 | | 0.0678 (0.0366–0.1222) | | 0.0071–0.4248 | 0.9929 | 1.1756 | .. |
| Antarctica | 3 | 585 | | 0.0120 (0.0013–0.1006) | | 0.0006–0.1927 | 0.1472 | 0.1962 | .. |
| Other | 6 | 4489 | | 0.1286 (0.0453–0.3147) | | 0.0126–0.6300 | 0.9705 | 0.7691 | .. |
| **Climatic zone** | **279** | **971230** | | **0.1027 (0.0871–0.1206)** | | **0.0090–0.5910** | **0.9957** | **1.6539** | **< 0.0001** |
| The north temperate zone | 189 | 914003 | | 0.1008 (0.0812–0.1246) | | 0.0082–0.6039 | 0.9969 | 1.7409 | .. |
| Tropics | 61 | 43463 | | 0.1002 (0.0765–0.1303) | | 0.0189–0.3921 | 0.9773 | 0.7575 | .. |
| The south temperate zone | 27 | 13340 | | 0.1422 (0.0929–0.2116) | | 0.0202–0.5713 | 0.9758 | 0.9885 | .. |
| The south frigid zone | 2 | 424 | | 0.0154 (0.0000–0.8807) | | 0.0000–0.9509 | 0.0674 | 0.0792 | .. |
| **Season** | **119** | **666690** | | **0.0722 (0.0544–0.0952)** | | **0.0063–0.4879** | **0.9964** | **1.5869** | **< 0.0001** |
| Spring | 53 | 465776 | | 0.0484 (0.0329–0.0707) | | 0.0055–0.3178 | 0.9958 | 1.1939 | .. |
| Summer | 34 | 55031 | | 0.1695 (0.0989–0.2750) | | 0.0320–0.5573 | 0.9900 | 0.7751 | .. |
| Autumn | 27 | 143642 | | 0.0552 (0.0307–0.0975) | | 0.0033–0.5081 | 0.9969 | 1.8814 | .. |
| Winter | 5 | 2241 | | 0.0650 (0.0288–0.1399) | | 0.0064–0.4281 | 0.9222 | 0.5869 | .. |
| **Habitat of sampling sites** | **276** | **968327** | | **0.1014 (0.0860–0.1193)** | | **0.0089–0.5872** | **0.9957** | **1.6508** | **< 0.0001** |
| Coastal and Islands | 72 | 506355 | | 0.0698 (0.0508–0.0952) | | 0.0080–0.4106 | 0.9966 | 1.2310 | .. |
| Forests and Woodlands | 84 | 125505 | | 0.1483 (0.1129–0.1924) | | 0.0121–0.7123 | 0.9933 | 1.7598 | .. |
| Composite Habitats | 43 | 174962 | | 0.0970 (0.0635–0.1453) | | 0.0080–0.5882 | 0.9957 | 1.6057 | .. |
| Wetlands and Aquatic Habitats | 18 | 57069 | | 0.0477 (0.0222–0.0995) | | 0.0023–0.5227 | 0.9960 | 2.0201 | .. |
| Agro-pastoral landscapes | 25 | 10170 | | 0.1389 (0.0730–0.2482) | | 0.0099–0.7225 | 0.9831 | 1.7401 | .. |
| Urban and Anthropogenic Ecosystems | 20 | 12254 | | 0.1120 (0.0504–0.2305) | | 0.0038–0.8069 | 0.9898 | 2.6542 | .. |
| Grasslands and Shrublands | 14 | 82012 | | 0.0731 (0.0352–0.1457) | | 0.0017–0.7870 | 0.9976 | 2.9521 | .. |
