# Supplementary material for: From flyways to foci: a systematic review and meta-analysis on the role of birds in the maintenance and global dispersal of ticks and tick-borne pathogens
Source: Parasit Vectors. 2026 Jan 24;19:88. doi: 10.1186/s13071-025-07238-4 (PMC12914891; doi:10.1186/s13071-025-07238-4)
Supplement: Supplementary file 5 — Additional file 5: Table S11. Co-detection of BATBPs in birds and BATs within the same study. [file 13071_2025_7238_MOESM5_ESM.docx]

Table S11: Co-detection of BATBPs in birds and BATs within the same study

| Nation | Abbreviations | Bird species | Bird order | Tick species | Tick status | Detection methods | Pathogen species | Pathogen genus | Reference ID |
| --- | --- | --- | --- | --- | --- | --- | --- | --- | --- |
| Romania | RO | *Corvus frugilegus* | Passeriformes | *Haemaphysalis concinna* | parasitising | PCR *(msp2)* | *Anaplasma phagocytophilum* | *Anaplasma* | 15 |
| Romania | RO | *Corvus monedula* | Passeriformes | *Haemaphysalis punctata* | parasitising | PCR (NA) | *Rickettsia monacensis* | *Rickettsia* | 15 |
| Romania | RO | *Corvus frugilegus* | Passeriformes | *Ixodes ricinus* | parasitising | PCR *(msp2)* | *Anaplasma phagocytophilum* | *Anaplasma* | 15 |
| Romania | RO | *Corvus monedula* | Passeriformes | *Ixodes ricinus* | parasitising | PCR (NA) | *Rickettsia monacensis* | *Rickettsia* | 15 |
| Canada | CA | *Larus argentatus* | Charadriiformes | *Ixodes uriae* | engorged | Serological test | Avalon virus | *Orthonairovirus* | 76 |
| Switzerland | CH | *Erithacus rubecula* | Passeriformes | *Ixodes ricinus* | parasitising | Isolation | *Borrelia burgdorferi* s.s. | *Borrelia* | 114 |
| USA | US | *Larus californicus* | Charadriiformes | *Argas* sp. | engorged | Isolation | Mono Lake virus | *Orbivirus* | 161 |
| USA | US | *Turdus migratorius* | Passeriformes | *Ixodes pacificus* | parasitising | PCR  *(5S-23S rRNA)* | *Borrelia burgdorferi* s.s. | *Borrelia* | 186 |
| Portugal | PT | *Turdus merula* | Passeriformes | *Haemaphysalis punctata* | parasitising | PCR  *(5S-23S rRNA)* | *Borrelia valaisiana* | *Borrelia* | 193 |
| Portugal | PT | *Turdus merula* | Passeriformes | *Ixodes frontalis* | parasitising | PCR  *(5S-23S rRNA)* | *Borrelia turdi* | *Borrelia* | 193 |
| Portugal | PT | *Turdus merula* | Passeriformes | *Ixodes ricinus* | parasitising | PCR  *(5S-23S rRNA)* | *Borrelia turdi* | *Borrelia* | 193 |
| Portugal | PT | *Turdus merula* | Passeriformes | *Ixodes ricinus* | parasitising | PCR  *(5S-23S rRNA)* | *Borrelia valaisiana* | *Borrelia* | 193 |
| Russia | RU | *Acrocephalus dumetorum* | Passeriformes | *Ixodes pavlovskyi* | parasitising | RT-PCR (NA) | Tick-borne encephalitis virus | *Orthoflavivirus* | 207 |
| Russia | RU | *Turdus iliacus* | Passeriformes | *Ixodes persulcatus* | parasitising | RT-PCR (NA) | Tick-borne encephalitis virus | *Orthoflavivirus* | 207 |
| Japan | JP | *Turdus chrysolaus* | Passeriformes | *Ixodes persulcatus* | parasitising | PCR *(flagellin*  *gene)* | *Borrelia garinii* | *Borrelia* | 233 |
| Israel | IL | *Bubulcus ibis* | Pelecaniformes | *Argas arboreus* | unidentifiable within the nest | RT-PCR (NA) | West Nile virus | *Orthoflavivirus* | 285 |
| Chile | CL | *Sphenicus magellanicus* | Sphenisciformes | *Ixodes uriae* | parasitising | PCR *(16S rRNA, dsb and groEL)* | *Ehrlichia* sp. | *Ehrlichia* | 343 |
| Germany | DE | *Luscinia megarhynchos* | Passeriformes | *Ixodes ricinus* | parasitising | PCR *(ospA)* | *Borrelia burgdorferi* | *Borrelia* | 367 |
| Hungary | HU | *Erithacus rubecula* | Passeriformes | *Haemaphysalis concinna* | parasitising | PCR *(gltA)* | *Rickettsia helvetica* | *Rickettsia* | 386 |
| Hungary | HU | *Erithacus rubecula* | Passeriformes | *Ixodes ricinus* | parasitising | PCR *(gltA)* | *Rickettsia helvetica* | *Rickettsia* | 386 |
| France | FR | *Erithacus rubecula* | Passeriformes | *Ixodes ricinus* | parasitising | PCR *(16S rRNA)* | *Ehrlichia chaffeensis* | *Ehrlichia* | 478 |
| Spain | ES | *Gyps fulvus* | Accipitriformes | *Argas persicus* | parasitising | PCR *(OmpA)* | *Rickettsia* sp. | *Rickettsia* | 545 |
| Spain | ES | *Gyps fulvus* | Accipitriformes | *Argas persicus* | parasitising | PCR *(18S rRNA)* | *Babesia ardeae* | *Babesia* | 545 |

Co-detection was defined as detection of the same pathogen in both birds and ticks sampled from the same site and within the same sampling period in a given study, with concordance assessed at the genotype/serotype level or by isolation/culture when available. Abbreviations for countries follow the ISO-3166 standard.
