# Supplementary material for: From flyways to foci: a systematic review and meta-analysis on the role of birds in the maintenance and global dispersal of ticks and tick-borne pathogens
Source: Parasit Vectors. 2026 Jan 24;19:88. doi: 10.1186/s13071-025-07238-4 (PMC12914891; doi:10.1186/s13071-025-07238-4)
Supplement: Supplementary file 6 — Additional file 6: Figure S10. Composition of ornithophilic ticks. Table S12. Distribution, hosts, and pathogens of ornithophilic ticks. Table S13. Ticks collected from bird nests or nesting cavities. Table S14. Confirmed records of tick predation by birds. Table S15. Ticks with evidence of biting humans. Table S16. Poultry infested with ticks. Table S17. Non-native ticks along migratory bird flyways. [file 13071_2025_7238_MOESM6_ESM.docx]

Figure S10: Composition of ornithophilic ticks


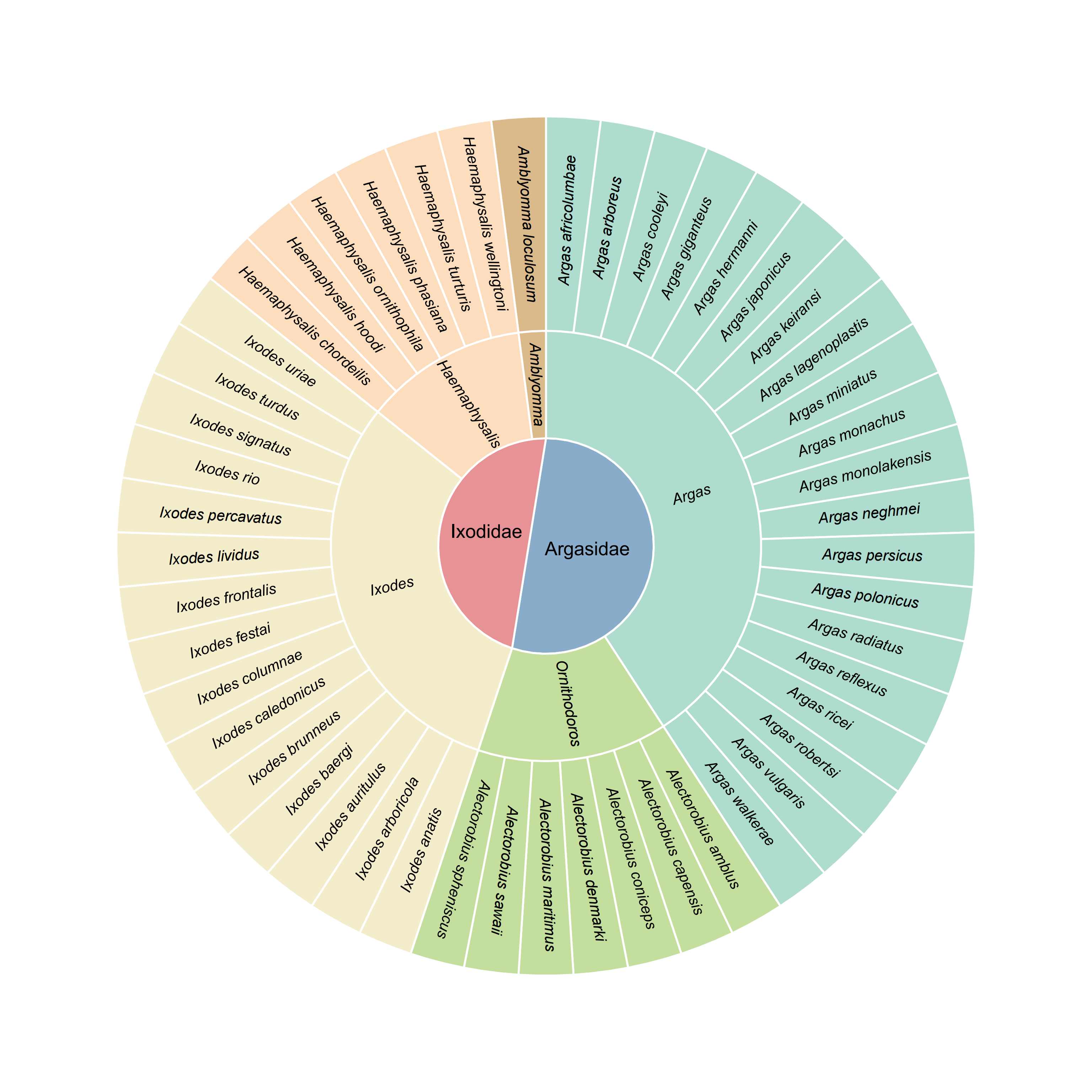


Table S12: Distribution, hosts, and pathogens of ornithophilic ticks

| Tick Species | Geographic Distribution | Representative Pathogens | Representative Hosts | Reference |
| --- | --- | --- | --- | --- |
| *Ornithodoros amblus* | the Pacific coast and on offshore islands of Peru | Not recorded | Peruvian Brown Pelican | Clifford CM, Hoogstraal H, Radovsky FJ, Stiller D, Keirans JE. Ornithodoros (Ornithodoros) amblus (Acarina: Ixodoidea: Argasidae): identity, marine bird and human hosts, virus infections, and distribution in Peru. *J Parasitol*. 1980;66(2):312-323. |
| *Ornithodoros capensis* | offshore territories worldwide | Not recorded | Seabird | Snyman A, Vanstreels RET, Nell C, et al. Determinants of external and blood parasite load in African penguins (*Spheniscus demersus*) admitted for rehabilitation. *Parasitology*. 2020;147(5):577-583. |
| *Ornithodoros coniceps* | Europe, Africa, and Asia | Baku virus | Domestic Pigeons and Swallows | Hoogstraal H, Clifford CM, Keirans JE. The Ornithodoros (Ornithodoros) capensis group (Acarina: Ixodoidea: Argasidae) of the palearctic and oriental regions. O. (A.) coniceps identity, bird and mammal hosts, virus infections, and distribution in Europe, Africa, and Asia. *J Parasitol*. 1979;65(3):395-407. |
| *Ornithodoros denmarki* | Hawaiian Archipelago | Midway virus | *Larus crassirostris* | Takahashi M, Yunker CE, Clifford CM, et al. Isolation and characterization of Midway virus: a new tick-borne virus related to Nyamanini. *J Med Virol*. 1982;10(3):181-193. |
| *Ornithodoros maritimus* | cave of Island | West Nile virus | Seabird | Sanz-Aguilar A, Payo-Payo A, Rotger A, et al. Infestation of small seabirds by Ornithodoros maritimus ticks: Effects on chick body condition, reproduction and associated infectious agents. *Ticks Tick Borne Dis*. 2020;11(1):101281. |
| *Ornithodoros sawaii* | seabird nest in Island | *Rickettsia lusitaniae* | Seabird | Kim HC, Jiang J, Hang J, et al. Detection of Rickettsia lusitaniae Among Ornithodoros sawaii Soft Ticks Collected From Japanese Murrelet Seabird Nest Material From Gugul Island, Republic of Korea. *J Med Entomol*. 2021;58(3):1376-1383. |
| *Ornithodoros spheniscus* | western South America | Not recorded | *Spheniscus humboldti* | Hoogstraal H, Wassef HY, Hays C, Keirans JE. Ornithodoros (Ornithodoros) spheniscus n. sp. [Acarina: Ixodoidea: Argasidae: Ornithodoros (Ornithodoros) capensis group], a tick parasite of the Humboldt penguin in Peru. *J Parasitol*. 1985;71(5):635-644. |
| *Amblyomma loculosum* | tropical islands of the western Indian Ocean | *Rickettsia africae* | Seabird | Dietrich M, Lebarbenchon C, Jaeger A, et al. Rickettsia spp. in seabird ticks from western Indian Ocean islands, 2011-2012. *Emerg Infect Dis*. 2014;20(5):838-842. |
| *Argas africolumbae* | Transvaal, South Africa | Not recorded | Red-rumped Swallow | Hoogstraal H, Wassef HY, Easton ER, Dixon JE. Observations on the subgenus Argas (Ixodoidea: Argasidae: Argas). 12. Argas (A.) africolumbae: variation, bird hosts, and distribution in Kenya, Tanzania, and South and South-West Africa. *J Med Entomol*. 1977;13(4-5):441-445. |
| *Argas arboreus* | Africa | West Nile virus | *Bubulcus ibis* | Mumcuoglu KY, Banet-Noach C, Malkinson M, Shalom U, Galun R. Argasid ticks as possible vectors of West Nile virus in Israel. *Vector Borne Zoonotic Dis*. 2005;5(1):65-71. |
| *Argas cooleyi* | Texas, U.S.A. | Sunday Canyon Virus | *Petrochelidon pyrrhonota* | Yunker CE, Clifford CM, Thomas LA, et al. Sunday Canyon virus, a new ungrouped agent from the tick Argas (A.) cooleyi in Texas. *Acta Virol*. 1977;21(1):36-44. |
| *Argas giganteus* | American continent | *Rickettsia hoogstraalii* | *Pandion haliaetus* | Yabsley MJ, Thompson AT, Friedeman N, et al. Detection of Rickettsia-Infected Argas (Persicargas) giganteus on Osprey (Pandion haliaetus) from the Northeastern USA. *J Wildl Dis*. |
| *Argas hermanni* | Middle East | West Nile virus | Domestic Pigeons | SCHMIDT JR, SAID MI. ISOLATION OF WEST NILE VIRUS FROM THE AFRICAN BIRD ARGASID, ARGAS REFLEXUS HERMANNI, IN EGYPT. *J Med Entomol*. 1964;1:83-86. |
| *Argas japonicus* | Japan, Korea and China | Not recorded | Swallows and Chickens | Hu X, Liu J, Bao R. Redescription and molecular characterization of the tick Argas japonicus Yamaguti, Clifford & Tipton, 1968 (Ixodida: Argasidae). *Parasitol Res*. 2021;120(11):3645-3651. |
| *Argas keiransi* | Chile | Not recorded | *Milvago* *chimango* | Estrada-Peña A, Venzal JM, González-Acuña D, Guglielmone AA. Argas (Persicargas) keiransi n. sp. (Acari: Argasidae), a parasite of the Chimango, Milvago c. chimango (Aves: Falconiformes) in Chile. *J Med Entomol*. 2003;40(6):766-769. |
| *Argas lagenoplastis* | Australia | *Rickettsia* spp. | *Petrochelidon ariel* | Diop A, Barker SC, Eberhard M, et al. Rickettsia fournieri sp. nov., a novel spotted fever group rickettsia from Argas lagenoplastis ticks in Australia. *Int J Syst Evol Microbiol*. 2018;68(12):3781-3784. |
| *Argas miniatus* | American continent | Not recorded | Chickens | Muñoz-Leal S, Venzal JM, Nava S, et al. The geographic distribution of Argas (Persicargas) miniatus and Argas (Persicargas) persicus (Acari: Argasidae) in America, with morphological and molecular diagnoses from Brazil, Chile and Cuba. *Ticks Tick Borne Dis*. 2018;9(1):44-56. |
| *Argas monachus* | Argentina and Paraguay | Not recorded | *Myiopsitta monachus* | Mastropaolo M, Turienzo P, Di Iorio O, et al. Distribution and 16S rDNA sequences of Argas monachus (Acari: Argasidae), a soft tick parasite of Myiopsitta monachus (Aves: Psittacidae). *Exp Appl Acarol*. 2011;55(3):283-291. |
| *Argas monolakensis* | California（USA） | Mono Lake virus | *Larus californicus* | Schwan TG, Corwin MD, Brown SJ. Argas (Argas) monolakensis, new species (Acari: Ixodoidea: Argasidae), a parasite of California gulls on islands in Mono Lake, California: description, biology, and life cycle. *J Med Entomol*. 1992;29(1):78-97. |
| *Argas neghmei* | Argentina and Chile | Not recorded | *Asthenes dorbignyi* | Di Iorio O, Turienzo P, Nava S, et al. Asthenes dorbignyi (Passeriformes: Furnariidae) host of Argas neghmei (Acari: Argasidae). *Exp Appl Acarol*. 2010;51(4):419-422. |
| *Argas persicus* | world | *Borrelia anserina* | *Gallus gallus* domesticus | Zahid H, Alouffi A, Almutairi MM, et al. *Argas persicus* and *Carios vespertilionis* Ticks Infesting Ducks, Domestic Fowls and Bats in Pakistan: First Report on Molecular Survey and Phylogenetic Position of *Borrelia anserina*. *Vet Sci*. 2023;10(10):628. |
| *Argas polonicus* | Krakow, Poland | Not recorded | Domestic Pigeons | Siuda K, Hoogstraal H, Clifford CM, Wassef HY. Observations on the subgenus Argas (Ixodoidea: argasidae: Argas). 17. Argas (A.) polonicus sp. n. parasitizing domestic pigeons in Krakow, Poland. *J Parasitol*. 1979;65(1):170-181. |
| *Argas radiatus* | Arizona, USA | Not recorded | *Haliaeetus leucocephalus* | Justice-Allen A, Orr K, Schuler K, McCarty K, Jacobson K, Meteyer C. Bald Eagle Nestling Mortality Associated with Argas radiatus and Argas ricei Tick Infestation and Successful Management with Nest Removal in Arizona, USA. *J Wildl Dis*. 2016;52(4):940-944. |
| *Argas reflexus* | Europe | Not recorded | Pigeons | Boni E, Incorvaia C. Near-fatal anaphylaxis with Kounis syndrome caused by *Argas reflexus* bite: a case report. *Clin Mol Allergy*. 2020;18:4. |
| *Argas ricei* | Arizona, USA | Not recorded | *Haliaeetus leucocephalus* | Justice-Allen A, Orr K, Schuler K, McCarty K, Jacobson K, Meteyer C. Bald Eagle Nestling Mortality Associated with Argas radiatus and Argas ricei Tick Infestation and Successful Management with Nest Removal in Arizona, USA. *J Wildl Dis*. 2016;52(4):940-944. |
| *Argas robertsi* | Australia | Vinegar Hill Virus | *Bulbulcus ibis* | Gauci PJ, McAllister J, Mitchell IR, Cybinski D, St George T, Gubala AJ. Genomic Characterisation of Vinegar Hill Virus, An Australian Nairovirus Isolated in 1983 from Argas Robertsi Ticks Collected from Cattle Egrets. *Viruses*. 2017;9(12):373. |
| *Argas vulgaris* | Europe | Not recorded | *Corvus frugilegus* | Palomar AM, Veiga J, Portillo A, et al. Novel Genotypes of Nidicolous *Argas* Ticks and Their Associated Microorganisms From Spain. *Front Vet Sci*. 2021;8:637837. |
| *Argas walkerae* | Africa | *Rickettsia hoogstraalii* | Chickens | Qiu Y, Simuunza M, Kajihara M, et al. Screening of tick-borne pathogens in argasid ticks in Zambia: Expansion of the geographic distribution of Rickettsia lusitaniae and Rickettsia hoogstraalii and detection of putative novel Anaplasma species. *Ticks Tick Borne Dis*. 2021;12(4):101720. |
| *Haemaphysalis chordeilis* | North America | Not recorded | *Tympanuchus phasianellus* | Egizi A, Maestas LP. Where have all the grouse ticks gone? Apparent decline in collections of *Haemaphysalis chordeilis* Packard. *Int J Parasitol Parasites Wildl*. 2022;19:323-329. |
| *Haemaphysalis hoodi* | sub-Saharan Africa | Not recorded | Not recorded | Paguem A, Mans BJ, Kingsley M, Renz A, Apanaskevich DA, Chitimia-Dobler L. *Haemaphysalis hoodi* (Acari: Ixodidae) on a human from Yaoundé, Cameroon, and its molecular characterization. *Parasitol Res*. 2022;121(10):2887-2890. |
| *Haemaphysalis ornithophila* | Southeast Asia | Not recorded | *Zoothera aurea* | Seo HJ, Noh J, Kim HC, et al. Molecular Detection and Phylogenetic Analysis of *Anaplasma* and *Borrelia* Species in Ticks Collected from Migratory Birds at Heuksan, Hong, and Nan Islands, Republic of Korea. *Vector Borne Zoonotic Dis*. 2021;21(1):20-31. |
| *Haemaphysalis phasiana* | East Asia | *Francisella*-like endosymbiont | Galliformes | Takhampunya R, Kim HC, Chong ST, et al. Francisella-Like Endosymbiont Detected in Haemaphysalis Ticks (Acari: Ixodidae) From the Republic of Korea. *J Med Entomol*. 2017;54(6):1735-1742. |
| *Haemaphysalis turturis* | India | tick-borne encephalitis virus | Not recorded | Nosek J, Kozuch O, Lichard M. Persistence of tick-borne encephalitis virus in, and its transmission by Haemaphysalis spinigera and H. turturis ticks. *Acta Virol*. 1967;11(5):479. |
| *Haemaphysalis wellingtoni* | Southeast Asia | *Anaplasma* spp. | *Gallus gallus* domesticus | Wattanamethanont J, Kaewthamasorn M, Tiawsirisup S. Natural infection of questing ixodid ticks with protozoa and bacteria in Chonburi Province, Thailand. *Ticks Tick Borne Dis*. 2018;9(3):749-758. |
| *Ixodes anatis* | New Zealand | Not recorded | Kiwi | Kwak ML, Heath ACG. Redescription of the kiwi tick Ixodes anatis (Acari: Ixodidae) from New Zealand, with notes on its biology. *Exp Appl Acarol*. 2018;74(2):207-223. |
| *Ixodes arboricola* | seabird nest in Island | *Rickettsia vini* | Not recorded | Nováková M, Heneberg P, Heylen DJA, et al. Isolated populations of Ixodes lividus ticks in the Czech Republic and Belgium host genetically homogeneous Rickettsia vini. Ticks Tick Borne Dis. 2018;9(3):479-484. |
| *Ixodes auritulus* | offshore territories | *Borrelia* spp. | Passerine Birds | Carvalho LA, Maya L, Armua-Fernandez MT, et al. Borrelia burgdorferi sensu lato infecting Ixodes auritulus ticks in Uruguay. *Exp Appl Acarol*. 2020;80(1):109-125. |
| *Ixodes baergi* | North America | Not recorded | *Hirundo pyrrhonota* | Keirans JE, Durden LA, Hopla CE. Description of the immature stages of Ixodes (Pholeoixodes) baergi (Acari: Ixodidae), a parasite of cliff swallows in the United States. *J Med Entomol*. 1993;30(4):735-739. |
| *Ixodes brunneus* | North America | *Candidatus* Rickettsia mendelii | *Mimus polyglottos* | Kennedy A, Winter W. First records of Ixodes brunneus (Acari: Ixodidae) in Delaware. *Ticks Tick Borne Dis*. 2022;13(2):101888. |
| *Ixodes caledonicus* | Europe | Not recorded | *Tachymarptis melba* | Hornok S, Kontschán J, Takács N, Pap PL, Sándor AD. First record of Ixodes (Scaphixodes) caledonicus in the Carpathian Basin and first time molecular-phylogenetic analysis of this tick species with updated host records and geographical range. *Ticks Tick Borne Dis*. 2024;15(1):102280. |
| *Ixodes columnae* | Japan | *Borrelia garinii* | *Emberiza spodocephala* | Fukunaga M, Hamase A, Okada K, et al. Characterization of spirochetes isolated from ticks (Ixodes tanuki, Ixodes turdus, and Ixodes columnae) and comparison of the sequences with those of Borrelia burgdorferi sensu lato strains. *Appl Environ Microbiol*. 1996;62(7):2338-2344. |
| *Ixodes festai* | Europe and Africa | Not recorded | Turdidae sp. | Contini C, Palmas C, Seu V, Stancampiano L, Usai F. Redescription of the male of Ixodes festai Rondelli, 1926 (Ixodida: Ixodidae) on specimens from Sardinia (Italy). *Parasite*. 2011;18(3):235-240. |
| *Ixodes frontalis* | Europe | *Rickettsia* spp. | *Turdus merula* | Plantard O, Hoch T, Daveu R, et al. Where to find questing Ixodes frontalis ticks? Under bamboo bushes!. *Ticks Tick Borne Dis*. 2021;12(2):101625. |
| *Ixodes lividus* | Europe | *Rickettsia vini* | *Riparia riparia* | Nováková M, Heneberg P, Heylen DJA, et al. Isolated populations of Ixodes lividus ticks in the Czech Republic and Belgium host genetically homogeneous Rickettsia vini. *Ticks Tick Borne Dis*. 2018;9(3):479-484. |
| *Ixodes percavatus* | Brazil | Not recorded | Procellariiformes | Acosta IDCL, Garcia IR, Luz HR, et al. New tick records with notes on rickettsial infection from the wildlife of the state of Espírito Santo, southeastern Brazil. *Ticks Tick Borne Dis*. 2024;15(2):102294. |
| *Ixodes rio* | Brazil | Not recorded | Piciformes | Acosta IDCL, Garcia IR, Luz HR, et al. New tick records with notes on rickettsial infection from the wildlife of the state of Espírito Santo, southeastern Brazil. *Ticks Tick Borne Dis*. 2024;15(2):102294. |
| *Ixodes signatus* | offshore territories | Not recorded | Seabirds | Schwan TG, Kelly PR. Ixodes signatus (Ixodoidea: Ixodidae) parasitizing Pigeon Guillemots on the Channel islands, California. *J Med Entomol*. 1981;18(2):171-172. |
| *Ixodes turdus* | Japan and Korea | *Bandavirus dabieense* | *Anthus hodgsoni* | Ji SR, Byun HR, Rieu MS, et al. First detection of Bandavirus dabieense in ticks collected from migratory birds in the Republic of Korea. *Acta Trop*. 2024;257:107279. |
| *Ixodes uriae* | offshore territories | *Borrelia burgdorferi* s.l. | Seabirds | Olsén B, Jaenson TG, Noppa L, Bunikis J, Bergström S. A Lyme borreliosis cycle in seabirds and Ixodes uriae ticks. *Nature*. 1993;362(6418):340-342. |

Table S13: Ticks collected from bird nests or nesting cavities

| Avian orders | Avian species | Tick species | Reference |
| --- | --- | --- | --- |
| Apodiformes | *Apus pacificus* | *Ornithodoros sawaii* | 577 |
| Charadriiformes | *Alca torda* | *Ixodes uriae* | 316 |
| Charadriiformes | *Cepphus grylle* | *Ixodes uriae* | 373 |
| Charadriiformes | *Fratercula arctica* | *Ixodes uriae* | 316, 619 |
| Charadriiformes | *Larus argentatus* | *Ornithodoros maritimus* | 675 |
| Charadriiformes | *Larus audouinii* | *Ornithodoros maritimus* | 658 |
| Charadriiformes | *Larus californicus* | *Argas* sp. | 161 |
| Charadriiformes | *Larus crassirostris* | *Ornithodoros capensis* | 361, 577 |
| Charadriiformes | *Larus crassirostris* | *Ornithodoros sawaii* | 326 |
| Charadriiformes | *Larus crassirostris* | *Ornithodoros* sp. | 455 |
| Charadriiformes | *Larus michahellis* | *Ornithodoros capensis* | 406, 467 |
| Charadriiformes | *Larus michahellis* | *Ornithodoros maritimus* | 553 |
| Charadriiformes | *Rissa tridactyla* | *Ixodes uriae* | 316, 669 |
| Charadriiformes | *Sterna hirundo* | *Ornithodoros capensis* | 655 |
| Charadriiformes | *Synthliboramphus antiquus* | *Ornithodoros sawaii* | 577, 590, 602 |
| Charadriiformes | *Synthliboramphus antiquus* | *Ixodes signatus* | 592 |
| Charadriiformes | *Synthliboramphus antiquus* | *Ixodes uriae* | 592 |
| Charadriiformes | *Synthliboramphus wumizusume* | *Ornithodoros sawaii* | 360 |
| Charadriiformes | *Uria aalge* | *Ixodes uriae* | 316, 619 |
| Ciconiiformes | *Egretta garzetta* | *Argas persicus* | 655 |
| Ciconiiformes | *SPhenorynchus abdimii* | *Argas arboreus* | 384 |
| Columbiformes | *Columba guinea* | *Argas africolumbae* | 157 |
| Columbiformes | *Columba livia* | *Argas reflexus* | 167, 404, 729 |
| Columbiformes | *Columbidae* sp. | *Argas hermanni* | 564 |
| Columbiformes | pigeon | *Argas reflexus* | 167, 357, 453 |
| Coraciiformes | *Coracias garrulus* | *Argas africolumbae* | 462 |
| Coraciiformes | *Coracias garrulus* | *Argas persicus* | 546 |
| Coraciiformes | *Coracias garrulus* | *Argas polonicus* | 462 |
| Coraciiformes | *Coracias garrulus* | *Argas* sp. | 462 |
| Galliformes | chicken | *Argas persicus* | 348, 649 |
| Galliformes | Domestic Fowl | *Ornithodoros puertoricensis* | 486 |
| Galliformes | laying chickens | *Argas* sp. | 625 |
| Galliformes | poultry | *Argas persicus* | 591, 527 |
| Galliformes | poultry | *Argas* sp. | 321 |
| Passeriformes | *Asthenes dorbignyi* | *Argas neghmei* | 144 |
| Passeriformes | *Cecropis daurica* | *Argas japonicus* | 158, 766 |
| Passeriformes | *Certhia* sp. | *Ixodes arboricola* | 397 |
| Passeriformes | *Corvus coronoides* | *Argas persicus* | 468 |
| Passeriformes | *Delichon urbicum* | *Argas japonicus* | 158 |
| Passeriformes | *Dendrocolaptes platyrostris* | *Ornithodoros mimon* | 3 |
| Passeriformes | *Panurus biarmicus* | *Dermacentor marginatus* | 642 |
| Passeriformes | *Parus caeruleus* | *Ixodes ricinus* | 659 |
| Passeriformes | *Petrochelidon pyrrhonota* | *Argas cooleyi* | 177, 562 |
| Passeriformes | *Poecile palustris* | *Ixodes arboricola* | 397 |
| Passeriformes | *Ptyonoprogne fuligula* | *Argas africolumbae* | 157 |
| Passeriformes | *Riparia riparia* | *Ixodes lividus* | 691 |
| Passeriformes | *Sitta europaea* | *Ixodes arboricola* | 397 |
| Passeriformes | small passerine | *Ixodes pacificus* | 531 |
| Passeriformes | swallows | *Ornithodoros coniceps* | 511 |
| Passeriformes | *Turdus merula* | *Ixodes ricinus* | 641 |
| Passeriformes | *Dendrocopos major* | *Ornithodoros mimon* | 3 |
| Pelecaniformes | *Ardeidae* spp. | *Ornithodoros capensis* | 655 |
| Pelecaniformes | *Bubulcus ibis* | *Argas arboreus* | 724 |
| Pelecaniformes | *Bubulcus ibis* | *Argas robertsi* | 132 |
| Pelecaniformes | *Pelecanus occidentalis* | *Ornithodoros capensis* | 129, 653 |
| Pelecaniformes | *Pelecanus thagus* | *Ornithodoros spheniscus* | 449 |
| Pelecaniformes | *Platalea alba* | *Argas arboreus* | 724 |
| Pelecaniformes | *Threskiornis aethiopicus* | *Argas arboreus* | 724 |
| Procellariiformes | *Hydrobates monorhis* | *Ornithodoros sawaii* | 361, 455, 577, 590, 602 |
| Sphenisciformes | *Aptenodytes patagonicus* | *Ixodes uriae* | 379, 651, 709 |
| Sphenisciformes | *Chinstrap Penguin* | *Ixodes uriae* | 709 |
| Sphenisciformes | *Eudyptes chrysocome* | *Ixodes uriae* | 379 |
| Sphenisciformes | *Eudyptes schlegeli* | *Ixodes uriae* | 379 |
| Sphenisciformes | *Pygoscelis adeliae* | *Ixodes uriae* | 620 |
| Sphenisciformes | *Pygoscelis papua* | *Ixodes uriae* | 180 |
| Sphenisciformes | *Spheniscus humboldti* | *Ornithodoros spheniscus* | 449 |
| Sphenisciformes | *Gentoo Penguin* | *Ixodes uriae* | 709 |
| Strigiformes | *Aegolius funereus* | *Ixodes arboricola* | 723 |
| Strigiformes | *Athene noctua* | *Argas reflexus* | 462 |
| Strigiformes | *Otus scops* | *Argas persicus* | 546 |
| Suliformes | *Microcarbo africanus* | *Argas arboreus* | 722 |
| Suliformes | *Phalacrocorax carbo* | *Ornithodoros capensis* | 384 |
| Suliformes | *Phalacrocorax carbo* | *Argas arboreus* | 384 |
| Suliformes | *Phalacrocorax penicillatus* | *Ixodes signatus* | 319 |
| Suliformes | *Sula leucogaster* | *Ornithodoros capensis* | 589, 689 |
| Suliformes | *Sula nebouxii* | *Ornithodoros* sp. | 164 |
| Swimmers | *Phalacrocorax punctatus* | *Ixodes* sp. | 178 |

Table S14: Confirmed records of tick predation by birds

| Avian orders | Avian species | Tick species | Tick hosts | Reference |
| --- | --- | --- | --- | --- |
| Passeriformes | *Molothrus oryzivorus* | *Amblyomma dubitatum* | *Tapirus terrestris*, *Hydrochoerus hydrochaeris* | 52 |
| Passeriformes | *Molothrus oryzivorus* | *Amblyomma triste* | *Tapirus terrestris*, *Hydrochoerus hydrochaeris* | 52 |
| Passeriformes | *Molothrus oryzivorus* | *Amblyomma sculptum* | *Tapirus terrestris*, *Hydrochoerus hydrochaeris* | 52 |
| Tinamiformes | *Nothoprocta pentlandii* | *Rhipicephalus microplus* | cattle | 166 |
| Tinamiformes | *Nothoprocta pentlandii* | *Rhipicephalus sanguineus* | dog | 166 |
| Passeriformes | *Buphagus erythrorhynchus* | Unknown | *Ceratotherium simum*, *Giraffa camelopardalis*, *Tragelaphus strepsiceros* and other ungulates | 609 |
| Passeriformes | *Buphagus africanus* | Unknown | *Ceratotherium simum*, *Giraffa camelopardalis*, *Tragelaphus strepsiceros* and other ungulates | 609 |
| Accipitriformes | *Coragyps atratus* | Unknown | *Hydrochoerus hydrochaeris*, cattle | 644 |
| Falconiformes | *Milvago chimachima* | Unknown | *Hydrochoerus hydrochaeris*, cattle | 644 |
| Passeriformes | *Molothrus oryzivorus* | Unknown | *Hydrochoerus hydrochaeris*, cattle | 644 |
| Passeriformes | *Machetornis rixosa* | Unknown | *Hydrochoerus hydrochaeris*, cattle | 644 |
| Cuculiformes | *Crotophaga ani* | Unknown | *Hydrochoerus hydrochaeris*, cattle | 645 |
| Pelecaniformes | *Bubulcus ibis* | Unknown | Large ungulates | 674 |
| Galliformes | *Numida meleagris* | Unknown | Large ungulates | 674 |
| Charadriiformes | *Vanellus coronatus* | Unknown | Large ungulates | 674 |
| Coraciiformes | *Coracias caudatus* | Unknown | Large ungulates | 674 |
| Passeriformes | *Rilipidura tricolor* | Unknown | cattle | 683 |
| Passeriformes | *Rhipidura leucophrys* | Unknown | cattle | 683 |
| Passeriformes | *Lanius senator* | *Hyalomma dromedarii* | *Camelus dromedarius* | 761 |
| Passeriformes | *Buphagus erythrorhynchus* | *Hyalomma truncatum* | *Bos taurus*, *Giraffa camelopardalis*, *Aepyceros melampus*, *Syncerus caffer*, *Hippotragus niger*, *Equus burchellii* and other mammalian species | 762 |
| Passeriformes | *Buphagus erythrorhynchus* | *Rhipicephalus decoloratus* | *Bos taurus*, *Giraffa camelopardalis*, *Aepyceros melampus*, *Syncerus caffer*, *Hippotragus niger*, *Equus burchellii* and other mammalian species | 762 |
| Passeriformes | *Buphagus erythrorhynchus* | Ixodidae sp. | *Bos taurus*, *Giraffa camelopardalis*, *Aepyceros melampus*, *Syncerus caffer*, *Hippotragus niger*, *Equus burchellii* and other mammalian species | 762 |
| Passeriformes | *Buphagus erythrorhynchus* | *Amblyomma variegatum* | *Bos taurus*, *Giraffa camelopardalis*, *Aepyceros melampus*, *Syncerus caffer*, *Hippotragus niger*, *Equus burchellii* and other mammalian species | 762 |
| Passeriformes | *Buphagus erythrorhynchus* | *Rhipicephalus evertsi* | *Bos taurus*, *Giraffa camelopardalis*, *Aepyceros melampus*, *Syncerus caffer*, *Hippotragus niger*, *Equus burchellii* and other mammalian species | 762 |
| Passeriformes | *Buphagus erythrorhynchus* | *Rhipicephalus zambeziensis* | *Bos taurus*, *Giraffa camelopardalis*, *Aepyceros melampus*, *Syncerus caffer*, *Hippotragus niger*, *Equus burchellii* and other mammalian species | 762 |
| Passeriformes | *Molothrus oryzivorus* | Unknown | *Tapirus terrestris* | 763 |

Table S15: Ticks with evidence of biting human

| Tick Species | Reference |
| --- | --- |
| *Ornithodoros amblus* | Hoogstraal H, Wassef HY, Hays C, Keirans JE. *Ornithodoros* (*Ornithodoros*) *spheniscus* n. sp. [Acarina: Ixodoidea: Argasidae: *Ornithodoros* (*Ornithodoros*) *capensis* group], a tick parasite of the Humboldt penguin in Peru. *J Parasitol*. 1985 Oct;71(5):635-44. |
| *Ornithodoros capensis* | Converse JD, Hoogstraal H, Moussa MI, Feare CJ, Kaiser MN. Soldado virus (Hughes group) from *Ornithodoros* (*Ornithodoros*) *capensis* (Ixodoidea: Argasidae) infesting Sooty Tern colonies in the Seychelles, Indian Ocean. *Am J Trop Med Hyg*. 1975 Nov;24(6 Pt 1):1010-8. |
| *Ornithodoros coniceps* | Hoogstraal H, Clifford CM, Keirans JE. The *Ornithodoros* (*Ornithodoros*) *capensis* group (Acarina: Ixodoidea: Argasidae) of the palearctic and oriental regions. *O*. (*A*.) *coniceps* identity, bird and mammal hosts, virus infections, and distribution in Europe, Africa, and Asia. *J Parasitol*. 1979 Jun;65(3):395-407. |
| *Ornithodoros mimon* | Labruna MB, Marcili A, Ogrzewalska M, Barros-Battesti DM, Dantas-Torres F, Fernandes AA, Leite RC, Venzal JM. New records and human parasitism by *Ornithodoros mimon* (Acari: Argasidae) in Brazil. *J Med Entomol*. 2014 Jan;51(1):283-7. |
| *Ornithodoros spheniscus* | Hoogstraal H, Wassef HY, Hays C, Keirans JE. *Ornithodoros* (*Ornithodoros*) *spheniscus* n. sp. [Acarina: Ixodoidea: Argasidae: *Ornithodoros* (*Ornithodoros*) *capensis* group], a tick parasite of the Humboldt penguin in Peru. *J Parasitol*. 1985 Oct;71(5):635-44. |
| *Amblyomma americanum* | Madison-Antenucci S, Kramer LD, Gebhardt LL, Kauffman E. Emerging tick-borne diseases. *Clin Microbiol Rev* 2020; 33: e00083-18. |
| *Amblyomma aureolatum* | Szabó MP, Pinter A, Labruna MB. Ecology, biology and distribution of spotted-fever tick vectors in Brazil. *Front Cell Infect Microbiol* 2013; 3: 27. |
| *Amblyomma auricularium* | Szabó MPJ, Martins TF, Barbieri ARM, et al. Ticks biting humans in the Brazilian savannah: Attachment sites and exposure risk in relation to species, life stage and season. *Ticks Tick Borne Dis*. 2020;11(2):101328. |
| *Amblyomma brasiliense* | Suzin A, da Silva MX, Tognolli MH, et al. Ticks on humans in an Atlantic rainforest preserved ecosystem in Brazil: Species, life stages, attachment sites, and temporal pattern of infestation. *Ticks Tick Borne Dis*. 2022;13(1):101862. |
| *Amblyomma cajennense* | Szabó MP, Pinter A, Labruna MB. Ecology, biology and distribution of spotted-fever tick vectors in Brazil. *Front Cell Infect Microbiol* 2013; 3: 27. |
| *Amblyomma calcaratum* | Valente JDM, Silva PW, Arzua M, et al. Records of ticks (Acari: Ixodidae) on humans and distribution of spotted-fever cases and its tick vectors in Paraná State, southern Brazil. *Ticks Tick Borne Dis*. 2020;11(6):101510. |
| *Amblyomma coelebs* | Ito K, Taniguchi H, Ohtaki N, Ando S, Kawabata H. A first case of tick bite by Amblyomma coelebs in Japan. *J Dermatol* 2018; 45: 243-4. |
| *Amblyomma dissimile* | Eisen L. Tick species infesting humans in the United States. *Ticks Tick Borne Dis*. 2022;13(6):102025. |
| *Amblyomma dubitatum* | Szabó MP, Pinter A, Labruna MB. Ecology, biology and distribution of spotted-fever tick vectors in Brazil. *Front Cell Infect Microbiol* 2013; 3: 27. |
| *Amblyomma hebraeum* | Petney TN, Horak IG, Rechav Y. The ecology of the African vectors of heartwater, with particular reference to *Amblyomma hebraeum* and *Amblyomma variegatum*. *Onderstepoort J Vet Res* 1987; 54: 381-95. |
| *Amblyomma integrum* | Dilrukshi PR, Yasawardene AD, Amerasinghe PH, Amerasinghe FP. Human otoacariasis: a retrospective study from an area of Sri Lanka. *Trans R Soc Trop Med Hyg*. 2004;98(8):489-495. |
| *Amblyomma lepidum* | Petney TN, Horak IG, Rechav Y. The ecology of the African vectors of heartwater, with particular reference to *Amblyomma hebraeum* and *Amblyomma variegatum*. *Onderstepoort J Vet Res* 1987; 54: 381-95. |
| *Amblyomma loculosum* | Eldin C, Mediannikov O, Davoust B, et al. Emergence of *Rickettsia africae*, Oceania. *Emerg Infect Dis*2011; 17:100-2. |
| *Amblyomma longirostre* | Valente JDM, Silva PW, Arzua M, et al. Records of ticks (Acari: Ixodidae) on humans and distribution of spotted-fever cases and its tick vectors in ParanáState, southern Brazil. *Ticks Tick Borne Dis*2020; 11:101510. |
| *Amblyomma maculatum* | Zemtsova GE, Watkins NE, JRhipicephalus, Levin ML. Multiplex qPCR assay for identification and differentiation of *Amblyomma americanum*, *Amblyomma cajennense*, and *Amblyomma maculatum*(Ixodida: Ixodidae) tick species in the eastern United States. *J Med Entomol*2014; 51:795-803. |
| *Amblyomma marmoreum* | Petney TN, Horak IG, Rechav Y. The ecology of the African vectors of heartwater, with particular reference to *Amblyomma hebraeum* and *Amblyomma variegatum*. *Onderstepoort J Vet Res* 1987; 54: 381-95. |
| *Amblyomma mixtum* | Novakova M, Literak I, Chevez L, et al. Rickettsial infections in ticks from reptiles, birds and humans in Honduras. *Ticks Tick Borne Dis* 2015; 6: 737-42. |
| *Amblyomma naponense* | Bermúdez CS, Castro A, Esser H, et al. Ticks (Ixodida) on humans from central Panama, Panama (2010-2011). *Exp Appl Acarol* 2012; 58: 81-8. |
| *Amblyomma ovale* | Szabó MP, Pinter A, Labruna MB. Ecology, biology and distribution of spotted-fever tick vectors in Brazil. *Front Cell Infect Microbiol* 2013; 3: 27. |
| *Amblyomma parkeri* | Valente JDM, Silva PW, Arzua M, et al. Records of ticks (Acari: Ixodidae) on humans and distribution of spotted-fever cases and its tick vectors in ParanáState, southern Brazil. *Ticks Tick Borne Dis*2020; 11:101510. |
| *Amblyomma parvum* | Monje LD, Fernandez C, PercaraA. Detection of Ehrlichiasp. strain San Luis and *Candidatus* Rickettsia andeanae in Amblyomma parvum ticks. *Ticks Tick Borne Dis*2019; 10:111-4. |
| *Amblyomma rotundatum* | Eisen L. Tick species infesting humans in the United States. *Ticks Tick Borne Dis*. 2022;13(6):102025. |
| *Amblyomma sculptum* | Saracho-Bottero MN, Tarragona EL, Sebastian PS, et al. Ticks infesting cattle and humans in the Yungas Biogeographic province of Argentina, with notes on the presence of tick-borne bacteria. *Exp Appl Acarol* 2018; 74: 107-16. |
| *Amblyomma testudinarium* | Chao LL, Lu CW, Lin YF, Shih CM. Molecular and morphological identification of a human biting tick, *Amblyomma testudinarium*(Acari: Ixodidae), in Taiwan. *Exp Appl Acarol* 2017; 71:401-14. |
| *Amblyomma tigrinum* | Nava S, Lareschi M, Rebollo C, et al. The ticks (Acari: Ixodida:Argasidae, Ixodidae) of Paraguay. *Ann Trop Med Parasitol*2007; 101:255-70. |
| *Amblyomma triste* | Romer Y, Borrás P, GovedicF, et al. Clinical and epidemiological comparison of *Rickettsia parkeri* rickettsiosis, related to *Amblyomma triste* and *Amblyomma tigrinum*, in Argentina. *Ticks Tick Borne Dis*2020; 11:101436. |
| *Amblyomma variegatum* | Petney TN, Horak IG, Rechav Y. The ecology of the African vectors of heartwater, with particular reference to *Amblyomma hebraeum* and *Amblyomma variegatum*. *Onderstepoort J Vet Res* 1987; 54: 381-95. |
| *Amblyomma varium* | Chitimia-Dobler L, Fachet K, Lindau A, et al. Exotic ticks removed from German travelers. *Parasitol Res*. 2024;123(2):120. |
| *Argas cooleyi* | Beatty NL, Klotz SA, Elliott SP. Hematophagous Ectoparasites of Cliff Swallows Invade a Hospital and Feed on Humans. *Clin Infect Dis*. 2017;65(12):2119-2121. |
| *Argas japonicus* | "Yan P, Qiu Z, Zhang T, et al. Microbial diversity in the tick *Argas japonicus* (Acari: Argasidae) with a focus on Rickettsia pathogens. *Med Vet Entomol* 2019; 33:327-35." |
| *Argas persicus* | "Dehhaghi M, Kazemi Shariat Panahi H, Holmes EC, Hudson BJ, Schloeffel R, Guillemin GJ. Human tick-borne diseases in Australia. *Front Cell Infect Microbiol* 2019; 9: 3." |
| *Argas reflexus* | Buczek A, Bartosik K, Kulina D, Raszewska-Famielec M, Borzęcki A. Skin lesions in humans bitten by European pigeon tick *Argas reflexus* (Fab.) (Ixodida: Argasidae) massively occurring in the Upper Silesian conurbation of south-west Poland. *Ann Agric Environ Med*. 2018;25(2):234-240. |
| *Argas vulgaris* | Chen Z, Liu J. A review of argasid ticks and associated pathogens of China. *Front Vet Sci*. 2022;9:865664. |
| *Dermacentor auratus* | Liyanaarachchi DR, Rajakaruna RS, Dikkumbura AW, Rajapakse RP. Ticks infesting wild and domestic animals and humans of Sri Lanka with new host records. *Acta Trop*. 2015;142:64-70. |
| *Dermacentor marginatus* | Garcia-Vozmediano A, Giglio G, RamassaE, Nobili F, Rossi L, Tomassone L. *Dermacentor marginatus* and *Dermacentor reticulatus*, and their infection by SFG *Rickettsiae* and Francisella-Like Endosymbionts, in mountain and periurban habitatsof northwesternItaly. *Vet Sci* 2020; 7: 157. |
| *Dermacentor nuttalli* | Khasnatinov MA, Liapunov AV, Manzarova EL, Kulakova NV, Petrova IV, Danchinova GA. The diversity and prevalence of hard ticks attacking human hosts in Eastern Siberia (Russian Federation) with first description of invasion of non-endemic tick species. *Parasitol Res*2016; 115:501-10. |
| *Dermacentor reticulatus* | Khasnatinov MA, Liapunov AV, Manzarova EL, Kulakova NV, Petrova IV, Danchinova GA. The diversity and prevalence of hard ticks attacking human hosts in Eastern Siberia (Russian Federation) with first description of invasion of non-endemic tick species. *Parasitol Res*2016; 115:501-10. |
| *Dermacentor variabilis* | Madison-AntenucciS, Kramer LD, Gebhardt LL, Kauffman E. Emerging tick-borne diseases. *Clin Microbiol Rev*2020; 33:e00083-18. |
| *Haemaphysalis bispinosa* | Kwak ML, Ng A. Detection of three new *Haemaphysalis* ticks (Acari: Ixodidae) in Singapore and their potential threat for public health, companion animals, and wildlife. *Acarologia*. 2022;62(4):927–940. |
| *Haemaphysalis concinna* | Khasnatinov MA, Liapunov AV, Manzarova EL, Kulakova NV, Petrova IV, Danchinova GA. The diversity and prevalence of hard ticks attacking human hosts in Eastern Siberia (Russian Federation) with first description of invasion of non-endemic tick species. *Parasitol Res*2016; 115:501-10. |
| *Haemaphysalis erinacei* | Keskin A, Keskin A, Bursali A, Tekin S. Ticks (Acari: Ixodida) parasitizing humans in Corum and Yozgat provinces, Turkey. *Exp Appl Acarol* 2015; 67: 607-16. |
| *Haemaphysalis flava* | Ozawa A, Yamaguchi N, Hayakawa K, Matsuo I, Niizuma K, Ohkido M. [A case of tick bite (*Haemaphysalis flava*)--consideration of tularemia infection through tick bite]. *Nihon Hifuka Gakkai Zasshi* 1982; 92: 1415-21. |
| *Haemaphysalis hoodi* | Paguem A, Mans BJ, Kingsley M, Renz A, Apanaskevich DA, Chitimia-Dobler L. *Haemaphysalis hoodi* (Acari: Ixodidae) on a human from Yaoundé, Cameroon, and its molecular characterization. *Parasitol Res*. 2022;121(10):2887-2890. |
| *Haemaphysalis hystricis* | Kwak ML, Ng A. Detection of three new *Haemaphysalis* ticks (Acari: Ixodidae) in Singapore and their potential threat for public health, companion animals, and wildlife. *Acarologia*. 2022;62(4):927–940. |
| *Haemaphysalis juxtakochi* | Valente JDM, Silva PW, Arzua M, et al. Records of ticks (Acari: Ixodidae) on humans and distribution of spotted-fever cases and its tick vectors in Paraná State, southern Brazil. *Ticks Tick Borne Dis* 2020; 11: 101510. |
| *Haemaphysalis leachi* | Dick G, Lewis E. A rickettsial disease in east Africa transmitted by ticks (*Rhipicephalus simus* and *Haemaphysalis leachi*). *T ROY SOC TROP MED H* 1947; 41: 295-326. |
| *Haemaphysalis leporispalustris* | Hahn MB, Disler G, Durden LA, et al. Establishing a baseline for tick surveillance in Alaska: tick collection records from 1909-2019. *Ticks Tick Borne Dis* 2020; 11: 101495. |
| *Haemaphysalis longicornis* | Madison-Antenucci S, Kramer LD, Gebhardt LL, Kauffman E. Emerging tick-borne diseases. *Clin Microbiol Rev* 2020; 33: e00083-18. |
| *Haemaphysalis megaspinosa* | Seishima M, Izumi T, Oyama Z, et al. Tick bite by *Haemaphysalis megaspinosa* - first case. *Eur J Dermatol* 2000; 10: 389-91. |
| *Haemaphysalis parva* | Keskin A, Keskin A, Bursali A, Tekin S. Ticks (Acari: Ixodida) parasitizing humans in Corum and Yozgat provinces, Turkey. *Exp Appl Acarol* 2015; 67: 607-16. |
| *Haemaphysalis punctata* | Raad M, Azar D, Perotti MA. First report of the ticks Haemaphysalis punctata Canestrini et Fanzago, 1878, *Haemaphysalis parva* (Neumann, 1897) and *Dermacentor marginatus* (Sulzer, 1776) (Acari, Amblyommidae) from humans in Lebanon. *Acta Parasitol* 2020; 65: 541-5. |
| *Haemaphysalis sulcata* | Keskin A, Keskin A, Bursali A, Tekin S. Ticks (Acari: Ixodida) parasitizing humans in Corum and Yozgat provinces, Turkey. *Exp Appl Acarol* 2015; 67: 607-16. |
| *Haemaphysalis wellingtoni* | Kwak ML, Ng A. Detection of three new *Haemaphysalis* ticks (Acari: Ixodidae) in Singapore and their potential threat for public health, companion animals, and wildlife. *Acarologia*. 2022;62(4):927–940. |
| *Hyalomma aegyptium* | Keskin A, Keskin A, Bursali A, Tekin S. Ticks (Acari: Ixodida) parasitizing humans in Corum and Yozgat provinces, Turkey. *Exp Appl Acarol* 2015; 67: 607-16. |
| *Hyalomma anatolicum* | Hosseini A, Dalimi A, Abdigoudarzi M. Morphometric study on male specimens of *Hyalomma anatolicum* (Acari: Ixodidae) in West of Iran. *Iran J Arthropod Borne Dis* 2011; 5: 23-31. |
| *Hyalomma dromedarii* | Mosabah AA, Morsy TA. Tick paralysis: first zoonosis record in Egypt. *J Egypt Soc Parasitol* 2012; 42: 71-8. |
| *Hyalomma excavatum* | Keskin A, Keskin A, Bursali A, Tekin S. Ticks (Acari: Ixodida) parasitizing humans in Corum and Yozgat provinces, Turkey. *Exp Appl Acarol* 2015; 67: 607-16. |
| *Hyalomma lusitanicum* | Santos-Silva MM, Beati L, Santos AS, et al. The hard-tick fauna of mainland Portugal (Acari: Ixodidae): an update on geographical distribution and known associations with hosts and pathogens. *Exp Appl Acarol* 2011; 55: 85-121. |
| *Hyalomma marginatum* | Keskin A, Keskin A, Bursali A, Tekin S. Ticks (Acari: Ixodida) parasitizing humans in Corum and Yozgat provinces, Turkey. *Exp Appl Acarol* 2015; 67: 607-16. |
| *Hyalomma rufipes* | Medialdea-Carrera R, Melillo T, Micaleff C, Borg ML. Detection of *Hyalomma rufipes* in a recently arrived asylum seeker to the EU. *Ticks Tick Borne Dis* 2021; 12: 101571. |
| *Hyalomma truncatum* | "Horak IG, Fourie LJ, Heyne H, Walker JB, Needham G. *Rhipicephalus* Ixodid ticks feeding on humans in South Africa: with notes on preferred hosts, geographic distribution, seasonal occurrence and transmission of pathogens. *Exp Appl Acarol* 2002; 27: 113-36." |
| *Ixodes acuminatus* | Kar S, Yılmazer N, Akyıldız G, Gargili A. The human infesting ticks in the city of Istanbul and its vicinity with reference to a new species for Turkey. *Systematic and Applied Acarology*. 2017;22(12):2245–2255. |
| *Ixodes arboricola* | Špitalská E, Boldišová E, Štefanidesová K, et al. Pathogenic microorganisms in ticks removed from Slovakian residents over the years 2008-2018. *Ticks Tick Borne Dis* 2021; 12: 101626. |
| *Ixodes cookei* | Hall JE, Amrine JW Jr, Gais RD, et al. Parasitization of humans in West Virginia by *Ixodes cookei* (Acari: Ixodidae), a potential vector of Lyme borreliosis. *J Med Entomol*. 1991;28(1):186-189. |
| *Ixodes cornuatus* | Dehhaghi M, Kazemi Shariat Panahi H, Holmes EC, Hudson BJ, Schloeffel R, Guillemin GJ. Human Tick-Borne Diseases in Australia. *Front Cell Infect Microbiol.* 2019;9:3. |
| *Ixodes dentatus* | Hall JE, Amrine JW Jr, Gais RD, et al. Parasitization of humans in West Virginia by *Ixodes cookei* (Acari: Ixodidae), a potential vector of Lyme borreliosis. *J Med Entomol*. 1991;28(1):186-189. |
| *Ixodes frontalis* | Gilot B, Beaucournu JC, Chastel C. [Collecting with the flagging method and fixing on man of *Ixodes* (Trichotoixodes) *frontalis* (Panzer, 1795)]. *Parasite* 1997; 4:197-9. |
| *Ixodes hexagonus* | Faulde MK, Rutenfranz M, Hepke J, Rogge M, Görner A, Keth A. Human tick infestation pattern, tick-bite rate, and associated *Borrelia burgdorferi* s.l. infection risk during occupational tick exposure at the Seedorf military training area, northwestern Germany. *Ticks Tick Borne Dis* 2014; 5: 594-9. |
| *Ixodes marxi* | Lubelczyk C, Cahill BK, Hanson T, et al. Tick (Acari: Ixodidae) infestation at two rural, seasonal camps in Maine and Vermont. *J Parasitol.* 2010;96(2):442-443. |
| *Ixodes nipponensis* | Lee SH, Shin NR, Kim CM, et al. First identification of *Anaplasma phagocytophilum* in both a biting tick *Ixodes nipponensis* and a patient in Korea: a case report. *BMC Infect Dis* 2020; 20: 826. |
| *Ixodes pacificus* | Brown RN, Lane RS. Lyme disease in California: a novel enzootic transmission cycle of *Borrelia burgdorferi*. *Science*. 1992;256(5062):1439-1442. |
| *Ixodes pararicinus* | Kar S, Yılmazer N, Akyıldız G, Gargili A. The human infesting ticks in the city of Istanbul and its vicinity with reference to a new species for Turkey. *Systematic and Applied Acarology*. 2017;22(12):2245–2255. |
| *Ixodes pavlovskyi* | Rar V, Chicherina G, Igolkina Y, Fedorets V, Epikhina T, Tikunova N. Spectrum of Ixodidae Ticks Attacking Humans in Novosibirsk Province, Russian Siberia, and Their Association with Tick-Borne Bacterial Agents. *Pathogens*. 2025;14(4):315. |
| *Ixodes persulcatus* | Madison-Antenucci S, Kramer LD, Gebhardt LL, Kauffman E. Emerging tick-borne diseases. *Clin Microbiol Rev* 2020; 33: e00083-18. |
| *Ixodes redikorzevi* | Kassis I, Ioffe-Uspensky I, Uspensky I, Mumcuoglu KY. Human toxicosis caused by the tick *Ixodes redikorzevi* in Israel. *Isr J Med Sci* 1997; 33: 760-1. |
| *Ixodes ricinus* | Madison-Antenucci S, Kramer LD, Gebhardt LL, Kauffman E. Emerging tick-borne diseases. *Clin Microbiol Rev* 2020; 33: e00083-18. |
| *Ixodes scapularis* | Madison-Antenucci S, Kramer LD, Gebhardt LL, Kauffman E. Emerging tick-borne diseases. *Clin Microbiol Rev* 2020; 33: e00083-18. |
| *Ixodes silvanus* | Kar S, Yılmazer N, Akyıldız G, Gargili A. The human infesting ticks in the city of Istanbul and its vicinity with reference to a new species for Turkey. *Systematic and Applied Acarology.* 2017;22(12):2245–2255. |
| *Ixodes spinipalpis* | Xu G, Pearson P, Dykstra E, Andrews ES, Rich SM. Human-Biting Ixodes Ticks and Pathogen Prevalence from California, Oregon, and Washington. *Vector Borne Zoonotic Dis*. 2019 Feb;19(2):106-114. |
| *Ixodes trianguliceps* | Hubbard MJ, Baker AS, Cann KJ. Distribution of *Borrelia burgdorferi* s.l. spirochaete DNA in British ticks (Argasidae and Ixodidae) since the 19th century, assessed by PCR. *Med Vet Entomol* 1998; 12: 89-97. |
| *Ixodes turdus* | Woo IC, Baba S, Suzuki H, Kawabata M. A case of tick bite with *Ixodes turdus* Nakatsuji--a report from Japan. *J Dermatol*. 1990;17(1):56-58. |
| *Ixodes uriae* | Kar S, Yılmazer N, Akyıldız G, Gargili A. The human infesting ticks in the city of Istanbul and its vicinity with reference to a new species for Turkey. *Systematic and Applied Acarology*. 2017;22(12):2245–2255. |
| *Ixodes ventalloi* | Sanogo YO, Parola P, Shpynov S, et al. Genetic diversity of bacterial agents detected in ticks removed from asymptomatic patients in northeastern Italy. *Ann N Y Acad Sci* 2003; 990: 182-90. |
| *Ornithodoros turicata* | Busselman RE, Olson MF, Martinez V, et al. Host Bloodmeal Identification in Cave-Dwelling *Ornithodoros turicata* Dugès (Ixodida: Argasidae), Texas, USA. *Front Vet Sci*. 2021;8:639400. Published 2021 Feb 15. |
| *Rhipicephalus annulatus* | Keskin A, Keskin A, Bursali A, Tekin S. Ticks (Acari: Ixodida) parasitizing humans in Corum and Yozgat provinces, Turkey. *Exp Appl Acarol* 2015; 67: 607-16. |
| *Rhipicephalus appendiculatus* | "Horak IG, Fourie LJ, Heyne H, Walker JB, Needham G. *Rhipicephalus* Ixodid ticks feeding on humans in South Africa: with notes on preferred hosts, geographic distribution, seasonal occurrence and transmission of pathogens. *Exp Appl Acarol* 2002; 27: 113-36" |
| *Rhipicephalus bursa* | Keskin A, Keskin A, Bursali A, Tekin S. Ticks (Acari: Ixodida) parasitizing humans in Corum and Yozgat provinces, Turkey. *Exp Appl Acarol* 2015; 67: 607-16. |
| *Rhipicephalus evertsi* | "Horak IG, Fourie LJ, Heyne H, Walker JB, Needham G. *Rhipicephalus* Ixodid ticks feeding on humans in South Africa: with notes on preferred hosts, geographic distribution, seasonal occurrence and transmission of pathogens. *Exp Appl Acarol* 2002; 27: 113-36." |
| *Rhipicephalus gertrudae* | Horak IG, Fourie LJ, Heyne H, Walker JB, Needham GR. Ixodid ticks feeding on humans in South Africa: with notes on preferred hosts, geographic distribution, seasonal occurrence and transmission of pathogens. *Exp Appl Acarol*. 2002;27(1-2):113-136. |
| *Rhipicephalus microplus* | Szabó MPJ, Martins TF, Barbieri ARM, et al. Ticks biting humans in the Brazilian savannah: attachment sites and exposure risk in relation to species, life stage and season. *Ticks Tick Borne Dis* 2020; 11: 101328. |
| *Rhipicephalus pusillus* | Santos-Silva MM, Beati L, Santos AS, et al. The hard-tick fauna of mainland Portugal (Acari: Ixodidae): an update on geographical distribution and known associations with hosts and pathogens. *Exp Appl Acarol* 2011; 55: 85-121. |
| *Rhipicephalus rossicus* | BORODIN VP, KHLIUSTOVA AI, KOROLEVA AP, SAMSONOVA AP, SPITSYN NA. Dva sluchaia zabolevanii tuliaremii ot ukusa kleshchei *Rhipicephalus rossicus* Jakim. et K. Jakim [Two cases of tularemia caused by bite of tick *Rhipicephalus rossicus* Jakim. et K. Jakim]. *Zh Mikrobiol Epidemiol Immunobiol*. 1956;27(9):49-51. |
| *Rhipicephalus sanguineus* | Madison-Antenucci S, Kramer LD, Gebhardt LL, Kauffman E. Emerging tick-borne diseases. *Clin Microbiol Rev* 2020; 33: e00083-18. |
| *Rhipicephalus turanicus* | Keskin A, Keskin A, Bursali A, Tekin S. Ticks (Acari: Ixodida) parasitizing humans in Corum and Yozgat provinces, Turkey. *Exp Appl Acarol* 2015; 67: 607-16. |

Table S16: Poultry infested with ticks

^a^ indicates pathogens detected in birds

| Bird order | Tick species | Pathogens detected in ticks | Nation | Reference ID |
| --- | --- | --- | --- | --- |
| With pathogen detected |  |  |  |  |
| Galliformes | *Argas persicus* | *Borrelia anserina*^a^ | Iran | 591 |
| Galliformes | *Argas persicus* | *Borrelia anserina*, *Rickettsia* sp., *Coxiella burnetii* | Algeria | 39 |
| Galliformes | *Argas persicus* | *Coxiella burnetii* | Pakistan | 548 |
| Galliformes | *Haemaphysalis wellingtoni* | *Coxiella* sp. | Thailand | 9 |
| Struthioniformes | *Hyalomma* sp*.* | *Orthonairovirus haemorrhagiae* | South Africa | 398 |
| Galliformes | *Rhipicephalus sanguineus* | *Ehrlichia* sp., *Bartonella* sp., *Rickettsia* sp. | Guatemala | 753 |
| Galliformes | *Haemaphysalis wellingtoni* | *Rickettsia felis* | Malaysia | 445 |
| Galliformes | *Argas persicus* | *Rickettsia hoogstraalii* | China | 298 |
| Galliformes | *Haemaphysalis longicornis* | Severe fever with thrombocytopenia syndrome virus | China | 426 |
| Unidentified | *Argas persicus* | *Borrelia anserina* | Pakistan | 507 |
| Without pathogen detected |  |  |  |  |
| Anseriformes | *Argas persicus* | NA | Algeria | 20 |
| Anseriformes | *Argas persicus* | NA | Iran | 594 |
| Anseriformes, Galliformes | *Argas persicus* | NA | Pakistan | 507 |
| Anseriformes, Galliformes | *Haemaphysalis concinna* | NA | China | 768 |
| Columbiformes | *Argas hermanni* | NA | Iran | 565 |
| Columbiformes | *Argas persicus* | NA | China | 772 |
| Columbiformes, Galliformes | Unidentified | NA | Iran | 547 |
| Galliformes | *Ornithodoros* sp. | NA | Tanzania | 415 |
| Galliformes | *Amblyomma* sp. | NA | Mexico | 558 |
| Galliformes | *Amblyomma variegatum* | NA | French West Indies | 170 |
| Galliformes | *Argas persicus* | NA | Ethiopia | 14 |
| Galliformes | *Argas persicus* | NA | Algeria | 20 |
| Galliformes | *Argas persicus* | NA | Zimbabwe | 372 |
| Galliformes | *Argas persicus* | NA | Ethiopia | 483 |
| Galliformes | *Argas persicus* | NA | Pakistan | 509 |
| Galliformes | *Argas persicus* | NA | Algeria | 561 |
| Galliformes | *Argas persicus* | NA | Tunisia | 574 |
| Galliformes | *Argas persicus* | NA | Tanzania | 629 |
| Galliformes | *Argas persicus* | NA | Kenya | 632 |
| Galliformes | *Argas persicus* | NA | Pakistan | 678 |
| Galliformes | *Argas persicus* | NA | Amhara | 533 |
| Galliformes | *Argas persicus* | NA | China | 769, 772 |
| Galliformes | *Haemaphysalis bispinosa* | NA | Thailand | 9 |
| Galliformes | *Haemaphysalis intermedia*, *Haemaphysalis turturis*, *Haemaphysalis spinigera*, *Haemaphysalis aculeata* | NA | Sri Lanka | 607 |
| Galliformes | *Amblyomma testudinarium*, *Amblyomma integrum* | NA | Sri Lanka | 607 |
| Galliformes | *Dermacentor auratus* | NA | Sri Lanka | 607 |
| Galliformes | *Haemaphysalis wellingtoni* | NA | Malaysia | 370 |
| Galliformes | *Ixodes pacificus* | NA | Pakistan | 622 |
| Galliformes | Unidentified | NA | Kenya | 597 |
| Passeriformes | *Argas reflexus* | NA | Iran | 573 |
| Unidentified | *Argas persicus* | NA | Pakistan | 518 |
| Unidentified | *Argas persicus* | NA | Iran | 519 |

Table S17: Non-native ticks along migratory bird flyways

| Non-native ticks | Study site | Habitat | Latitude | Longtitude | Reference ID | Original distribution area of the tick | Potential flyways | Evidence consistency |
| --- | --- | --- | --- | --- | --- | --- | --- | --- |
| *Ornithodoros capensis* | Brazil | island | -24.48360 | -46.67540 | 589 | the tropical marine regions | ATLANTIC AMERICAS | confirmed |
| *Amblyomma calcaratum* | USA | coastline | 29.75984 | -93.67046 | 219 | Tropical South America | ATLANTIC AMERICAS | confirmed |
| *Amblyomma longirostre* | USA | coastline | 29.75984 | -93.67046 | 219 | Tropical South America | ATLANTIC AMERICAS | suspected |
| *Amblyomma nodosum* | USA | coastline | 29.75984 | -93.67046 | 219 | Tropical South America | ATLANTIC AMERICAS | confirmed |
| *Haemaphysalis juxtakochi* | USA | coastline | 29.75984 | -93.67046 | 219 | Tropical South America | ATLANTIC AMERICAS | confirmed |
| *Ixodes arboricola* | Egypt | na | 30.93369 | 29.58073 | 176 | the Mediterranean region of Europe | BLACK SEA, MEDITERRANEAN | confirmed |
| *Hyalomma marginatum* | Hungary | park | 47.72633 | 18.90817 | 73 | Southern Europe, North Africa, and Western Asia | BLACK SEA, MEDITERRANEAN | suspected |
| *Argas arboreus* | Israel | na | 31.56913 | 34.69384 | 285 | Northeastern Africa and the Nile River Basin | BLACK SEA, MEDITERRANEAN | confirmed |
| *Amblyomma marmoreum* | Italy | island | 40.78640 | 13.41530 | 494 | the Central and Southern African region | BLACK SEA, MEDITERRANEAN | confirmed |
| *Amblyomma variegatum* | Italy | island | 40.79625 | 13.43056 | 1 | Tropical Africa | BLACK SEA, MEDITERRANEAN | confirmed |
| *Amblyomma variegatum* | Italy | island | 40.78640 | 13.41530 | 494 | Tropical Africa | BLACK SEA, MEDITERRANEAN | confirmed |
| *Argas africolumbae* | Italy | island | 40.79625 | 13.43056 | 70 | Sub-Saharan Africa | BLACK SEA, MEDITERRANEAN | confirmed |
| *Argas vulgaris* | Italy | island | 40.79625 | 13.43056 | 1 | Russia and the Asian regions | BLACK SEA, MEDITERRANEAN | confirmed |
| *Hyalomma rufipes* | Italy | island | 40.79625 | 13.43056 | 1 | Sub-Saharan Africa | BLACK SEA, MEDITERRANEAN | confirmed |
| *Hyalomma rufipes* | Italy | island | 40.78640 | 13.41530 | 494 | North Africa and sub-Saharan Africa。 | BLACK SEA, MEDITERRANEAN | confirmed |
| *Hyalomma truncatum* | Italy | island | 40.79625 | 13.43056 | 1 | Tropical Africa | BLACK SEA, MEDITERRANEAN | confirmed |
| *Hyalomma truncatum* | Italy | island | 40.78640 | 13.41530 | 494 | Tropical Africa | BLACK SEA, MEDITERRANEAN | confirmed |
| *Ornithodoros coniceps* | Malta | island | 35.93750 | 14.37540 | 690 | Africa and the islands of Western Europe | BLACK SEA, MEDITERRANEAN | confirmed |
| *Amblyomma marmoreum* | Malta | island | 35.87833 | 14.44629 | 22 | Tropical Africa | BLACK SEA, MEDITERRANEAN | confirmed |
| *Hyalomma marginatum* | Malta | island | 35.87833 | 14.44629 | 22 | Southern Europe, North Africa, and Western Asia | BLACK SEA, MEDITERRANEAN | suspected |
| *Hyalomma rufipes* | Malta | island | 35.87833 | 14.44629 | 22 | North Africa and sub-Saharan Africa。 | BLACK SEA, MEDITERRANEAN | confirmed |
| *Ixodes cumulatimpunctatus* | Malta | island | 35.87833 | 14.44629 | 22 | Tropical Africa | BLACK SEA, MEDITERRANEAN | confirmed |
| *Hyalomma rufipes* | Slovakia | mountain | 49.15058 | 18.00802 | 397 | North Africa and sub-Saharan Africa。 | BLACK SEA, MEDITERRANEAN | confirmed |
| *Ixodes eldaricus* | Turkey | wetland ecosystems | 41.63333 | 36.08333 | 46 | the Transcaucasian region | BLACK SEA, MEDITERRANEAN | confirmed |
| *Ixodes festai* | Turkey | wetland ecosystems | 41.63333 | 36.08333 | 46 | Southern Europe and North Africa | BLACK SEA, MEDITERRANEAN | confirmed |
| *Hyalomma marginatum* | Finland | island | 62.60380 | 30.39450 | 4 | Southern Europe, North Africa, and Western Asia | EAST ATLANTIC | suspected |
| *Hyalomma marginatum* | Finland | island | 59.84962 | 21.61397 | 4 | Southern Europe, North Africa, and Western Asia | EAST ATLANTIC | suspected |
| *Ixodes frontalis* | Finland | island | 59.83330 | 19.91670 | 635 | Sub-Saharan Africa | EAST ATLANTIC | confirmed |
| *Hyalomma rufipes* | Norway | coastline | 59.04927 | 10.89888 | 73 | Tropical Africa | EAST ATLANTIC | confirmed |
| *Ixodes kaiseri* | Norway | na | 40.63546 | 22.95348 | 282 | Central Europe, Eastern Europe, and the Black Sea region | EAST ATLANTIC | confirmed |
| *Hyalomma lusitanicum* | Portugal | mixed forest | 40.22220 | -8.44375 | 19 | the Western Mediterranean region | EAST ATLANTIC | suspected |
| *Hyalomma marginatum* | Portugal | mixed forest | 40.22220 | -8.44375 | 19 | Southern Europe, North Africa, and Western Asia | EAST ATLANTIC | suspected |
| *Ixodes arboricola* | Portugal | mixed forest | 40.22220 | -8.44375 | 19 | the Mediterranean region of Europe | EAST ATLANTIC | confirmed |
| *Amblyomma americanum* | Canada | na | 53.45000 | -113.00000 | 606 | the central and southern parts of the Americas | CENTRAL AMERICAS | suspected |
| *Amblyomma dissimile* | Canada | na | 43.61780 | -79.34250 | 605 | Southern Brazil | CENTRAL AMERICAS | confirmed |
| *Amblyomma longirostre* | Canada | na | 42.58000 | -80.41000 | 606 | Tropical South America | CENTRAL AMERICAS | suspected |
| *Amblyomma maculatum* | Canada | na | 44.03000 | -77.29000 | 606 | the southeastern United States | CENTRAL AMERICAS | suspected |
| *Amblyomma rotundatum* | Canada | lake | 42.55000 | -80.05000 | 604 | Southern Brazil | CENTRAL AMERICAS | confirmed |
| *Ixodes minor* | Canada | na | 43.70000 | -79.41670 | 608 | the Central American region | CENTRAL AMERICAS | confirmed |
| *Amblyomma varium* | Peru | forest park | -3.94392 | -73.60654 | 204 | Tropical South America | CENTRAL AMERICAS | confirmed |
| *Ixodes auritulus* | USA | coastline | 28.30030 | -80.70120 | 667 | the North American west coast | CENTRAL AMERICAS | confirmed |
| *Ornithodoros capensis* | Japan | island | 30.46667 | 140.30000 | 283 | Hawaii and the mainland United States | EAST ASIA, AUSTRALASIA | confirmed |
| *Ornithodoros capensis* | Korea | island | 34.50000 | 128.83330 | 588 | the tropical marine regions | EAST ASIA, AUSTRALASIA | confirmed |
| *Ornithodoros sawaii* | Korea | island | 36.68330 | 126.10000 | 588 | the North Pacific coast | EAST ASIA, AUSTRALASIA | confirmed |
| *Ornithodoros sawaii* | Korea | island | 34.06670 | 125.11670 | 590 | the tropical regions | EAST ASIA, AUSTRALASIA | confirmed |
| *Ornithodoros sawaii* | Korea | island | 34.78330 | 125.80000 | 602 | the North Pacific coast | EAST ASIA, AUSTRALASIA | confirmed |
| *Haemaphysalis concinna* | Korea | island | 33.36700 | 126.53300 | 28 | Southeast Asia | EAST ASIA, AUSTRALASIA | confirmed |
| *Haemaphysalis flava* | Korea | island | 33.36700 | 126.53300 | 30 | Southeast Asia and the tropical Pacific region | EAST ASIA, AUSTRALASIA | confirmed |
| *Haemaphysalis formosensis* | Korea | island | 33.36700 | 126.53300 | 28 | Southeast Asia | EAST ASIA, AUSTRALASIA | confirmed |
| *Ixodes turdus* | Korea | island | 33.36700 | 126.53300 | 30 | Southeast Asia and the tropical Pacific region | EAST ASIA, AUSTRALASIA | confirmed |
| *Ixodes uriae* | Korea | island | 34.68330 | 125.18330 | 592 | the polar regions | EAST ASIA, AUSTRALASIA | confirmed |
| *Ixodes uriae* | Norway | na | 70.66667 | 30.33333 | 289 | the polar regions | EAST ATLANTIC | suspected |
| *Ornithodoros capensis* | Coiba | island | 7.65340 | -81.72720 | 689 | the tropical marine regions | PACIFIC AMERICAS | confirmed |

**Definition** (Allochthonous ticks):
We defined a tick occurrence as allochthonous (non-native) when the original study explicitly described the tick as a first local record or non-native in the reported area and provided a clear species identification approach (e.g., morphological keys and or molecular markers).

**Confidence grading** (confirmed vs suspected)**:**

Each candidate record was cross-validated within the first administrative level of the reported location using GBIF occurrence data (accessed Oct 2025) and a supplementary PubMed search for earlier regional reports. Records were classified as confirmed when no prior occurrence of the same tick species was found in GBIF for that first-level administrative unit or ecologically comparable neighbouring areas, and no earlier literature record was retrieved. Records were classified as suspected when GBIF or the supplementary literature indicated prior records in the reported area, or when the reported location fell within the species’ commonly recorded range, making the “first-record/non-native” claim uncertain.
